# Multiscale structure of chromatin condensates explains phase separation and material properties

**DOI:** 10.1126/science.adv6588

**Published:** 2025-12-04

**Authors:** Huabin Zhou, Jan Huertas, M. Julia Maristany, Kieran Russell, June Ho Hwang, Run-Wen Yao, Nirnay Samanta, Joshua Hutchings, Ramya Billur, Momoko Shiozaki, Xiaowei Zhao, Lynda K. Doolittle, Bryan A. Gibson, Andrea Soranno, Margot Riggi, Jorge R. Espinosa, Zhiheng Yu, Elizabeth Villa, Rosana Collepardo-Guevara, Michael K. Rosen

**Affiliations:** 1 Department of Biophysics, Howard Hughes Medical Institute, UT Southwestern Medical Center, Dallas, TX, USA; 2 Yusuf Hamied Department of Chemistry, University of Cambridge, Cambridge CB2 1EW, UK; 3 Department of Genetics, University of Cambridge, Cambridge CB2 3EH, UK; 4 Maxwell Centre, Cavendish Laboratory, Department of Physics, University of Cambridge, Cambridge CB3 0HE, UK; 5 Department of Biochemistry and Molecular Biophysics, Washington University in St. Louis, St. Louis, MO, USA; 6 Center for Biomolecular Condensates, Washington University in St. Louis, St Louis, MO, USA; 7 School of Biological Sciences, University of California San Diego, La Jolla, CA, USA; 8 Department of Cell and Molecular Biology, St. Jude Children’s Research Hospital, Memphis, TN, USA; 9 Janelia Research Campus, Howard Hughes Medical Institute, Ashburn, VA, USA; 10 Max Planck Institute for Biochemistry, Martinsried/Munich D-82152, Germany; 11 Howard Hughes Medical Institute, University of California San Diego, La Jolla, CA, USA; 12 Marine Biological Laboratory Chromatin Collaborative, Marine Biological Laboratory, Woods Hole, MA, USA

## Abstract

The structure and interaction networks of molecules within biomolecular condensates are poorly understood. Using cryo-electron tomography and molecular dynamics simulations, we elucidated the structure of phase separated chromatin condensates across scales, from individual amino acids to network architecture. We found that internucleosomal DNA linker length controls nucleosome arrangement and histone tail interactions, shaping the structure of individual chromatin molecules within and outside condensates. This structural modulation determines the balance between intra- and intermolecular interactions, which governs the molecular network, thermodynamic stability, and material properties of chromatin condensates. Mammalian nuclei contain dense clusters of nucleosomes whose non-random organization is mirrored by the reconstituted condensates. Our work explains how the structure of individual chromatin molecules determines physical properties of chromatin condensates and cellular chromatin organization.

Cells are organized on length scales from nanometers to micrometers. Biomolecular condensates concentrate molecules into discrete foci without surrounding membranes, exemplifying how nanoscale molecular interactions can produce micron scale cellular organization. Many condensates appear to form through assembly and concomitant phase separation of multivalent macromolecules (1–3). The structure and dynamics of individual molecules, and of their multivalency-enabled interaction networks, strongly influence the macroscopic physical properties of condensates (4–6). Physical properties, along with solution environment and composition define the biochemical and cellular functions of condensates (7–16). These features of condensates are altered in diseases and have been suggested as therapeutic targets (17, 18). An important goal in biology and human health is to understand the relationships between molecular structure/dynamics, network architecture/dynamics and function of condensates.

Molecules within condensates are often conformationally flexible and interact weakly and transiently, producing complex distributions of interconverting states. Nuclear magnetic resonance spectroscopy has revealed structural ensembles and residue-specific interactions of intrinsically disordered regions of proteins (IDRs) within condensates (19–21). But these studies have not strongly informed on the higher order organization of these elements within condensates. On micrometer length scales, cryo-electron tomography (cryo-ET) has been used to characterize the morphology of condensates (22–29), but has not connected meso-scale features to molecular interactions that form the compartments. Computational work has sought to bridge molecular interactions to the network architecture of intrinsically disordered protein (IDP) condensates, providing insight into viscoelasticity and surface tension (5, 30–32). But these models are difficult to test experimentally due to the challenge of directly visualizing molecules and interactions inside condensates. Because of these complexities, we still lack a complete understanding from molecular structure and interactions to meso-scale organization and dynamics for any condensate.

Phase separation is thought to play an important role in the organization of eukaryotic chromatin, enabling compaction necessary for storage in the nucleus while retaining dynamics necessary for function (33–43). Chromatin fibers are arrays of nucleosomes, structural units containing ~147 base pairs of DNA wrapped around an octamer of histone proteins (44), connected by DNA linkers of diverse lengths. Nucleosome arrays compact along their lengths and interact with one another (45), creating higher order cellular structures ranging from 10-100 nm clusters (46–48) to 100-300 nm domains (49, 50). The compaction and dynamics of nucleosomes are highly regulated, affecting nuclear processes including transcription (51–53), DNA replication and DNA repair (54). Interactions between biochemically reconstituted nucleosome arrays can drive salt-dependent phase separation, dependent on the disordered tails of histones (33, 35, 55–60). Post-translational modifications of histone tails, including acetylation and methylation, can regulate phase separation and biological functions (33, 38, 57). Phase separation is also modulated by the length of inter-nucleosome DNA linkers; the drive to phase separate shows an oscillatory pattern as linkers are extended, stronger for 10N+5 base pairs (integer N) and weaker for 10N base pairs (33). These behaviors, along with genomic data and modeling, suggest that phase separation of the chromatin polymer plays a role in genome organization in vivo (33, 35, 57–61).

Numerous studies have examined the structure of native chromatin fibers using different modalities of light and electron microscopy (46, 49, 62–66), as well as chemical crosslinking (67, 68). These have revealed that chromatin inside cells/nuclei consists mostly of irregular conformations, although small segments of ordered, stacked groups of nucleosomes have also been observed (62, 65, 69, 70). Most of these studies focused on the conformations of individual fibers, rather than the structural basis of interactions between them. However, in one study pairwise inter-nucleosome interactions revealed a wide distribution of packing geometries (65). The relationship between the conformations, interactions and networking of fibers to the density and material properties of chromatin in cells is not known.

Here we integrated cryo-ET, computer simulations and light microscopy to understand mechanisms of phase separation and physical properties of synthetic and native chromatin. We found that nucleosome linker length controls salt-dependent conformations and histone tail interactions of chromatin fibers. These properties dictate the strength and number of interactions between fibers in condensates, which then define the network structure of the condensates. These physical features explain the driving force for chromatin phase separation as well as viscoelastic properties of chromatin condensates. Dense, 100-300 nm clusters of native chromatin in situ show a similar, non-random distribution of inter-nucleosome contacts as one class of synthetic chromatin. Together, our studies provide an understanding of chromatin phase separation from nanometers to micrometers, and suggest that certain reconstituted chromatin condensates may functionally mimic important features of native chromatin.

## Distinct phase separation propensity and dynamics of 25 bp and 30 bp chromatin

**Figure 1.**
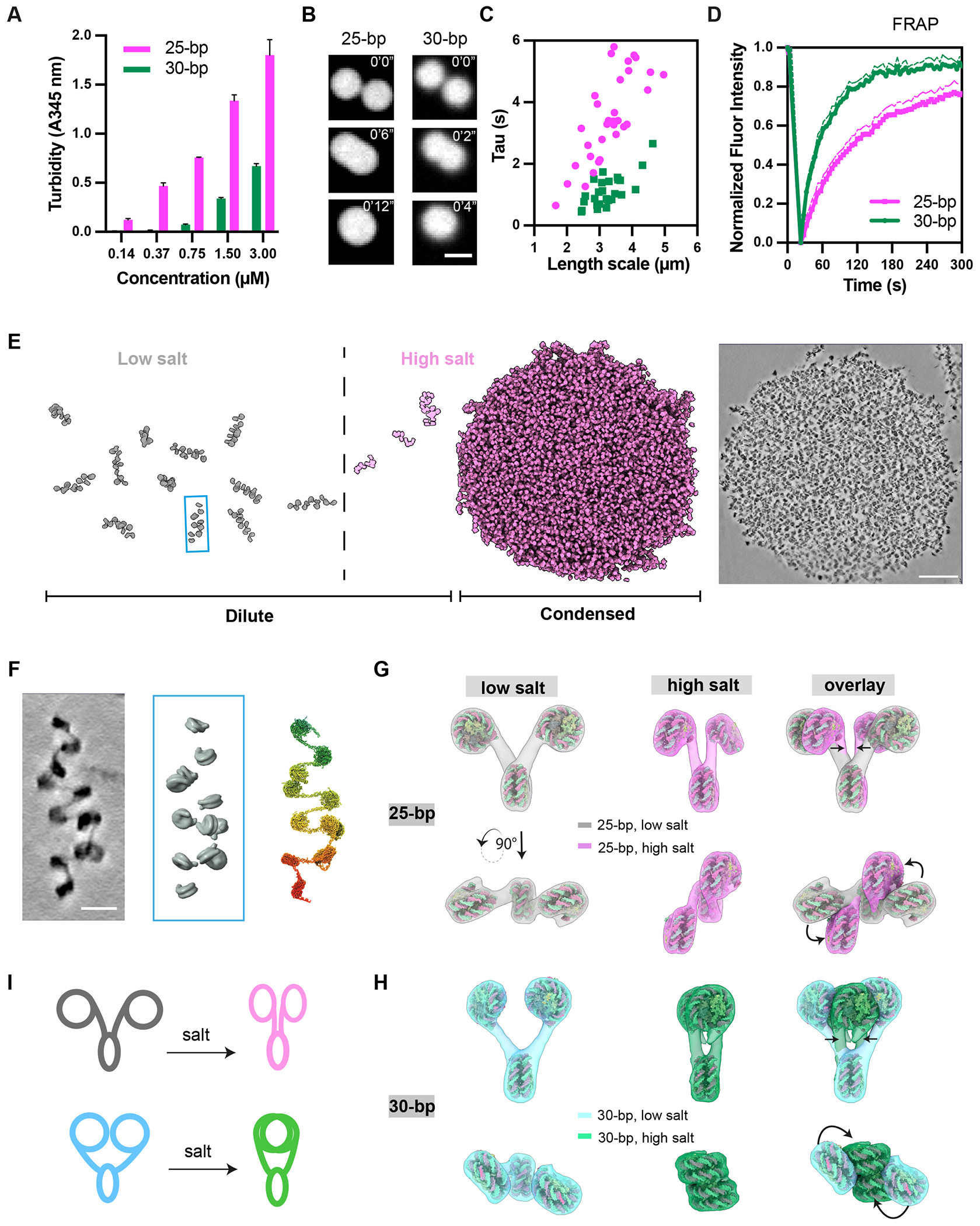
Linker length determines condensate properties and chromatin fiber structure. (A) Turbidity (absorption at 345 nm) of chromatin solutions at the indicated nucleosome concentrations. Error bars indicate ± standard deviation of three measurements. (B) Time-lapse imaging of droplet fusion in 25 bp and 30 bp chromatin immediately after inducing phase separation. Scale bar is 3 μm. (C) Timescale of fusion for 25 bp and 30 bp chromatin droplets of different sizes. (D) Fluorescence Recovery After Photobleaching (FRAP) results for 25 bp and 30 bp chromatin. Error bars indicate ± standard deviation of 20 measurements. (E) Schematic of the compaction and phase separation of chromatin fibers as salt concentration increases (left and middle panels) and visualized by cryo-ET (right panel), where scale bar is 100 nm. Boxed array derives from dilute phase cryo-ET image in panel F. (F) Schematic of the computational approach used to reconstruct histone tails on a single chromatin array visualized by cryo-ET in low salt. From left to right, images show: maximum projection of chromatin density in a denoised cryo-ET tomogram; nucleosome models extracted from the density (corresponding to fiber boxed in panel E; scale bars are 20 nm); higher resolution fiber computationally reconstructed using molecular dynamics simulations steered according to nucleosome positions in the cryo-ET data.(G-H) Models based on average structural parameters of chromatin with 25 bp (G) and 30 bp (H) linkers in the dilute phase at low (left) and high (middle) salt concentrations. Right panels show overlays of the two conditions. (I) Schematic representation of the conformational changes induced by salt in 25 bp and 30 bp chromatin.

We generated dodecameric nucleosome arrays based on the Widom 601 sequence (71, 72), with either 25 bp or 30 bp internucleosomal DNA linkers (25 bp or 30 bp chromatin, respectively). As previously reported (33), 25 bp chromatin phase separates at lower concentration and at lower ionic strength than 30 bp chromatin (Fig. 1A). The 25 bp chromatin droplets fuse more slowly than 30 bp chromatin droplets (Fig. 1B, C), and recover more slowly in partial droplet fluorescence recovery after photobleaching (FRAP) experiments (Fig. 1D). We sought to understand these differences in phase separation propensity and material properties using cryo-ET and simulations (Fig. 1E).

## Salt differentially compacts 25 bp and 30 bp chromatin

We collected cryo-ET data in low- and high salt conditions (0 and 150 mM potassium acetate, respectively) for solutions of 25 bp and 30 bp chromatin. In low salt, molecules are monomeric and homogeneously distributed in solution, while in high salt they separate into dense and dilute phases. In low salt conditions we could readily identify nucleosomes and trace their connectivity in individual chromatin fibers in cryo-ET tomograms (Fig. 1F, S1, Movie S1, 2).

We first examined how increasing salt affects the structure of individual nucleosome arrays. Numerous studies have shown that salt causes compaction and oligomerization of chromatin fibers (73–79), although most studies have not revealed the underlying structural details (but see (80) and (81) for recent exceptions). For each traced fiber we measured the distance between the centers of mass, D, and the dihedral angle between planes, para, for all pairs of nucleosomes N and N+2 (N represents sequential position). We also measured the dihedral angle between the planes of adjacent (N, N+1) nucleosomes, α (Fig. S2A–D). To assist in visualizing these geometric features, in each system/condition we reconstructed an average di-nucleosome, and then combined two such pairs to build an average tri-nucleosome model (Fig. S2E), shown in Figures 1G and H.

In low salt, the 25 bp and 30 bp chromatin have similarly large values of D (Fig. S2D), indicating that semi-adjacent nucleosomes are splayed apart from one other; para values are ~45° in both cases. The average α angle for the two arrays differs by nearly 180° (α ~ 230° and 50°, for 25 bp and 30 bp, respectively, Fig. S2B), reflecting the difference in phasing of successive nucleosomes imparted by the 10 bp/turn helical pitch of the linker DNA. Because of this difference, in 25 bp chromatin the N and N+2 nucleosomes face away from each other (Fig. 1G, left), whereas in 30 bp chromatin they face toward each other (Fig. 1H, left).

Increased salt reduces long-range electrostatic repulsion in the chromatin DNA (77), rearranging and compacting the arrays (Fig. S1D). In both types, D decreases appreciably (Fig. S2D), as does para (Fig. S2C). In the 25 bp chromatin, nucleosomes N and N+2 remain unstacked with their faces exposed to solvent (Fig. 1G), and become roughly parallel to the intervening N+1 nucleosome as shown by the decrease in α (to ~ 200°, Fig. 1G,I,S2B). The changes to D and para are larger in the 30 bp chromatin, due to face-to-face stacking of the N and N+2 nucleosomes, which become oriented approximately perpendicular to the N+1 nucleosome (Fig. 1H–I) as indicated by the large increase in α (to ~80°, Fig. S2B), similar to observations on a 40 bp tetra-nucleosome arrays (81). These changes result in greater compaction of 30 bp chromatin than 25 bp chromatin (Fig. S2F). We note that intra-nucleosome stacking in 30 bp chromatin has been predicted by multiple computer simulations (82–84) and observed in crystal structures (85) and single-particle cryo-electron microscopy reconstructions (86) of arrays with linkers of 20, 30 or 40 base pairs (Fig. S2G). Computational studies suggest that this conformation is stabilized by face-to-face nucleosome contacts, which are the most energetically favorable nucleosome packing geometries, followed by face-to-side and side-to-side (83, 87).

## Salt differentially reorganizes histone tail interactions

**Figure 2.**
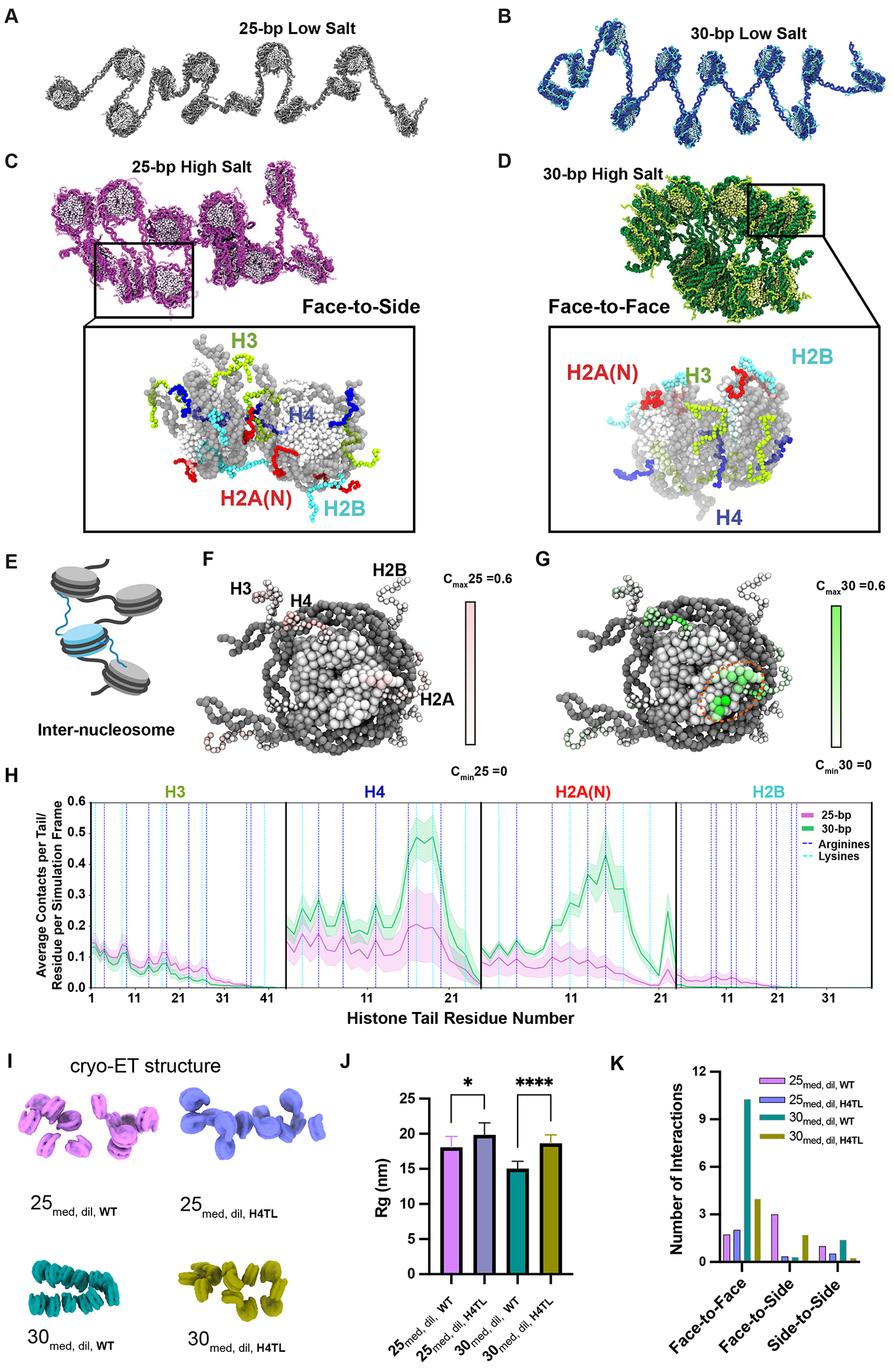
Different histone tail interactions in 25 bp and 30 bp chromatin in the dilute phase. (A-B) Schematic representations 25 bp (A) and 30 bp (B) chromatin arrays in low salt derived from cryo-ET and computationally extended to the chemical-specific model. DNA beads are represented in dark grey (A) and dark blue (B), histone protein beads are in light grey (A) and light blue (B) (C-D) As in (A-B) for chromatin in high-salt. In (C) DNA beads are dark pink (C) and dark green (D), histone protein beads are light pink (C) and light green (D). Insets show a pair of nucleosomes interacting face-to–side (C) and face-to-face ((D) via histone tails. Histone core beads are white and DNA beads are light grey. Histone tail beads are colored as: H3 green, H4 blue, H2A-N terminal red, and H2B cyan. (E) Schematic representation of inter-nucleosome histone tail interactions. (F-G). Representative structures of a nucleosome from 25 bp (F) and 30 bp (G) chromatin simulations illustrating average contact frequency of each residue; histone core and tails are represented by larger and smaller beads, respectively. Residues color-coded by the total number of inter-nucleosome contacts (averaged over 400 simulation frames, see Table S1 for n) made by each amino acid in histones (H3, H4, H2A(N), and H2B) of one nucleosome with DNA (nucleosomal and linker) and histones (core and tails) of neighboring nucleosomes. Acidic patch in G shown by dashed orange oval. (H) Inter-nucleosome contacts (average per residue per simulation frame) made by each amino acid in the histone of one nucleosome and the DNA and histones of neighboring nucleosomes (simulation details in F-G legend above) 25 bp fibers pink, 30 bp fibers green). Blue, cyan and orange vertical lines show positions of lysines, arginines and acidic patch residues, respectively. Shading indicates standard deviation from the mean. (I) Representative nucleosome arrays assigned in cryo-ET tomograms of 25 bp and 30 bp arrays (medium salt, dilute phase) assembled with either wild-type histone octamers (25_med,dil_,WT, 30_med,dil_,WT) or H4 tail deletion octamers (25_med,dil_,H4TL, 30_med,dil_,H4TL). (J) Radius of gyration of indicated nucleosome arrays. (K) Intramolecular interactions per 12-mer array, classified as face-to-face, face-to-side, side-to-side.

To gain insight into the histone tail interactions driving salt-dependent chromatin compaction, we combined experimental cryo-ET data with computational simulations using a chemically specific coarse-grained model (88), which represents chromatin at amino acid and nucleotide resolution (Fig. 2A–D, Table S1). We simulated a series of arrays observed in the cryo-ET tomograms, restraining the nucleosome geometric centers to their experimental positions in low (Fig. 2A–B) and high salt (Fig. 2C–D). The simulations generate structural ensembles of the histone tails, revealing the probabilities of different histone-tail conformational states. We quantified the number of intra- and inter-nucleosome contacts mediated by each tail (Fig. S3A). This analysis identifies the most frequent interactions within nucleosome pairs arranged according to the cryo-ET data. Although interaction statistics do not directly report on energies, our analyses show that the most frequent tail interactions arise from energetically favorable pairs—lysines and arginines contacting negatively charged histone residues and DNA (Fig. S3B).

The simulations show that in low salt, the histone tails in both chromatin types make almost exclusively intra-nucleosome interactions with DNA (Fig. 2A–B; S3C–D), as in previous simulations (89). In high salt, the tails partially release from their own nucleosomes (Fig. S3C) and instead contact the DNA and histones of neighbors (Fig. 2C–E and Fig. S3C). The nature of these interactions is governed by the conformation of the fiber, and by the location of each tail on the nucleosome core: H4 and H2A(N) emerge from the nucleosome face, whereas H3 and H2B extend from the nucleosome edge (Fig. 2C–D and Fig. S3E).

The cryo-ET data show that in high salt, 25 bp arrays in the dilute phase adopt diverse conformations, with nucleosomes displaying a broad range of distances and orientations relative to their neighbors (Fig.S1D and S2B–D). In the simulations, this range of inter-nucleosome configurations coincides with heterogeneous interactions involving principally histone H2A, H3 and H4 tails (Fig. 2C, E, F, H). This heterogeneity is evident in the larger variance in contacts per residue compared to 30 bp arrays (Fig. 2H). The H3 tail is ideally positioned for face-to-side and side-to-side nucleosome contacts, while the H4 and H2A(N) tails facilitate face-to-side interactions and, less frequently, face-to-face contacts (Fig. 2C). Consistent with the diverse tail-behaviors of 25 bp chromatin, simulations indicate that the free energy landscape of a 25-bp tetranucleosome is characterized by multiple competing low-lying minima (90).

The 30 bp arrays are different in that the prevalence of intramolecular face-to-face stacking decreases the heterogeneity of tail interactions (reducing their standard deviations in Fig. 2H), and amplifies the contributions of the H4 and H2A(N) tails, whose positions facilitate stabilization of face-to-face stacking (Fig. 2D–E and S3E). The H3 and H2B tails are less involved due to their peripheral locations. The H4 and H2A(N) tails make appreciably more inter-nucleosome contacts in 30 bp chromatin than in 25 bp chromatin (Fig. 2G, H). The region in H4 from K16 to R19 makes the highest number of contacts (Fig. 2H and S4A), in agreement with observed decompaction of chromatin upon H4K16 acetylation or mutation of these residues (91–93). Residues in and adjacent to the H2A and H2B acidic patch (H2A residues Y57, E61, E64, L65, N68, R71, D72, N89-E92; H2B residues E102, E110 plus L42-D47) formed more interactions in 30 bp chromatin compared to 25 bp chromatin (Fig. 2F–H), primarily due to interactions with the H4-tail basic patch (94–97).

The histone tail contact distributions of 25 bp and 30 bp chromatin are consistent with reported biochemical studies of chromatin compaction (Tables S2–3). Previous simulations performed without experimental restraints have also revealed that histone tail contacts are dependent on solution conditions, linker DNA length, and inter-nucleosome orientations (84, 89, 98–100), as observed here.

Our combined data and simulations show that increased salt concentration drives compaction of nucleosome arrays concomitant with formation of many new inter-nucleosome contacts. For 25 bp chromatin, DNA geometry and diverse contacts of the H3, H4 and H2A(N) histone tails produce heterogeneous structures with many nucleosome faces exposed to solvent. In contrast, for 30 bp chromatin, DNA geometry and interactions of histone H4 and H2A(N) tails favor intramolecular stacking of nucleosomes. As described below, these differences in configuration strongly influence the ability of molecules to interact with neighbors, with important consequences on phase separation and condensate material properties.

## Histone H4 tail deletion differentially affects 25 bp and 30 bp chromatin conformations

To test the predicted differential contributions of the H4 tail to the 25 bp and 30 bp chromatin structures we assembled both array types with octamers lacking the H4 tail and determined their structures in 60 mM potassium acetate buffer using cryo-ET (Fig 2I–K, S4B–D). Deleting the H4 tail increased radius of gyration (Rg) and distance D more for 30 bp arrays than for 25 bp arrays. Moreover, face-to-face interactions of 30 bp arrays were substantially decreased along with dihedral angle para, with little change in these parameters for 25 bp arrays. Essentially, the helical fiber formed by wild type 30 bp chromatin is abolished by H4 tail deletion, while the structure of 25 bp chromatin is only modestly changed. These results are consistent with previous reports of 10N array decompaction upon histone H4 tail deletion(93, 101, 102) (93, 101, 102), and with our computational predictions that the H4 tail is more important to 30 bp chromatin structure than to 25 bp chromatin structure.

## 25 bp and 30 bp chromatin form distinct molecular structures in the condensates

**Figure 3.**
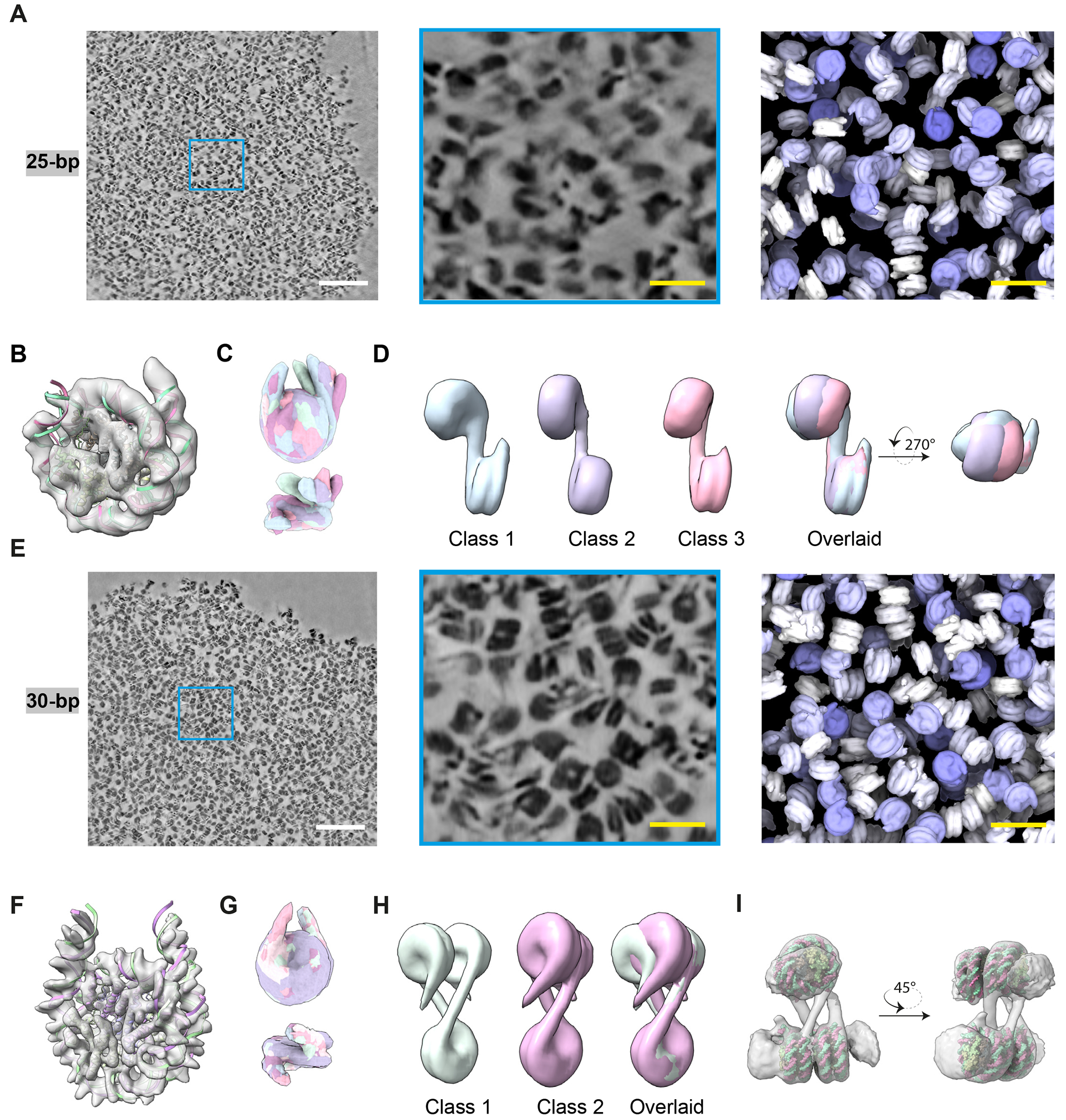
Molecular structures of 25 bp and 30 bp chromatin in the condensed phase. (A) Cross-section of a chromatin condensate in denoised cryo-ET tomogram (left, scale bar 100 nm; middle, magnified view with scale bar 20 nm). Right panel shows nucleosome model assignments from densities in the center panel (scale bar 20 nm). (B) Nucleosome structure (8.0 Å resolution) from 25 bp chromatin obtained by sub-tomogram averaging 104,438 particles in 10 tomograms. Density shown as grey surface, fitted model (PDB: 6pwe) shown as red and green ribbons. (C,D) Classification of mono- and di-nucleosome structures in 25 bp chromatin. Classes colored differently. (E) Overview of 30 bp chromatin condensate, analogous to panel A. (F) Nucleosome structure (5.8 Å resolution) from 30 bp chromatin obtained by sub-tomogram averaging 111, 909 particles in 10 tomograms. Density shown as grey surface, fitted model (PDB: 6L4A) shown as red and green ribbons. (G) Classification of mono- and tri-nucleosome structures in 30 bp chromatin. Classes colored differently and are highly overlapping. (H) Two classes of tri-nucleosome structures in 30 bp chromatin. (I) Structural modeling of a tetra-nucleosome in 30 bp chromatin. Density shown as grey surface, fitted four individual mononucleosome models (PDB: 6pwe) shown as red and green spheres.

We next examined the structure of chromatin in the condensed phase. Using context-aware template matching (CATM) algorithms (103), we placed and oriented the nucleosomes for both 25 bp and 30 bp chromatin condensates (Fig. 3A, E, Movie S3–5). Nearly random orientation distributions with respect to the imaging axis in both cases indicate that sample integrity was preserved and nucleosomes were properly assigned (Fig. S5A, (103)). Subtomogram averaging resulted in nucleosome structures with 8 Å and 6 Å resolution for 25 bp and 30 bp chromatin, respectively (Fig. S5B–D, Table S4), further supporting the accuracy of nucleosome positions and orientations.

During refinement nucleosomes in 25 bp chromatin showed only one linker DNA segment emerging from the core particle (Fig. 3B). Subclassification yielded a panel of structures with distinct orientations of the second linker when aligned based on the first (Fig. 3C). These orientations differed in DNA crossing angle at the nucleosome dyad (Fig. 3C, top) and in deviations out of the nucleosome plane (Fig. 3C, bottom), similar to mononucleosomes analyzed in high salt (104). In further reconstructions of 25 bp dinucleosomes, adjacent nucleosomes adopted different conformations, with varying twist angles (α) between them (Fig. 3D). This flexibility prevented subtomogram averaging of trinucleosomes or more complex structures. The flexibility of both individual nucleosomes and nucleosome pairs is likely related to the reported importance of nucleosome breathing and DNA twisting for efficient chromatin phase separation (57, 88).

The 30 bp chromatin behaves differently (Fig. 3E). Mononucleosomes in 30 bp chromatin condensates could be refined to high resolution with two linker DNA segments clearly observable (Fig. 3F, G). These segments have a crossing angle of 57°, similar to that observed in cryoEM reconstructions of mononucleosomes (Fig. 3F, G, (105, 106)). We could also reconstruct trinucleosome (two conformers, Fig. 3H) and tetranucleosome structures (but not larger), the latter with additional partial nucleosome densities surrounding it (Fig. 3I). Both assemblies show pairs of stacked, alternating nucleosomes (N:N+2), oriented approximately orthogonally to the other nucleosome(s). Our data suggest that the tetranucleosome is the largest well-ordered element in the 30 bp dodecameric arrays within condensates.

Overall, within the condensates 25 bp chromatin adopts more diverse configurations, while 30 bp chromatin is more constrained and stereotypical. This is true for individual nucleosomes and for larger groupings within a fiber. As in the dilute phase, the 30 bp chromatin makes numerous intramolecular face-to-face stacking interactions, which are not frequently observed in the 25 bp chromatin.

## Balance of intra- and inter-molecular interactions determines condensate stability

**Figure 4.**
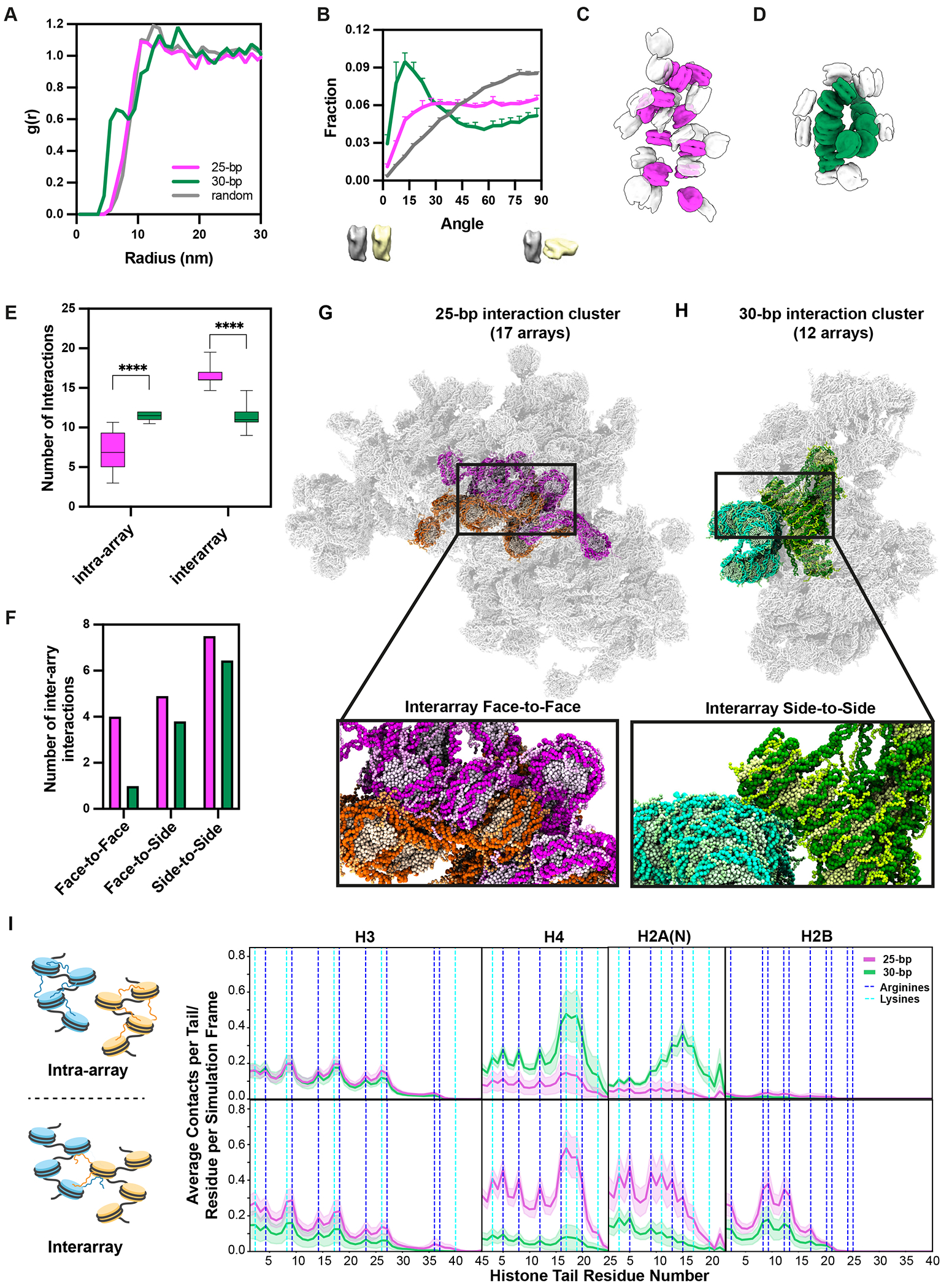
Linker length determines balance of intra- and inter-molecular interactions in the condensed phase. (A) Radial distribution functions, g(r), of 25 bp (magenta), 30 bp (green) chromatin, and a bath of randomly oriented nucleosomes at equivalent density (grey). Peaks at 6, ~12, and ~16 nm in 30 bp chromatin reflect pair-wise and higher-order nucleosome stacking. (B) Distributions of nearest di-nucleosome orientations for 25 bp (magenta) and 30 bp (green) chromatin. Zero and 90 degrees represent parallel and perpendicular orientations, respectively. (C, D) Representative manually traced individual nucleosome arrays (25 bp magenta in C, 30 bp green in D) and their immediate neighboring nucleosomes (grey) within chromatin condensates. See Figures S5E–J and Movies S4, S5 for nucleosome tracing. (E) Intra and inter-nucleosome interactions of 25 bp (magenta) and 30 bp (green) nucleosome arrays within chromatin condensates, Statistical analysis was performed using a t-test; **** indicates p < 0.0001, n = 11. (F) Intermolecular contacts between traced arrays and their immediate neighboring nucleosomes with face-to-face, face-to-side and side-to-side geometries (25 bp magenta, 30 bp green). (G, H) Representative high-resolution snapshots of reconstructed clusters of chromatin arrays in the 25 bp (G, magenta/orange) and 30 bp (H, green/cyan) chromatin condensates. In each panel the array most similar to a traced array in a cryo-ET tomogram (see Methods) is highlighted. All arrays interacting with this central array are shown, with one colored orange (25 bp) or cyan (30 bp). Inset expands image of the two interacting arrays, to show face–to-face (G) or side-to-side (H) inter-array stacking. (I) Number of inter-nucleosome contacts (average per residue per simulation frame) between histone tails of one nucleosome and the DNA and histones of a neighboring nucleosome (intra-array top, inter-array bottom) within simulated clusters (25 bp magenta, 30 bp green, averaged over 400 simulation frames, Table S1.) The blue and cyan vertical lines show positions of lysines and arginines, respectively. Shading indicates one standard deviation from the mean.

To translate molecular features of 25 bp and 30 bp chromatin to phase separation behaviors of the two molecules, we next compared the organization of nucleosomes and nucleosome arrays on larger scales. The radial distribution function, g(r), of nucleosomes in 25 bp chromatin is broad and only modestly different from a random distribution, while that of 30 bp chromatin shows distinct peaks indicating face-to-face stacking (Fig. 4A). Similarly, the distribution of angles between the planes of nearest neighbor nucleosomes in 25 bp chromatin deviates only modestly from a random (sinusoidal) distribution (103) (Fig. 4B). In contrast, nucleosomes in 30 bp condensates show a strong preference for low angles (~parallel geometry), with a maximum at ~15 degrees and substantial depletion for angles >~45 degrees (Fig. 4B). Together, the data are consistent with an abundance of face-to-face stacking in 30 bp condensates and more heterogeneous orientations in 25 bp condensates.

To understand how pairwise nucleosome packing generates interactions between arrays we next sought to connect the assigned nucleosomes into molecular units (Fig. S5E–J, Movies S4, 5). Ambiguities in nucleosome positions/orientations and weak linker DNA density made it slow and laborious to connect nucleosomes into arrays. Nevertheless, we did connect 10 groups of >8 nucleosomes into molecular units in the 25 bp and 30 bp condensates. For each molecule we also identified all surrounding nucleosomes (Fig. 4C, D).

As illustrated in Figures 4C and S6A, B, within condensates 25 bp nucleosome arrays are relatively expanded, with an average R_g_ of 18.2 ±1.3 nm. The molecules adopt numerous, irregular conformations. In contrast, the 30 bp arrays are compact, with average R_g_ of 13.9 ± 0.9 nm, and composed mostly of groups of 4-6 nucleosomes organized into two-start helices, with nucleosomes N and N+2 stacked face-to-face (Fig. 4D, S6C, D). For both chromatin types, the structures observed within the condensates have similar values and distributions of R_g_, α, D and para to those observed in the dilute phase under the same solution conditions (Fig. S6B, E–G), suggesting that phase separation may not strongly impact the conformation and flexibility of individual molecules. One caveat is that molecules recently dissociated from the condensate and captured by freezing proximal to the liquid-liquid interface may not have relaxed to their equilibrium dilute-phase configurations.

The differences in molecular conformation produce starkly different patterns of intra- and intermolecular interactions for the two chromatin types (Fig. 4C, D). Due to their extension and nucleosome arrangements, 25 bp arrays show few intramolecular contacts and many intermolecular contacts (Fig. 4C). The behavior is inverted for 30 bp arrays, which make ~2-fold more intramolecular contacts and ~70% as many intermolecular contacts than 25 bp arrays. The types of intermolecular interactions made by the two systems are also different (Fig. 4E). The 25 bp arrays make a much larger fraction of the most energetically favorable contacts (face-to-face) than the 30 bp arrays, which show a larger fraction of weaker contacts (face-to-side and side-to-side) (83, 87) (Fig. 4F). Thus, within condensates 25 bp chromatin molecules make a larger number of intermolecular contacts, with more energetically favorable geometries, compared to 30 bp chromatin molecules. These differences suggest a greater enthalpic gain upon condensate formation for 25 bp chromatin.

To examine histone tail contacts within the condensates we simulated clusters of arrays surrounding a molecule similar in conformation to one found in the cryo-ET tomograms (Fig. 4G, H and S7, Table S1), and quantified contacts of the histone tails of the central molecule (Fig. 4I). Paralleling the nucleosome contacts observed in the cryo-ET tomograms (Fig. 4E), the simulations revealed that in 25-bp condensates, histone tails make more inter-array than intra-array contacts (Fig. 4I). In contrast, again in agreement with the cryo-ET data (Fig. 4E), most histone tail interactions in 30-bp condensates are intra-array (Fig. 4I).

In 25-bp condensates, chromatin fibers interact frequently via their H4 and H2A(N) tails (Fig. 4I), consistent with the prevalence of inter-array face-to-face stacking (Fig. 3A, 4F–G). Conversely, within 30-bp condensates, the H4 and H2A(N) tails make many fewer inter-array contacts (Fig. 4I). This results in a relatively flat distribution of inter-array contacts across the four tails for the 30-bp chromatin. Notably, the pattern of inter-array contacts in 25 bp chromatin resembles that of intra-array contacts in 30 bp chromatin (dominant H4 and H2A(N)), since both are enriched in nucleosome face-to-face stacking. However, H2B interactions are numerous in the former, but nearly absent in the latter. This difference arises because antiparallel stacking in 25 bp chromatin positions the H2B tail in contact with the entry and exit DNA of the neighboring nucleosome, whereas parallel stacking in 30 bp chromatin positions it more peripherally. Thus, analogous to single fibers (Fig. 2), intermolecular contacts of histone tails in condensates are determined by a combination of fiber conformation and tail position within the nucleosome. As described in Tables S5–6, the patterns of tail interactions observed here are generally consistent with previous biochemical studies of tail contributions to chromatin oligomerization, with the caveat that for 30 bp chromatin, perturbations disrupting nucleosome stacking will affect both oligomerization and the conformation of individual arrays.

Histone tail acetylation is associated with increasing chromatin accessibility in cells and can dissolve chromatin condensates in vitro (33). To investigate how acetylation influences chromatin packing, we treated 30 bp chromatin with p300 histone acetyltransferase and captured temporal snapshots using cryo-ET. Unmodified 30 bp chromatin condensates have virtually no pores larger than 14 nm (Fig. S8A,D). Upon acetylation, larger pores emerge (Fig. S8B–D) and overall nucleosome density decreases (Fig. S8E). Pore number and size increase with the duration of acetylation, with some large enough to accommodate macromolecular complexes such as RNA polymerase II – 15 nm versus pores as large as 20-25 nm. The changes in density and pore size distribution coincide with loss of face-to-face nucleosome packing interactions, indicating disruption of the helical structure of individual arrays (Fig. S8F, G). These observations indicate that acetylation can disrupt local chromatin fiber structure and may induce the formation of physical voids within cellular chromatin, providing a structural mechanism that could facilitate transcriptional activation by enhancing accessibility.

These data lead to a model in which linker length determines the conformation of chromatin molecules, which in turn determines the balance between intra- and intermolecular contacts as well as the energetic strength of those contacts. These contacts then define the thermodynamic stability of their condensates. Twenty-five bp chromatin adopts conformations favoring strong and numerous intermolecular interactions, producing more stable condensates. Thirty bp chromatin adopts conformations that allow weaker and fewer intermolecular interactions (but more and stronger intramolecular interactions), producing less stable condensates (Movie S6).

## Linker length determines connectivity of the condensate network

**Fig. 5.**
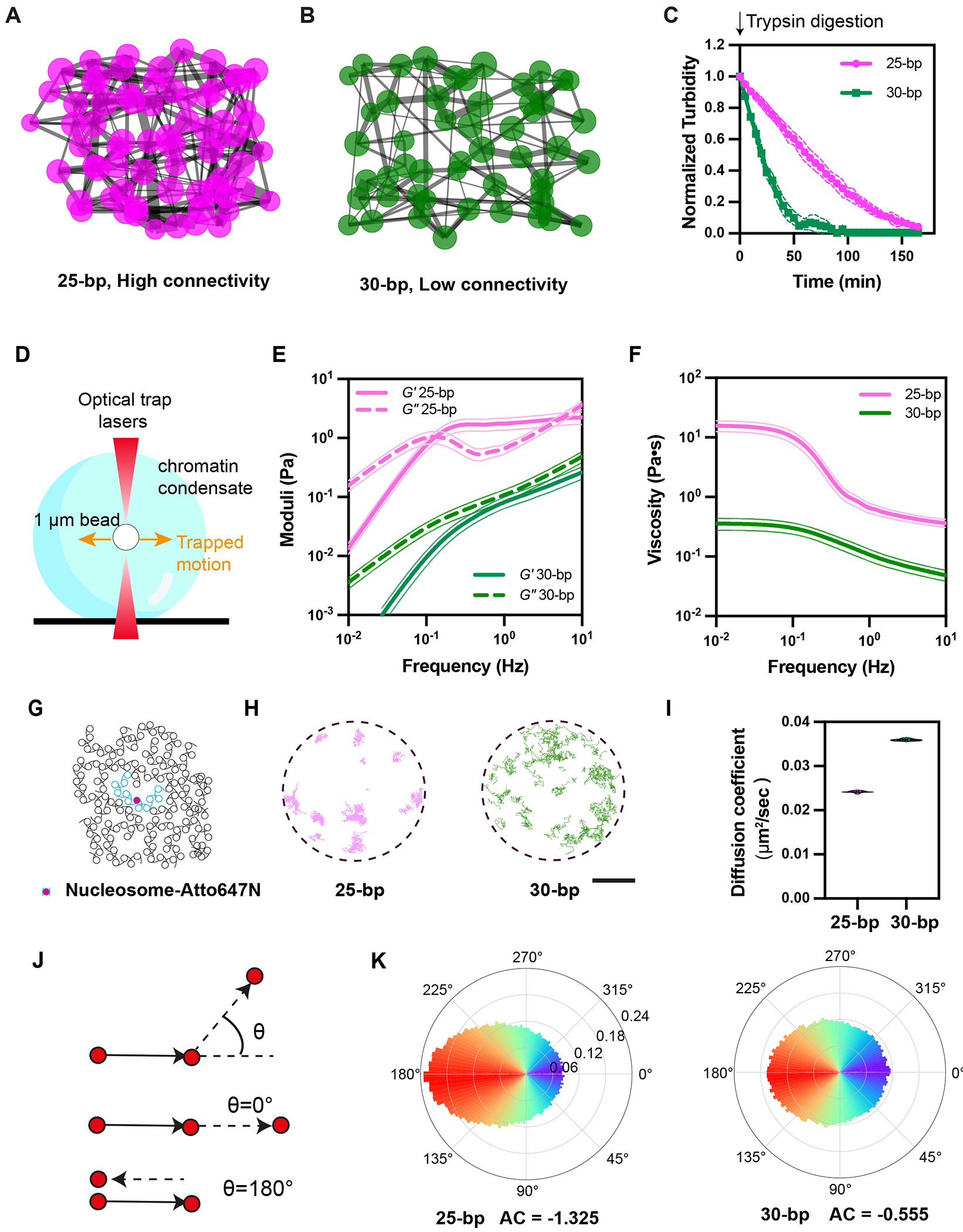
Distinct dynamics of 25 bp and 30 bp chromatin condensate on molecular- and meso-scales. (A-B) Cross sections of interaction networks derived from coarse-grained simulations of 25 bp (A) and 30 bp (B) chromatin condensates. Node diameter is scaled by number of molecules contacted by each array; edge thickness scaled by energy of association between each pair of molecules. (C) Normalized turbidity (A_345 nm_) of solutions containing chromatin droplets after introduction of trypsin. (D) Schematic illustrating Passive Microrheology with Optical Traps (PMOT). 1 μm bead is optically trapped within chromatin condensate, and its trapped motion tracked over time using brightfield camera or quadrant photodetector. (E) Average elastic/storage (G′) and viscous/loss (G″) moduli of 25 bp (magenta; n=12) and 30 bp (green; n=11) chromatin condensates. Representative individual plots shown in Fig. S11. Thick lines indicate average, thin lines represent standard error from the mean. (F) Viscosity of 25 bp (magenta; n=12) and 30 bp (green; n=11) chromatin condensates, calculated from G” data in panel (E); plotted as in (E). Thick lines: average; thin lines: standard error. (G) Schematic illustrating single-molecule tracking experiments. Sparse labelling of nucleosome arrays with Atto647N dye enables tracking of individual molecules. (H) Trajectories of single nucleosome array in 25 bp (magenta) and 30 bp (green) chromatin condensates. Scale bar = 1 μm. (I) Diffusion coefficients calculated for nucleosome arrays in 25 bp (total 7670 trajectories) and 30 bp (total 3167 trajectories) chromatin condensates. (J) Schematic illustration of trajectory angular analysis, where the angle between every pair of steps in a single-molecule trajectory is measured. Zero degrees corresponds to directed motion and 180 degrees represents back-and-forth movement. (K) Trajectory angular analysis distributions for nucleosome arrays in 25 bp (left) and 30 bp (right) chromatin condensates. Asymmetry coefficient (AC) describes the ratio of forward to backward movement on a log_2_ scale.

We next used the minimal model coarse-grained simulations (see Methods) to relate the network of interactions between chromatin arrays to material properties of the condensates. We abstracted the simulations to graph networks where nodes and edges represent array molecules and interactions between them, respectively (Fig. 5A, B, S9A, B). Figures 5A, B show cross-sections through the centers of representative networks generated by temporal snapshots of simulations of each chromatin type. The 25 bp network is more densely connected than the 30 bp network, as described quantitatively by the average number (Fig. S9C) and strength (Fig. S9D, E) of pairwise inter-array interactions, and normalized algebraic connectivity values of 0.88 ± 0.03 and 0.73 ± 0.05, respectively (on a scale of 0 – 1, where 0 indicates no connections and 1 indicates all nodes connected with the highest node degree in the 25 bp chromatin).

To examine network connectivity biochemically we treated the condensates with trypsin, which cleaves histone tails leading to dissolution ((33), (Fig. 5C)). Twenty five bp condensates were more resistant than 30 bp condensates, consistent with greater histone tail-mediated connectivity. We also modeled this process by reducing the number of potential interactions between nucleosomes in simulations. We found that the size of the largest connected cluster of molecules decreased much faster for 30 bp chromatin than for 25 bp chromatin, aligning with the experimental results (Fig. S9F).

Thus, the differences in molecular interactions of 25 bp and 30 bp chromatin produce higher order networks with different degrees of connectivity (Movie S6).

## 25 bp and 30 bp chromatin show distinct dynamics on meso- and molecular scales

The differences in affinity (likely reflecting differences in lifetime) and density of bonds in 25 bp and 30 bp chromatin condensates suggested that the material properties of the two liquids might differ (5, 30, 107, 108). To test this idea on the mesoscale, we measured viscoelasticity of the condensates through passive microrheology using optical traps (pMOT, Fig. 5D). The optically trapped beads move less in 25 bp condensates than in 30 bp condensates (Fig. S10A–D). Analysis of the trajectories yields the viscoelastic modulus (G*), composed of viscous (G”) and elastic (G’) moduli (Fig. 5E, Fig. S10E–N). The 25 bp chromatin condensates have higher G’ and G” than 30 bp condensates in all measured frequency regimes. Moreover, while viscous behavior is dominant (G” > G’) on all timescales for 30 bp condensates, the viscous and elastic moduli intersect at ~0.1 Hz and again at ~5 Hz for 25 bp condensates. Between these frequencies, elastic behavior is dominant (G′ > G″). pMOT measurements using a fast detector revealed a shallow crossover between G’ and G” for the 30 bp condensates only at very high frequencies, ~10^3^ Hz (Fig. S10K, L). Viscoelastic moduli determined by analyses of Brownian motion of fluorescent beads in the condensates showed analogous behaviors (Fig. S11, Movie S7, Table S7). The pMOT data yielded zero-shear viscosity for 25 bp condensates ~40-fold higher than that of 30 bp condensates (Fig. 5F). In cryo-ET tomograms, the 25 bp and 30 bp condensates have comparable nucleosome densities, suggesting that the different material properties likely arise from differences in molecular interaction networks rather than nucleosome density (Fig. S12A).

We used two approaches to computationally examine the viscoelastic behavior of the chromatin condensates. First, we analyzed the trajectories of the coarse-grained simulations to extract the shear stress relaxation modulus, (G(t)) (30), which reveals not only the differential material properties of 25 bp versus 30 bp condensates (Fig. S12B), but also how these changes are governed by distinct chromatin relaxation mechanisms. At short time scales (high frequencies), G(t) is nearly identical for both 25 bp and 30 bp systems. This similarity stems from the short-time behavior being primarily dictated by fast local processes, including intramolecular bond and angle relaxations. In contrast, at long time scales, the 25 bp system consistently exhibits a higher stress relaxation modulus than the 30 bp system. This difference arises because long-time behavior is dominated by slower, collective mechanisms—such as the formation and rupture of nucleosome–nucleosome interactions, large-scale conformational rearrangements of molecules, and diffusion of entire chromatin arrays. These relaxation processes are significantly hindered in the 25 bp condensates due to the stronger chromatin–chromatin interactions, resulting in a more elastic mechanical response at long times.

We next used a recently described formalism to model G’ and G” based on the interaction network observed in the simulations (5). We found that the differences in connectivity between the 25 bp and 30 bp condensates were insufficient to account for their different material properties (Fig. S12C–E). However, differences in interaction strength (likely reflecting longer-lived interactions, accounted for through the polymer friction coefficient in the model) did produce the qualitative differences observed in the data (Fig. S12F). Thus, within the limitations of the formalism (Fig. S10 legend), the modeling suggests that the different intermolecular affinities dominantly account for the differences in viscoelasticity between the two chromatin condensate types.

We also examined dynamics of the condensates on molecular scales by imaging individual dye-labeled array molecules (Fig. 5G, Movie S8). Single 25 bp arrays exhibited slower dynamics than 30 bp arrays, with diffusion coefficients of 0.024±0.0002 μm^2^/sec and 0.036 ±0.0002 μm^2^/sec, respectively (Fig. 5H, I). Additionally, the distribution of jump angles between two consecutive translocations in the trajectories (Fig. 5J) was substantially biased toward 180° for 25 bp arrays compared to 30 bp arrays, indicating less random and more confined dynamics (Fig. 5K). Notably, the calculated asymmetry coefficient (AC), which describes the ratio of forward to backward movement on a log_2_ scale, of 25 bp arrays is similar to that previously reported for single nucleosome dynamics in live cells (−1.33 versus −1.36 (109)). Thus, chromatin motions are more constrained in 25 bp condensates than in 30 bp condensates.

Together, the rheological and single-molecule data suggest that differences in network interactions arising from differences in linker-dependent molecular structure produce quantitative and qualitative differences in the dynamics of chromatin condensates spanning meso- to molecular scales (Movie S6).

## Native chromatin forms domains with nucleosome organization similar to 25 bp condensates

**Fig. 6.**
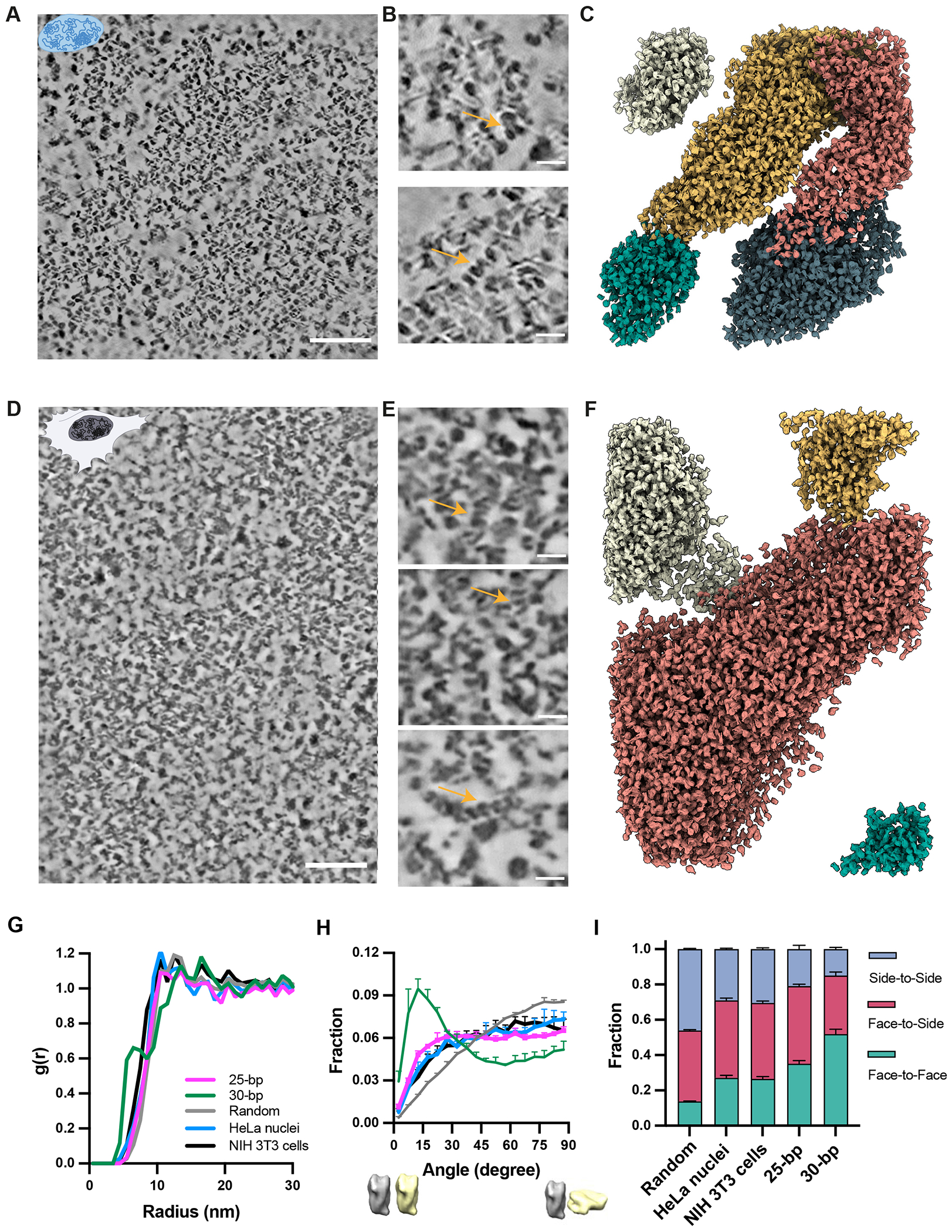
Native chromatin forms discrete domains organized similarly to 25 bp chromatin condensates. (A-F) Tomographic slices and 3D reconstructions illustrating nuclear nucleosome organization. (A-C) Overview slice (scale bar = 100 nm), expanded regions (scale bar = 20 nm) and 3D nucleosome model assignments from a purified Hela cell nucleus. (D-F) Analogous images from the nucleus of an intact NIH3T3 cell. Note that panels B and E are not expansions of regions from A and D, but are derived from different planes of the tomograms. Different colors in panel C, F represent distinct chromatin nanodomains by manual segmentation. (G) Radial distribution functions, g(r), for randomly oriented nucleosomes (grey), nucleosomes in 25 bp chromatin condensates (magenta), purified Hela cell nuclei (cyan), and nuclei of intact NIH3T3 cells (black). (H) Distributions of nearest di-nucleosome orientations for 25 bp chromatin condensates (magenta), purified Hela cell nuclei (cyan), and nuclei of intact NIH3T3 cells (black). Zero and 90 degrees represent parallel and perpendicular orientations, respectively. (I) Fractions of face-to-face, face-to-side and side-to-side pairwise nucleosome contacts in a random nucleosome distribution, 25 bp reconstituted chromatin condensates, nucleosomes in purified Hela cell nuclei, and nuclei of intact NIH3T3 cells.

We investigated chromatin organization in vitrified HeLa cell nuclei and intact NIH 3T3 cells using cryo-FIB milling and cryo-ET (Fig. 6A–F). Each system had nuclear domains of high nucleosome density, varying in size from ~100-300 nm, surrounded by regions of lower density. These structures, which were also reported in a recent cryo-ET study of T-lymphoblast CEM RPE-1 nuclei (65, 70), are similar in size to domains of high-density DNA observed by super-resolution fluorescence microscopy (49, 50). Staining for epigenetic marks suggested that those structures corresponded to inactive chromatin (50). Thus, the high-density domains we observe here may be inactive chromatin as well.

Radial distribution functions and overall density of nucleosomes in each native chromatin sample were similar to those of synthetic 25 bp chromatin condensates (Fig. 6G, S13A). The orientation distribution and patterns of packing geometries of nearest nucleosome pairs were also similar for native chromatin and 25 bp chromatin condensates, showing modest deviations from a random distribution (Fig. 6H, I, S14). Thus, at the level of individual nucleosomes, dense chromatin regions and 25 bp chromatin condensates have packing arrangements that deviate similarly from randomly organized molecules, likely reflecting geometric features of nucleosome interaction energies and restrictions imparted by DNA linkers.

Inspection of the dense chromatin regions revealed some groups of nucleosomes stacked into two-start helices analogous to those in synthetic 30 bp chromatin condensates (but shorter, Fig. 6B, E, Fig. S13B–H). These may be formed by genomic regions with nucleosomes having ~10N spacing. These structures might be functionally important if recognized by specific factors or associated with specific loci. Overall, our data show that native chromatin from these cellular sources forms ~100-300 nm scale dense domains composed mostly of nucleosomes that are packed non-randomly and have an excess of contacts involving nucleosome faces, with occasional instances of two-start helical fibers.

## Discussion

Our studies of chromatin illustrate the types of information that may be gained from future cryo-ET studies of other biomolecular condensates composed of large, electron dense units. Comparison of dilute phase structures above and below the phase separation threshold can reveal conformational changes that drive assembly. Sub-tomogram averaging can afford medium resolution structures of individual particles in the dense and dilute phases. For multivalent systems it may be possible to reconstruct higher order units of the core particle (e.g. multi-nucleosome groupings, Fig. 3). It may also be possible to observe and reconstruct client molecules that bind the phase separating scaffold molecules. At a higher level, one can visualize the spatial arrangement of components on nano- to micrometer length scales. Such analyses will reveal local packing interactions (distances, geometries, pore sizes; energetics, when coupled with modeling), which inform on the drive to phase separate. On longer scales, network architecture, when coupled with dynamics measurements, will inform on viscoelasticity. Comparison of molecular organization in the core and surface of a condensate can provide a structural explanation for surface tension (103) and potentially surface chemistry. Future developments in the representation and analyses of molecules within condensates should yield additional information and insights beyond those considered here.

These features are relevant to diverse aspects of condensate function. Structures of individual particles within a condensate may provide insight into activity changes that accompany phase separation. Pore sizes in the molecular network will be relevant to recruitment, movement and chemistry of other molecules within a condensate (110–113). Viscoelasticity will dictate the length- and timescales at which internal processes will be impacted by the condensate solution environment, and how the condensate will respond to mechanical forces (114–116). Structures will provide a framework to understand the physical properties of the compartment such as pH, ion concentrations (15, 117) and hydrophobicity (14, 118). Finally, condensate structural analyses should explain how disease-causing mutations alter both molecular and higher order organization to produce functional defects (119). These types of analyses will be most applicable to biochemically reconstituted systems where all components are known, but nevertheless can be applied also in cells when certain components are large and stereotypical.

A long-standing question in the chromatin field is how the 1-dimensional arrangement of nucleosomes along a DNA strand dictates the higher-order organization of the strands to produce a 3-dimensional architecture. Numerous studies have shown that 10N base pair nucleosome spacing produces more compact fibers (85, 86, 120), while 10N+5 spacing facilitates higher order assembly (33, 83). Our cryo-ET data and simulations explain these findings in structural detail. They reveal how differences in nucleosome spacing produce distinct fiber conformations with different histone tail interactions. These conformational differences then determine the balance between intra- and intermolecular contacts, dictating the drive for higher order assembly as well as the material properties of the condensed states. As a step toward connecting this structural information to function, we also show that weaking histone tail interactions through acetylation alters the pore size distribution within condensed chromatin, which is likely important to acetylation-dependent processes such as transcription and DNA repair. Future studies examining 1-D to 3-D relationships in chromatin containing other regulatory post-translational modifications could yield general principles regarding how different compartments of chromatin are formed and function.

Cryo-ET images here, and elsewhere (65, 70), show that nuclear chromatin forms dense ~100-300 nanometer foci of concentrated nucleosomes, surrounded by regions of appreciably lower density. These foci and 25 bp chromatin condensates have similar nucleosome density and packing arrangements. It is possible that the foci form through micro-phase separation (i.e. phase separation of a small element of one or more chromosomes (121)), although other mechanisms are also possible. It remains unclear how these foci relate to genome organization characterized by single-molecule imaging and interaction mapping technologies. The former have shown that small groups of 10-50 nucleosomes, termed clutches, assemble into discrete units (36). The latter have suggested that on a cell population basis genomic regions of 0.2 – 1 Mbp associate into topologically associated domains (TADs) (36, 122, 123). Such large domains are not typically observed in interaction maps of individual cells sampled at single time points, where only subsets appear to associate (124–127). But the larger TADs may reflect cellular genome organization averaged over time. The foci we observe by cryo-ET have density of approximately 600,000 nucleosomes/μm^3^. Assuming an average nucleosome repeat length of 192 base pairs (128), this density corresponds to 115 Mb base pairs/μm^3^. As a rough estimate, foci of 100-300 nm diameter would contain 315 – 8500 nucleosomes and 60 kbp – 1.6 Mbp of DNA. Thus, the foci are much larger than clutches and more consistent in size with TADs. It remains unknown how these foci, each of which represents only a tiny fraction of the human genome (< 0.5 %), relate to nuclear functions such as transcription, replication, etc. Future studies based on correlated light- and electron microscopy, to understand epigenetic marks and perhaps genomic regions present in the foci, will be important to understanding how these higher-order chromatin structures form and what their functions may be.

## Supplementary Material

scienceadv6588_movie_s1

scienceadv6588_movie_s2

scienceadv6588_movie_s3

scienceadv6588_movie_s4

scienceadv6588_movie_s5

scienceadv6588_movie_s6

scienceadv6588_movie_s7

scienceadv6588_movie_s8

accepted version supp matls

## Data Availability

All cryoET data are deposited in the Dryad database (129). The subtomogram averages of nucleosome structures from the 25 bp and 30 bp chromatin condensates have been deposited in the EMDB under accession numbers EMD-70745 and EMD-70743, respectively. The CATM software and all cryoET analysis scripts are available on Zenodo (130). All simulation code has also been deposited in Zenodo (131). All reagents described in the work will be available by request to the corresponding authors.
