# Supplementary material for: Multiscale structure of chromatin condensates explains phase separation and material properties
Source: Science. Author manuscript; Available in PMC 2026 Feb 24. (PMC12929043; doi:10.1126/science.adv6588)
Supplement: accepted version supp matls [file NIHMS2143018-supplement-accepted_version_supp_matls.pdf]

Science
AAAS

Supplementary Materials for

Multiscale structure of chromatin condensates explains phase separation and material properties

Huabin Zhou, Jan Huertas, M. Julia Maristany, Kieran Russell, June Ho Hwang, Run-Wen Yao, Nirnay Samanta, Joshua Hutchings, Ramya Billur, Momoko Shiozaki, Xiaowei Zhao, Lynda K. Doolittle, Bryan A. Gibson, Andrea Soranno, Margot Riggi, Jorge R. Espinosa, Zhiheng Yu, Elizabeth Villa, Rosana Collepardo-Guevara*, Michael K. Rosen*

Corresponding author: rc597@cam.ac.uk (R.C.G.), michael.rosen@utsouthwestern.edu (M.K.R.)

The PDF file includes:

Material and Methods
Figs. S1 to S14
Tables S1 to S7
References (132-166)

Other Supplementary Materials for this manuscript include the following:

Movies S1-S8

Materials and Methods

Protein and Nucleosome Arrays, Native Chromatin

Nucleosome arrays (*33*), HeLa nuclei, and NIH3T3 cells (*103*) were prepared as described previously. Xenopus histone octamers were used for the H4 tail deletion experiments (Fig. 2I–K, S4B–D), while human octamers were used for all other experiments.

Chromatin Phase Separation

Chromatin condensates were induced by combining 6 μM of either 25 bp or 30 bp nucleosome arrays with an equal volume of a 2X phase separation buffer (40 mM Tris-OAc pH 7.5, 300 mM KOAc, 10% glycerol, 0.2 mM EGTA). These condensates were subsequently used in a series of experiments, including FRAP, droplet fusion, cryo-ET, trypsin digestion, single-molecule tracking, and microrheology.

Phase Diagram with Turbidity Measurement

The extent of phase separation was assessed by measuring absorbance at 345 nm using a NanoDrop One UV-Vis spectrophotometer (Thermo Scientific). Nucleosome arrays were diluted to the desired concentration using a buffer containing 20 mM Tris-OAc pH 7.5, 0.1 mM EGTA, and mixed with an equal volume of 2X phase separation buffer at varying KOAc concentrations. Each sample was measured three times, and the results were averaged.

FRAP of Chromatin Condensates

FRAP experiments were performed using a Leica SP8 confocal fluorescence microscope equipped with a 561 nm laser for photobleaching. Prior to photobleaching, three frames were captured to establish a baseline. A region of interest (ROI) within the chromatin condensate (<10% of condensate diameter) was selectively bleached with a high-intensity laser for 5 seconds. Fluorescence recovery within the bleached ROI was monitored at 5-second intervals over a total period of 300 seconds.

Images were captured using PMT detectors paired with a 60x oil immersion objective. Fluorescence intensity within the ROI was quantified before and after bleaching using Leica LAS-X software. Recovery curves were plotted to determine the mobile fraction and the half-time of fluorescence recovery. Data analysis was conducted using easyFRAP (*132*); curves were fitted using double-scale normalization.

Imaging and Analyzing Droplet Fusion

Real-time imaging of droplet fusion was conducted using a Leica DMI6000 microscope, equipped with a Yokogawa CSU-X1 spinning disk confocal scanner unit and a Hamamatsu ImagEMX2 EM-CCD camera. A Leica 40x oil immersion objective was employed for imaging. Time-lapse sequences were recorded at 100 ms per frame.

Images were acquired using Metamorph (Biovision) software. The time-lapse images were analyzed with FIJI/ImageJ software to track droplet fusion dynamics. Fusion events were identified manually. To determine the time constant (τ) for each condensate fusion event, the change in aspect ratio (AR) over time was measured, fitting the values to an exponential decay

model: AR = 1 + (ARinit − 1) * exp(−t/τ), where ARinit represents the initial aspect ratio at the onset of fusion.

Trypsin Digestion and Turbidity Measurement

Chromatin condensates (22 µL), prepared as described above, were mixed with 3 µL of trypsin (4 ng/µL in 25 mM Tris-HCl, pH 7.5) in an mPEG-Silane pretreated imaging plate (*35*). The plate was immediately placed in a plate reader (Tecan Spark) to monitor turbidity changes by measuring absorption at 345 nm until the signal reached a plateau. The measurements were conducted in three independent wells. The results were normalized such that the start time was set to 1 and the end time to 0, followed by averaging across replicates. Standard deviations are shown to indicate variability.

Acetylation of Chromatin Condensates

Chromatin acetylation was performed following a previously described protocol (Gibson, 2019). Briefly, nucleosome arrays were assembled on DNA containing a terminal TetO binding site, using 30 bp spacing across a 12×601 DNA sequence. Phase separation was induced by mixing 20 µL of 6 µM nucleosome arrays with 20 µL of Phase Separation Buffer (40 mM Tris-OAc, pH 7.5; 300 mM KOAc; 10% glycerol; 0.2 mM EGTA) and incubating the mixture at room temperature for 10 minutes. The resulting condensates were then combined with 1 µL of 6 µM GFP-TetR-p300-HAT and 1 µL of 10 mM Acetyl-CoA. After 6 minutes of incubation, 3 µL aliquots were taken every 2 minutes for high-pressure freezing.

Cryo-ET Sample Preparation and Data Collection

Chromatin samples in low and medium salt buffer

To prepare chromatin samples under low salt conditions, Lacey carbon grids (300 mesh, Ted Pella Inc) were glow discharged using an easiGlow (Pelco) at 30 mA for 30 seconds. A Vitrobot Mark IV (Thermo Fisher) was pre-cooled to 4°C and set to 100% humidity. For low salt condition, a 3 µL aliquot of nucleosome array solution (6 µM in 20 mM Tris-Cl, 0.1 mM EGTA) was applied to the grid, blotted with a force setting of 0 for 4 seconds, and plunge frozen in liquid ethane. Under medium salt conditions, 5 µL of nucleosome arrays were mixed with 5 µL of buffer (40 mM Tris-OAc, pH 7.5, 120 mM KOAc, 10% glycerol, 0.2 mM EGTA). A 3 µL aliquot of the mixture was then applied to cryoEM grids for plunge freezing.

Chromatin samples in high salt buffer

A 4 µL aliquot of phase separated nucleosome array solution (6 µM total nucleosome concentration in 20 mM Tris-Cl, 150 mM KOAc, 0.1 mM EGTA) was vitrified using the waffle method (*133*) on a Wohlwend Compact 03 high-pressure freezer as previously described (*103*). After freezing, grids were analyzed by cryo-fluorescence imaging (Leica EM Cryo-CLEM system equipped with a 50x/dry objective (NA=0.9)) to identify regions containing condensates, then transferred to an Aquilos II cryoFIB-SEM (Thermo Fisher). Lamellae (60-200 nm thick) were randomly generated in these regions using notch-milling procedures, and condensates were identified using the SEM mode of the instrument, as previously described (*103*, *133*).

Cryo-ET Data Collection

For chromatin samples with a 25 bp linker length in low salt conditions, tilt-series were collected using a 300 kV Titan Krios G1 (Thermo Fisher Scientific) equipped with a high-brightness field emission gun (xFEG), a spherical aberration corrector, a BioQuantum energy filter, and a post-

GIF K3 camera (Gatan Inc.). Tilt series were acquired using a dose symmetric scheme over a tilt range from -60° to +60° with a 3-degree increment. For each tilt angle, a movie was recorded at a nominal magnification of 33,000x, with a calibrated physical pixel size of 0.206 nm in CDS (correlated double sampling) counted mode. A 20 eV energy slit was used and the total electron dose was limited to 162 electrons/Å². A Volta phase plate was employed, with a targeted defocus set to -0.5 µm (Table S4).

For chromatin samples with a 30 bp linker length in low salt conditions, tilt-series were collected using a Titan Krios G3i (Thermo Fisher) equipped with a cold field emission gun, a Selectris X energy filter, and a Falcon 4i camera. Images in each tilt series were acquired using a dose symmetric scheme over a tilt range from -45° to +45° with a 3-degree increment. For each tilt angle, a movie was recorded at a nominal magnification of 53,000x with a calibrated pixel size of 0.23 nm. A 5eV energy slit was used and the total electron dose was limited to 120 electrons/Å² (table S4).

For chromatin condensates in high salt conditions, data collection was performed using the Krios G3i as described above. The tilt series spanned angles from -48° to +60° with 2° increments per tilt. Images were recorded at a physical pixel size of 0.1516 nm at the specimen level. The defocus values ranged from -3 µm to -4.5 µm, and the total electron dose was limited to 178 electrons/Å² (Table S4, (*103*)).

Cryo-ET Data Analysis

The tilt-series data were pre-processed using Warp (*134*) for motion correction and CTF estimation, then subsequently aligned with AreTomo (*135*). The alignments were used in Warp to reconstruct two half-set tomograms with a pixel size of 8 Å and denoised sequentially (*103*) using Warp (*134*) and IsoNet (*136*).

For tomograms of chromatin under low salt conditions, nucleosome particles were picked using crYOLO (*137*). A subset of tomogram slices was manually annotated for training purposes. After particle picking, the tomograms were manually inspected to remove junk particles and particles closer than ~30 nm to the air-water interface.

Tomograms of chromatin condensates were initially segmented using DeepFinder (*138*), and the centers of particles were localized with the MeanShift algorithm. The particles with estimated positions were then processed through our custom algorithm, Context-Aware Template Matching (CATM), to determine nucleosome orientation and refine particle centers (*103*). Briefly, the particles were extracted from the tomogram and subjected to local template matching using a nucleosome structure low-pass filtered at 25 Å. For each particle, multiple positions and orientations, along with their corresponding cross-correlation coefficients, were recorded. This information was then used to optimize particle assignments and eliminate clashes. Once nucleosome orientations and positions were accurately assigned, sub-tomograms were generated in Warp and further refined in Relion (*139*, *140*) to yield the final positions of all nucleosomes.

Tri-Nucleosome Model Building

To build tri-nucleosome models (Fig. S2E), individual nucleosomes were first aligned to reconstruct an average mono-nucleosome. The refinement box size was then expanded to align adjacent nucleosomes, which were identified at either DNA end of the central nucleosome in

separate classes. The di-nucleosome structures, with adjacent nucleosomes at either end, were then aligned based on the central nucleosome to create a tri-nucleosome structure for visualization (note that all geometric analyses were performed using individual nucleosomes, not these averages, which are purely for visualization). Due to the limited number of particles available, we were unable to achieve high-quality sub-tomogram averaging for the 30 bp structure in high-salt dilute phase conditions. Therefore, we used the tri-nucleosome structure of the 30 bp chromatin in the high-salt condensate phase as a representative in Fig. 1H, as the conformations under these two conditions are very similar (Fig. S6B, E-G).

Tracing of Individual Nucleosome Arrays

The tracing process begins by positioning the assigned nucleosomes into the tomograms using ChimeraX (*141*) with the Artix plugin (*142*) for visual inspection. Erroneous assignments were manually removed, and the in-plane rotation of nucleosomes was adjusted based on their shape and linker DNA density. Connectivity between nucleosomes within the array was manually assigned. The traced nucleosome positions and orientations were further refined in CATM (*103*) to locally optimize their orientations and resolve particle clashes.

The traced nucleosome arrays were then used to quantify nucleosome conformations. The distance between nucleosome N and N+2 was calculated using Euclidean distance (D) between their centers of mass, and the dihedral angles between adjacent (α) or alternating (para) nucleosomes were determined by calculating the angles between their planes. Dihedral angles were calculated using vectors perpendicular to the nucleosome planes. To calculate α, for the 25 bp nucleosome array, adjacent nucleosome vectors were oriented oppositely, reflecting the 2.5 turns of DNA between them. The angle between these vectors was measured and scaled to a range of 180°–270°, where 180° corresponds to parallel nucleosome planes and 270° to perpendicular planes. For the 30 bp array, the vectors were aligned due to the 3 turns of DNA, and the angle between them was calculated and scaled to 0°–90°, with 0° indicating parallel planes and 90° indicating perpendicular planes. To calculate para, the vectors were aligned, and the resulting angle was similarly scaled to 0°–90°.

Classification of Mono, Di, and Tri-Nucleosome Conformations in Chromatin Condensates

To assess mono-nucleosome structural variability, 3D classification was performed using Relion. Subtomograms were sampled at a pixel size of 8 Å with 64x64x64 voxels. Nucleosomes were classified into six classes over ten iterations in Relion using a 200 Å diameter spherical mask. The resulting nucleosome structures were aligned based on one linker DNA to observe structural variability in the other linker DNA.

To analyze 25 bp chromatin structural variability at the di-nucleosome level, nucleosomes were classified into eight classes. This analysis produced 35.8% of nucleosomes with both one adjacent nucleosome and the linker DNA well resolved; these nucleosomes were categorized into three different classes. To analogously explore the conformational diversity of multi-nucleosome groups in 30 bp chromatin, nucleosomes were classified, producing 56.5% with well-resolved linker DNA and two adjacent nucleosomes. These well-resolved particles were further organized into two different nucleosome stacking patterns: 20.6% of particles exhibited an offset between stacked nucleosomes, while 79.4% of particles displayed perfect face-to-face stacking (Fig. 3H).

Nucleosome Pair Orientations

In our coordinate system, the origin is set at the centroid of the reference nucleosome, which is approximated as having 2-fold symmetry about the dyad axis (i.e., composed of palindromic DNA). The Z-axis is defined as the direction along which the nucleosome projection is maximum (i.e. perpendicular to the nucleosome plane). The X-axis extends from the centroid to the nucleosome dyad, and the Y-axis is orthogonal to the X-Z plane. For each nucleosome identified through template matching, we calculated the angle between its Z-axis and either the beam direction or the two directions perpendicular to the beam. A random arrangement of nucleosomes will exhibit a sinusoidal distribution of each angle (*143*).

The relative orientation of two nucleosomes was classified into three categories: face-to-face, side-to-side, and face-to-side (Fig. 4F and 6I), similar to definitions described by Farr and colleagues (*88*). Classification was based on vectors perpendicular to the nucleosome planes (i.e. along the Z-axes) through their centroids according to the following algorithm:

For nucleosomes i and j, the angles θ and ψ are defined according to:

$\hat{\mathbf{z}}_i \cdot \hat{\mathbf{z}}_j = \cos\theta$

$\hat{\mathbf{z}}_i \cdot \hat{\mathbf{r}} = \cos\psi_i$

$\hat{\mathbf{z}}_j \cdot \hat{\mathbf{r}} = \cos\psi_j$

where $\hat{\mathbf{z}}_i$ and $\hat{\mathbf{z}}_j$ are the unit vectors of nucleosomes i and j, and $\hat{\mathbf{r}}$ is the unit vector pointing from the centroid of nucleosome i to the centroid of nucleosome j. Thus, θ denotes the angle between the two unit nucleosome vectors (used in Figures 4B and 6H), and ψ denotes the angle between a nucleosome unit vector and the line between the nucleosome centroids.

```
if θ < 45° or θ > 135°:                                  {nucleosomes ~parallel)
if ψi < 45° or ψi < 135° or ψj < 45° or ψj > 135°:       {one nucleosome above the other}
   face-to-face
else:                                                    {nucleosomes beside each other}
      side-to-side
else:                                                    {nucleosomes ~perpendicular)
   if ψi > 45° and ψi < 135° and ψj > 45° and ψj < 135°: {nucleosome edges beside each other}
      side-to-side
   else:                                                 {perpendicular packing}
       face-to-side
```

Only nucleosome pairs whose centroids were closer than 13 nm were considered in the analysis of relative orientation.

Radial Distribution Analysis

To analyze the radial distribution of nucleosomes within the condensates, a spherical region with 60 nm diameter centered on each nucleosome was used to estimate the local nucleosome concentration. To avoid edge artifacts, the 500 nucleosomes with the highest local concentrations were used in the analysis to ensure they were fully within the condensates. For each nucleosome, a series of concentric shells of 1 nm thickness with outer diameters incrementing by 1 nm up to 60 nm was generated. The number of particles within each spherical shell was calculated and normalized against the average nucleosome concentration and the volume of the shell. The distribution was then averaged across all nucleosomes.

Radius of Gyration (Rg) Calculation

To calculate the radius of gyration for nucleosome arrays, we first identified the center of mass by averaging the coordinates of each nucleosome position. Next, we computed the squared distances of each nucleosome from this center of mass. The radius of gyration was obtained as the square root of the mean of these squared distances. Only arrays with more than 4 nucleosomes were considered, and Rg was normalized by scaling it with the square root of the ratio between the reference count (12) and the actual number of nucleosomes. Statistical comparisons were conducted using the Kruskal–Wallis test ($n \geq 10$). Asterisks indicate significance levels: *$p < 0.05$, **$p < 0.01$, ***$p < 0.001$, and ****$p < 0.0001$.

Calculation of Nucleosome Concentration in the Condensate

To minimize boundary effects, we manually defined a 3D box within each tomogram by visually inspecting and setting the minimum and maximum values along the x, y, and z axes. Within this defined volume, we quantified the number of nucleosomes and converted the count into molar concentration based on the box dimensions.

Measuring Pore Sizes in Condensates

To measure the pore sizes in the condensates, we first trained the EMAN2 segmentation algorithm on our tomograms. For each tomogram, a subvolume of interest was extracted and processed to detect cavities. We characterized cavities by fitting a collection of the largest possible non-overlapping spheres inside them. We then plotted the distribution of sphere diameters for the entire condensate subvolume, normalized to 100 nm^3 volume to allow comparison between datasets.

Segmentation of Chromatin Domains in Native Chromatin Tomograms

To examine chromatin segregation within tomograms of Hela nuclei and NIH3T3 cell nuclei, we used IMOD (*144*) to manually delineate the segregated domains. The assigned nucleosomes were then mapped back to their respective domains and visualized in ChimeraX (*141*) using the ArtiaX plugin (*142*).

Multiscale Modelling of Chromatin Fibers in the Dilute and Condensed Phases

To represent chromatin fibers, we use our multiscale chromatin model (*88*), a bottom-up approach that combines atomistic information of nucleosomes, DNA, and proteins, with two levels of coarse-graining: chemical-specific and minimal. This model enables us to explore how the chemical composition of chromatin influences its structure and propensity to phase separate. We adopted a multiscale strategy to take advantage of both (1) the precision of atomistic models, which can reveal how chemical modifications alter the local behavior of proteins and DNA, describe protein-chromatin binding, and explain how DNA sequence affects mechanical properties, and (2) the efficiency of coarse-grained models, which significantly reduce system dimensionality—for example, representing a 100 kb chromatin region (approximately 10 million atoms plus solvent) with just ~15K beads. These two coarse-grained models are described below.

<u>Chemical-Specific Coarse-grained Model.</u>

The chemical specific chromatin coarse-grained model explicitly represents each amino acid in the histone proteins as a bead centered on its alpha-carbon. Beads corresponding to lysine, arginine, aspartic acid, glutamic acid, and histidine carry the full charge of their atomistic counterparts at

pH ~7. Each amino acid bead has a relative hydrophobicity and diameter values derived from atomistic simulations and experimental data (*88*). The histone protein core is modeled using an elastic network that maintains the secondary structure of histones in the 1KX5 crystal structure. Histone tails are treated as flexible polymers, with bonds between consecutive residues maintained by a stiff harmonic potential, without penalizing for bending or torsion.

Screened electrostatic interactions between all non-bonded beads are approximated using the Debye-Hückel model. Non-ionic associations are modeled with a Lennard–Jones potential. All model parameters and energy functions are detailed in the previous studies (*88*, *145*).

Minimal Coarse-Grained Chromatin Model.

Our minimal chromatin model (*88*) represents the histone core as a single bead, modeled as an ellipsoid with radii of 28 × 28 × 20 Å. Linker and nucleosomal DNA are represented by finite-size orientable spheres (an ellipsoid of 12 × 12 × 12 Å), with one sphere per 5 base-pair segment. To capture the mechanical properties of DNA, we developed a minimal "Rigid-Base-Pair-like" model with a resolution of 5 base pairs per bead. The minimal helical parameters were optimized based on chemically specific coarse-grained simulations of 200 bp DNA strands (*88*). Nucleosome–nucleosome and nucleosome–DNA interactions are modeled using a set of orientation-dependent potentials, fitted to reproduce the internucleosome potentials of mean force obtained from our chemically specific coarse-grained chromatin model. All model parameters and energy functions are detailed in our pervious study (*88*).

Reconstruction and Simulation of Chromatin Fibers in the Dilute Phase Using the Chemical-Specific Model

Flat bottomed harmonic restraints were used to impose the inter-nucleosomal distances obtained from cryo-EM tracing onto simulated nucleosome arrays using our GPU-accelerated chemical-specific coarse-grained chromatin model. The restraints were implemented in OpenMM using a CustomCentroidBondForce with the following expression:

$$V(r) = \begin{cases} 0.5k\,(r - (r_{eq} + tolerance))^2 \; if\; r > r_{eq} + tolerance, \\ 0.5k\,(r - (r_{eq} - tolerance))^2 \; if\; r < r_{eq} - tolerance, \\ 0 \quad otherwise. \end{cases}$$

with r being the centroid distance between each 2 nucleosomes. A value of 5 kJ/mol/nm^2 was used for k. The value of tolerance used for a given bond incorporated the cross-correlation coefficient (CCC) obtained from the cryo-EM maps, with the formula 1 – $CCC_{average}$ being used to calculate the final tolerance, where $CCC_{average}$ is the average CCC of the two nucleosomes the constraint is applied to. The tolerance was gradually decreased from 2(1 – $CCC_{average}$) to the final value in five sets of 50 ns simulations. All initial steps were performed at a salt concentration of 60 mM, before the simulation was ran for 500 ns at its target salt concentration (100 mM for high salt and 25 mM for low salt). The last 400 ns were retained for analysis. The following number of independent systems were simulated: 12 (25bp low salt), 11 (30bp low salt), 7(25bp high salt), 5(30bp high salt). For the number of nucleosomes on each system see Table S1.

Reconstruction and Simulation of Chromatin Fibers inside Condensates Using the Chemical-Specific Model

In principle we could have used the approach described above for dilute phase fibers (Fig. 2) to analyze condensate fibers surrounded by their neighboring nucleosomes (Fig. 4C, D). However, we were concerned that because the surrounding nucleosomes could not, in most cases, be connected to one another, tail behaviors of the fibers might be in error. To address this concern we developed an alternative, multiscale approach that leverages coarse-grained models at two resolutions (*88*) (Fig. S7A). As described in Figure S7, coarse-grained simulations using the low resolution, minimal model of 25 bp and 30 bp chromatin condensates produced collections of molecular structures whose distributions of Rg values contained those of the molecules that we could trace in the experimental tomograms. Moreover, we recently showed that these simulations quantitatively recapitulate the experimentally determined phase separation thresholds of a series of chromatin molecules (*83*). Thus, here we first used direct coexistence simulations using the minimal model, to generate approximately 10,000 uncorrelated equilibrium configurations of chromatin condensates, each containing 125 interacting 12-nucleosome chromatin fibers at high salt, with either 25 or 30 bp linkers (Fig. S7A iv; note that these same coarse grained simulation trajectories were also reported in (*83*)). Then for each of the fibers that could be traced in the cryo-ET tomograms for each chromatin type, we searched the full simulation ensemble for the best match (among ~1,250,000 individual fibers). Scoring was based on the root mean square deviation (RMSD) between the geometric centers of the nucleosomes in the cryo-ET and simulated fibers (Fig. S7A i-ii, and iii, respectively). The top-scoring (lowest RMSD) fiber was tracked back to its simulated condensate (Fig. S7A iv) and extracted along with its full interaction cluster—i.e., all neighboring fibers that contacted it (Fig. S7A v).

Subsequently, this cluster was backmapped from the minimal coarse-grained model to our higher resolution chemically specific model, enabling its reconstruction at the amino acid and nucleotide level (Fig. S7A vi). To perform this backmapping, we used a shorter, 15 ns version of the protocol for dilute reconstructions with tolerance set to 0, as the coordinates obtained from the minimal model have no associated uncertainty. The reconstructed models were then added one by one to assemble the full interaction cluster, with harmonic restraints applied to constrain the inter-nucleosome distances, maintaining the array conformation. A second CustomCentroidBondForce, with expression:

$$V(r) = k'((x - x_0)^2 + (y - y_0)^2 + (z - z_0)^2)$$

where x.y and z are the coordinates of a nucleosome centroid, and x_0, y_0 and z_0 are the target coordinates of that nucleosome centroid, was then used to pull each newly added array to its correct position, with k' gradually ramping from 0 to 2.5 kJ/mol/nm² over a period of 2.5 ns each time a new array is added. As before, the entire reconstruction procedure was carried out at a salt concentration of 60 mM, before switching to the final salt concentration of 100 mM once all arrays were in place. The final configuration was then simulated for 500 ns with all restraints in place, with the final 400 ns retained for analysis.

Histone-tail Contact Analysis

For the analysis of tail-mediated interactions of chromatin arrays in both the dilute phase (low and high salt) and inside the condensates, we built chemically specific coarse-grained models of the chromatin arrays based on the cryo-ET data and performed molecular dynamics simulations as detailed above. For each experimental condition (25 bp low salt dilute phase, 25 bp high salt dilute phase, 30 bp low salt dilute phase, 30 bp high salt dilute phase, 25 bp high salt condensate, 30 bp

high salt condensate), we ran independent simulations for each of the available experimentally resolved chromatin arrays (Table S1). In each case, we analyzed one simulation frame (i.e., the coordinates of all beads at a fix time-point in the simulation) for every ns within the last 400 ns of the simulation trajectories (post-equilibration), for a total of 400 frames.

For each simulation frame, we computed the following interaction contact matrices, *I,* for all possible pairs of beads (regardless of whether they are histone or DNA beads), i and j, in any nucleosome in the array:

$$I_{ij} = \begin{cases} 1, & \text{if beads } i \text{ and } j \text{ are "in contact"} \\ 0, & \text{otherwise} \end{cases}$$

Here, “in contact” is true whenever the distance between beads i and j is less than a set cutoff distance. The cut-off distance was set to the average van der Waals radius of the two beads plus 0.25 nm.

To plot the histone-tail mediated contacts, we focus on the rows of the matrix corresponding to histone beads. For each histone bead, i, the total number of contacts is computed by adding up its contacts with all other beads (DNA and histone beads) that meet different criteria (labelled “x” below), depending on the type of contact of interest:

$$M^{\text{x}}(i) = \sum_{j \text{ in x}}^{L} I_{ij}$$

Here, M is the total number of beads in the array per frame. For the “inter-nucleosome contacts” shown in Figures 2 and 4, the condition j in x is set to count contacts only if they occur among beads of different nucleosomes. For the “inter-array contacts” in Figure 5, j in x is set to count contacts only if they occur among beads of different nucleosomes and different fibers. For the “inter-nucleosome contacts” in Figure 5, j in x is set to count contacts only if they occur among beads of different nucleosomes and the same fibers. For the intra-nucleosome contacts shown in Figure S2, j in x is set to count contacts only if they occur beads of the same nucleosome.

The value of $M^{\text{x}}(i)$ is then added over for all beads that correspond to the same histone bead residue within all the different nucleosomes (e.g. for all beads corresponding to the H4K16 residue across nucleosomes 1, 2, … N)

$$C^{\text{x}}(\text{histone residue}) = \frac{1}{a} \sum_{i = \text{histone residue}}^{L} M^{\text{x}}(i)$$

Here a is a normalization constant. C^{x} is recalculated for each analysis frame, and all its values area summed. Finally, the accumulated values of C^{x} are normalized by $2Nt$, where N is the number of nucleosomes in the array, 2 considers the two copies of each tail, and t is the total number of frames used for analysis. The procedure is repeated for all the different simulations corresponding to the same experimental condition. The plots of contacts per residue display the values of $C^{\text{x}}(i)$ averaged across these different simulations.

For the plots where contacts are accumulated per tail (Fig. 2E and Fig. 4I), an additional sum is performed to add up the contacts of all the beads S of each tail:
$T^{\{H4,H3,H2A(N),H2B\}} = \sum_{i \text{ in } \{\text{H4,H3,H2A(N),H2B}\}}^{S} C^{\text{x}}(\text{histone residue})$.

Calculation of Shear Stress Relaxation Modulus using Minimal Coarse-Grained Chromatin Model

To evaluate the viscoelastic material properties of our chromatin condensates simulated with the minimal model, we compute the shear stress relaxation modulus ($G(t)$)(*30*). In the limit of zero deformation, $G(t)$ can be determined by computing the autocorrelation function of any of the off-diagonal components of the pressure tensor at equilibrium ($G(t) = (V/kT) < \sigma_{\alpha\beta}(t)\, \sigma_{\alpha\beta}(0)>$) where $\sigma_{\alpha\beta}$ is an off-diagonal component ($\alpha\beta$) of the stress tensor, V is the volume, k refers to the Boltzmann constant, and the correlation average is taken at equilibrium over all possible time origins. This correlation is directly computed during a simulation, with no significant CPU cost and no need to postprocess the trajectory using the USER-MISC LAMMPS Molecular Dynamics package. Once the relaxation modulus has been computed, the shear viscosity (η) can be straightforwardly calculated by integrating the shear stress relaxation modulus in time, using the Green-Kubo equation.

Chromatin Network Analysis

We used minimal model coarse-grained simulations to analyze the chromatin interaction network within the condensates. For each chromatin type, we performed a 5-10 microseconds simulation and selected 12 uncorrelated configurations for analysis. For each configuration, nucleosomes from every array were identified along with their neighboring nucleosomes. The interactions were classified according to face-to-face, face-to-side, and side-to-side geometries as described above. Based on the potential of mean force (PMF) for di-nucleosome interactions (*83*), nucleosomes within 130 Å were considered to be interacting through the three geometries, respectively. For each interaction, an energy of 8 kT, 7.5 kT, or 1.5 kT, respectively, was assigned to the corresponding nucleosome pair. The interaction energy for each pair of arrays was then assigned to the sum of their nucleosome-nucleosome contact energies.

To construct a geometric graph network, each array was represented as a node. Array-array interactions with energies greater than or equal to 7.5 kT then established an edge between the corresponding nodes. The interaction energy was used as the weight for the edge. We evaluated connectivity of the network through the normalized algebraic connectivity. Algebraic connectivity, also known as the Fiedler value, refers to the second smallest eigenvalue of the Laplacian matrix of a graph, which represents the overall connectivity and robustness of the network (*146*). The algebraic connectivity of the chromatin condensate graphs was normalized using a 3D lattice graph, where the degree of connections matched the maximum degree observed in the chromatin condensate. Normalized algebraic connectivity values for the 12 independent condensate configurations were averaged to describe each system.

To assess network stability, 25% of the interactions were turned off at equilibrium, and the simulation was continued. The chromatin network gradually disassembled, resembling the behavior observed in trypsin digestion experiments. Graph networks were constructed throughout the dissolution process, and the size of the largest giant component was measured.

Single-Molecule Tracking and Analysis

Single-molecule imaging was performed using a DeltaVision OMX SR system, equipped with a 60 × 1.49 NA TIRF oil immersion objective and ring-TIRF system. Imaging was conducted in epi/TIRF mode using sCMOS cameras set to 2 × 2 binning and a beam condenser to enhance

contrast. For single-molecule tracking of nucleosomal arrays, burst mode was used at 50 Hz imaging rate.

Movies were initially processed using Fiji/ImageJ (*147*) for background subtraction. The first 500 frames of each movie were discarded to ensure detection of only single nucleosome arrays. Movies were analyzed using TrackIt software (*148*). Spots were detected using a threshold factor of 2 and tracked with a tracking radius of 0.8 pixel, a minimal track length of 10, gap frames of 3 and a minimal track length before gap frame of 5.

The tracked data were analyzed via jump distance analysis to determine an effective diffusion coefficient, D_{eff}. In this analysis cumulative distributions of squared jump distances were fit with two exponential components corresponding to two effective diffusion coefficients D1, 2 with amplitudes A1, 2, respectively (*149*):

$$f_2(X) = A_1\left(1 - e^{\frac{-X}{D1}}\right) + (1 - A_1)\left(e^{\frac{-C1}{D2}} - e^{\frac{-X}{D2}}\right)\Big/\left(e^{\frac{-C1}{D2}} - e^{\frac{-C2}{D2}}\right)$$

Where $X=(x^2+y^2)/(4\tau)$ with the camera frame rate τ. Functions are normalized to account for the lower and upper limit of jump distances, C1=0 and C2=r_{max}, where r_{max} is the tracking radius. D_{eff} was defined as:

$$D_{eff} = \sum_i D_i \times A_i$$

D_{eff} is used as a measure of the overall mobility of the tracked nucleosome arrays. To assess the robustness of the data and for further error estimation, resampling was performed. Fifty percent of jumps from the pooled distribution were randomly drawn 20 times, and each distribution was fitted individually. The average value over all resampling was shown, with the error bar indicating the standard deviation of the resampling values.

To assess the asymmetry of array movement within the condensates, we initially transformed the sequential points $\{(x_0, y_0), (x_1, y_1), \cdots, (x_n, y_n), \cdots\}$ representing a trajectory in the xy plane into a series of displacement vectors $\Delta rn = (x_{n+1} - x_n, y_{n+1} - y_n)^t$. We then computed the angle between each pair of vectors Δr_n and Δr_{n+1}. The angles were normalized to 2π for visualization, with color representing the angles and values corresponding to the probability density. The Asymmetry Coefficient (AC) was determined as the $\log_2$ of the ratio between the frequencies of forward (FWD) angles (−30° to +30°) and backward (BWD) angles (150° to 210°) (*150*). A negative AC value indicates a deviation from a uniform distribution, with backward angles being predominant.

$$AC = \mathrm{Log2}\left(\frac{FWD}{BWD}\right)$$

Passive Microrheology with Optical Tweezer (pMOT).

To quantify frequency dependent viscoelasticity of the condensate, passive microrheology (*151–153*) was applied using optical tweezers (OT) implemented in a C-trap® G2 confocal system with Blulake™ software v2.7.2 (Lumicks, Netherlands). This OT system is based upon an inverted microscope where a water immersion objective (Plan Apochromat 60x, Nikon, Japan) and an oil immersion condenser attached with a temperature controller are mounted for optical trapping and imaging. A single stationary optical trap was generated by a 1064 nm infrared continuous wave

laser (overall power 1%, ~25 mW) to levitate a single spherical (diameter 1.0 µm) polystyrene bead (Spherotech$^{\text{Inc.}}$) in the sample. The trapped bead position in the x, y direction was collected using a bright field camera with ~2 ms time interval (frame rate ~500 Hz) for ~10 min at a fixed temperature of 30 °C. In addition, 78.125 kHz data were recorded by using a position sensitive detector quadrant photodiode (QPD). The C-trap system is equipped with a motorized stage including nanostage to adjust the sample position and motorized telescope to control trapping position in three dimensions; thus, it allows a probe bead to be trapped (far enough from the glass surface or edge of the condensate) inside the condensate or in solution. For all measurements ~10 µm droplets were selected (or fused together to create) to make sure the probe bead was appropriately far from the droplet edge. By measuring the time dependent position $\vec{p}_r(t)$ fluctuation of the trapped probe particle in the x-y plane, the frequency-dependent complex modulus $G^*(\omega)$ [$G'(\omega)$ (storage modulus): elastic component and $G''(\omega)$ (loss modulus): viscous component] of the condensate was obtained. Samples were prepared in a custom-made glass chamber that requires only ~15 µl volume. The glass surface was passivated by applying siliconization (Sigmacote, Sigma-Aldrich), followed by ~0.05 mg/ml bovine serum albumin (BSA) solution in double distilled water for 1 min at room temperature. The BSA concentration was optimized to limit movements of the droplets along the coverslip and to minimize excessive wetting of the surface. The chamber was sealed with UV-active adhesive to avoid evaporation of the solution and consequent alterations in concentration.

The generalized Langevin equation for the dynamics of a trapped spherical particle with mass m, and periodic displacement $\vec{r}(t) = \vec{p}_r(t) - \vec{p}_r(0)$ from the trap center, with velocity $\vec{v}(t) = \frac{d\vec{r}(t)}{dt}$ at time t, is given by:

$$m\frac{d\vec{v}(t)}{dt} + \int_0^t K(t-\tau)\vec{v}(\tau)d\tau + \kappa\vec{r}(t) = \vec{f}_R(t), \qquad (1)$$

where the integral term incorporates the memory kernel $K(t-\tau)$, κ is the optical trap stiffness and $\vec{f}_R(t)$ is the Gaussian white noise term originated from the stochastic thermal (Brownian) forces acting on the particle.

According to the Principle Equipartition of Energy at thermal equilibrium (for each dimension), $\frac{1}{2}\kappa\langle r^2\rangle = \frac{1}{2}k_BT$, where k_B is the Boltzmann constant and T is the absolute temperature. For each measurement κ was determined by measuring the mean displacement ($\langle r^2\rangle$) of the bead from the trap center, which provides an accurate measure of the trap stiffness regardless of the viscoelasticity of the material. Each day, prior to condensate measurement we calibrated the system in water to ensure that the trap stiffness (obtained from the power spectrum) was symmetrical in both x and y directions.

Laplace transform of eqn (1) results in:

$$m[s\hat{v}(s) - \hat{v}(0)] + \hat{K}(s)\hat{v}(s) + \kappa \frac{\hat{v}(s)}{s} = \hat{f}_R(s) \quad (2)$$

where s represents the frequency in the Laplace domain.

The normalized autocorrelation function (NPAF) from the bead displacement is given by:

$$A(\tau) = \frac{\langle \vec{r}(t)\, \vec{r}(t+\tau)\rangle}{\langle r(t)^2\rangle} \quad (3)$$

Multiplying both the sides of eqn (1) by $\vec{r}(0)$ and taking the ensemble average one can derive the autocorrelation function in Laplace space $\hat{A}(s) = \frac{1}{s+\frac{\kappa}{ms+\hat{K}(s)}}$

After rearranging, $\hat{K}(s) = \kappa \frac{\hat{A}(s)}{1-s\hat{A}(s)} - ms$ (4)

According to Mason and Weitz, the memory kernel is proportional to the viscosity of the fluid by the relation:

$$\hat{K}(s) = 6\pi a\hat{\eta}(s), \quad (5)$$

where a is the radius of the bead.

Therefore, $\hat{\eta}(s) = \frac{\hat{K}(s)}{6\pi a} = \frac{1}{6\pi a}\left(\frac{\kappa\hat{A}(s)}{1-s\hat{A}(s)} - ms\right)$ (6)

The complex modulus in Fourier space is related with the viscosity in Laplace space by the following relation which can be further expressed in terms of autocorrelation function $\hat{A}(\omega)$,

$$G^*(\omega) = s\hat{\eta}(s)|_{s=i\omega} = \frac{1}{6\pi a}\left(\frac{\kappa i\omega\hat{A}(\omega)}{1-i\omega\hat{A}(\omega)} + m\omega^2\right). \quad (7)$$

For a micron-size bead, $m\omega^2$ (inertia term) can be neglected for frequencies ω<<MHz. Therefore, the final expression is:

$$G^*(\omega) = \frac{1}{6\pi a}\left(\frac{\kappa i\omega\hat{A}(\omega)}{1-i\omega\hat{A}(\omega)}\right). \quad (8)$$

The frequency dependent viscosity can be derived from the imaginary part of the modulus ($G^*(\omega) = G'(\omega) + i\, G''(\omega)$):

$$\eta(\omega) = \frac{G''(\omega)}{\omega} \quad (9)$$

Data analysis was performed using a Python script adapted from previously published code . The normalized autocorrelation function was fitted with a multi exponential function:

$$A(\tau) = \sum_{i=1}^{3} G_i exp\,(-k_i \tau)^{\gamma_i} \quad (10)$$

By applying a numerical Fourier transform(*151*, *154*) $A(\tau)$ was transformed to frequency domain autocorrelation $\hat{A}(\omega)$ with the boundary condition $A(0) = 1$ and $\dot{A}(\infty) = 0$ (eqn 11):

$$-\omega^2 \hat{A}(\omega) = i\omega A(0) + (1 - e^{-i\omega t_1})\frac{A_1 - A(0)}{t_1} + \dot{A}(\infty)e^{-i\omega t_N} + \sum_{k=2}^{N}\left(\frac{A_k - A_{k-1}}{t_k - t_{k-1}}\right)(e^{-i\omega t_{k-1}} - e^{-i\omega t_k}) \quad (11)$$

Thus obtained $\hat{A}(\omega)$ was substituted in Eq. (8) to calculate the complex modulus. The $G^*(\omega)$ values were also calculated from the raw correlation values to identify potential artifacts due to fitting.

Video Particle Tracking Microrheology

Samples were prepared in 384-well glass bottom microwell plates (Brooks Life Science Systems: MGB101-1-2-LK-L). Prior to use, the plates were cleaned with 5% Hellmanex III (Hëlma Analytics), etched with 1 M KOH, and siliconized with Sigmacote (Sigma-Aldrich). On the day of the experiment, individual wells were blocked with 1% bovine serum albumin, then rinsed thoroughly with MilliQ-water. Chromatin samples were added (final concentration of 1 uM) in 25 mM Tris-Acetate pH 7.5, 150 mM KOAc, 5% glycerol, along with 175 nm-diameter carboxylate-modified fluorescent beads (<1500 beads/uL, Invitrogen: P7220). Samples were incubated inside the temperature-controlled microscope chamber at 30 °C for at least 1 hour before imaging.

Images were captured on a Lecia DMI6000 B microscope base with a Yokogawa CSU-X1 spinning disk confocal scanner unit and a 405/488/561/647 nm Laser Quad Band Set filter cube (Chroma) with a plan apo 63 or 100 × 1.40 NA oil immersion objective. Images were acquired using a Hamamatsu ImagEMX2 EM-CCD camera at 15 ms/frame using the stream acquisition function in Metamorph (Biovision) software. A total of 10,000-100,000 frames (2.5-25 min) were acquired for each acquisition, up to 6 acquisitions were made per sample per session, and at least 5 independent sessions were carried out per sample for reproducibility. In a typical acquisition, hundreds of beads were observed.

Particle tracking and calculation of mean squared displacement (MSD) was performed using MATLAB codes by Daniel Blair and Eric Dufresne (https://site.physics.georgetown.edu/matlab/code.html). Average MSD was calculated from ~10,000 individual trajectories, and smoothed using a moving average with span < 10 % of total number of frames.

Elastic (G') and viscous (G") moduli as a function of frequency (ω) were calculated from the fitted (see below) average MSD curves using the generalized Stokes-Einstein relation (GSER) as described by Mason TG (*155*) using MATLAB codes by Andrew Sun (https://github.com/andrewx101/track_analysis/releases/tag/v2.05). Viscosity (η) was calculated

(η = G"/ω) and plotted against frequency. From the viscosity plot, the mean value of the plateau at low frequency was used to estimate the zero-shear viscosity (η_0).

To calculate G' and G" using GSER, we first fit the average MSD to either a Maxwell or Jeffrey fluid model, both of which describe the relationship between Brownian motion and viscoelastic properties (for simpler and more complex viscoelastic fluids, respectively). For a Maxwell fluid(*152*), MSD is related to τ according to:

$$\mathrm{MSD}(\tau) = \Delta_0^2\left(1 + {}^{\tau}/_{\tau_c}\right)$$

where Δ_0^2 is the τ-independent offset, and τ_c is the relaxation time.

For a Jeffrey fluid, MSD is related to τ in a relationship dependent on the time regime(*156*). The behavior of a Jeffrey fluid at short times is:

$$\mathrm{MSD}(\tau) \approx \frac{6T}{\zeta_V} t\left(1 - \frac{t}{2\tau_V}\right), \tau_V \equiv \alpha \cdot \zeta_V$$

and at long times is:

$$\mathrm{MSD}(\tau) \approx \frac{6T}{\zeta_V\left(1 + \left(\frac{\zeta_M}{\zeta_V}\right)\right)}\left(t + \frac{\left(\frac{\zeta_M}{\zeta_V}\right)}{1 + \left(\frac{\zeta_M}{\zeta_V}\right)}\tau_M\right), t > \frac{\tau_M}{1 + \left(\frac{\zeta_M}{\zeta_V}\right)}$$

where τ_V and τ_M are the characteristic time points and ζ_V and ζ_M are the friction coefficient at τ_V and τ_M, respectively.

To discern whether Maxwell or Jeffrey fluid models best describe the viscoelastic properties of the chromatin condensates, we performed F tests of each model. As described in Supplementary Table S7, the 30 bp condensates were best represented as Maxwell fluids and the 25 bp condensates were best represented as Jeffrey fluids. Further supporting use of the Jeffrey fluid model for the 25 bp chromatin condensates, the pMOT analyses below showed two crossovers between G' and G", indicating viscoelastic behaviors not characterized by the simpler Maxwell fluid model. Thus, the Jeffrey and Maxwell fluid models were used to model the average MSD of the 25 bp and 30 bp condensates, respectively, in the video particle tracking experiments to calculate G' and G" using GSER.

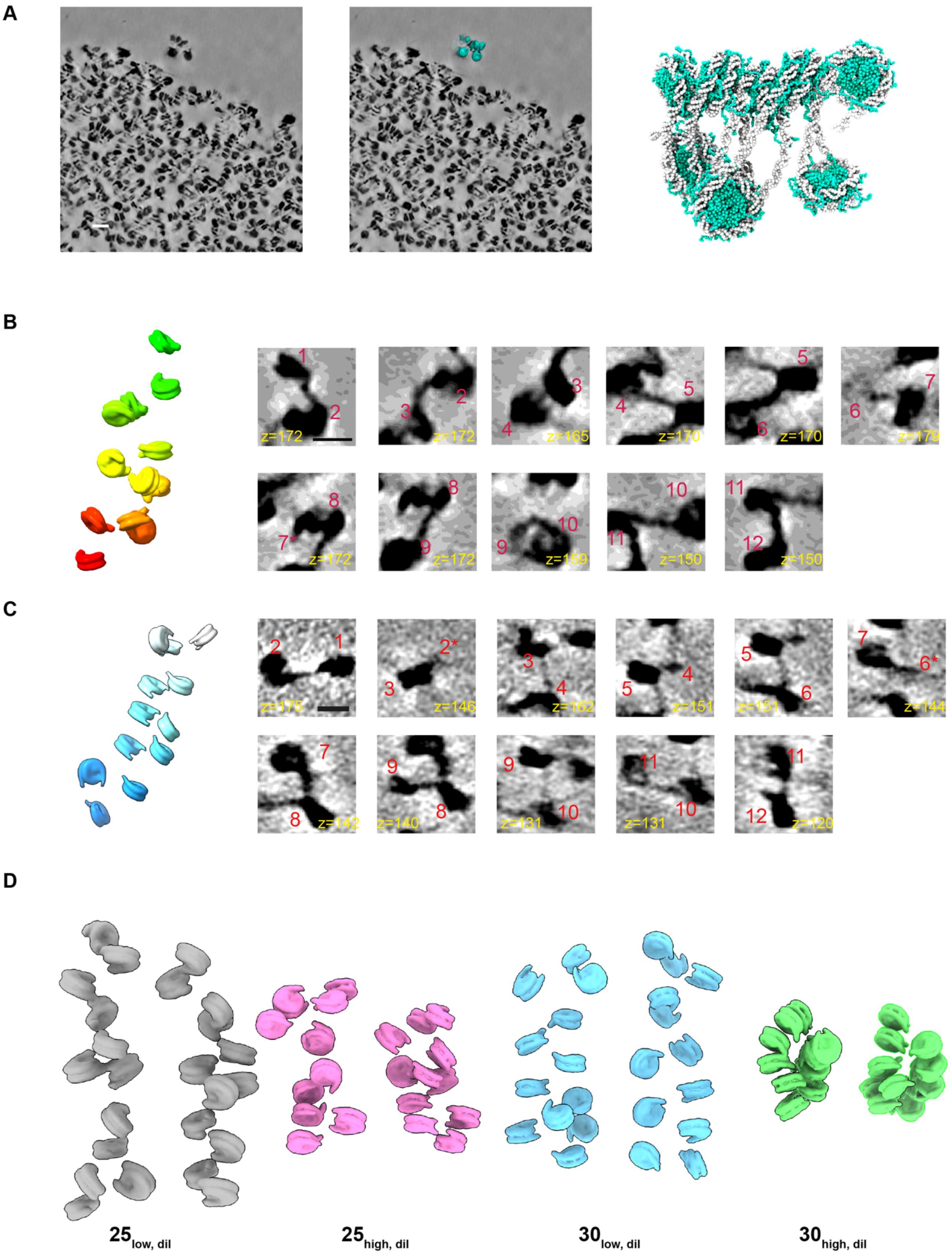
A
B
C
D
25 low, dil
25 high, dil
30 low, dil
30 high, dil

Fig. S1. Identification and tracing of nucleosome arrays in the dilute phase.

(A) Schematic of the computational approach used to reconstruct histone tails on a single chromatin array visualized by cryo-ET in high salt conditions. Left panel shows maximum projection of chromatin density in a denoised cryo-ET tomogram. Center panel shows nucleosomes placed by CATM into the density for an array outside of the condensate and then connected, scale bar is 20 nm. Right panel shows higher resolution fiber computationally reconstructed using molecular dynamics simulations steered according to nucleosome positions in the cryo-ET data.

(B-C) Stepwise tracing of nucleosome arrays in 25 bp (B) and 30 bp (C) chromatin. Left panels show assigned nucleosomes with connectivity color-coded. Right panels show sequential connections between nucleosomes from the 1st to the 12th, with red numbers indicating their order. A red number marked with a star denotes a nucleosome that is not visible in the current z-slice but appears in other slices. Yellow numbers represent their relative z-position. Scale bar: 10 nm.

(D) Representative nucleosome arrays under the specified conditions, 25 bp chromatin in low-salt dilute ($25_{low,\ dil}$) and high-salt dilute ($25_{high,\ dil}$) phases, and 30 bp chromatin in low-salt dilute ($30_{low,\ dil}$) and high-salt dilute ($30_{high,\ dil}$) phases.

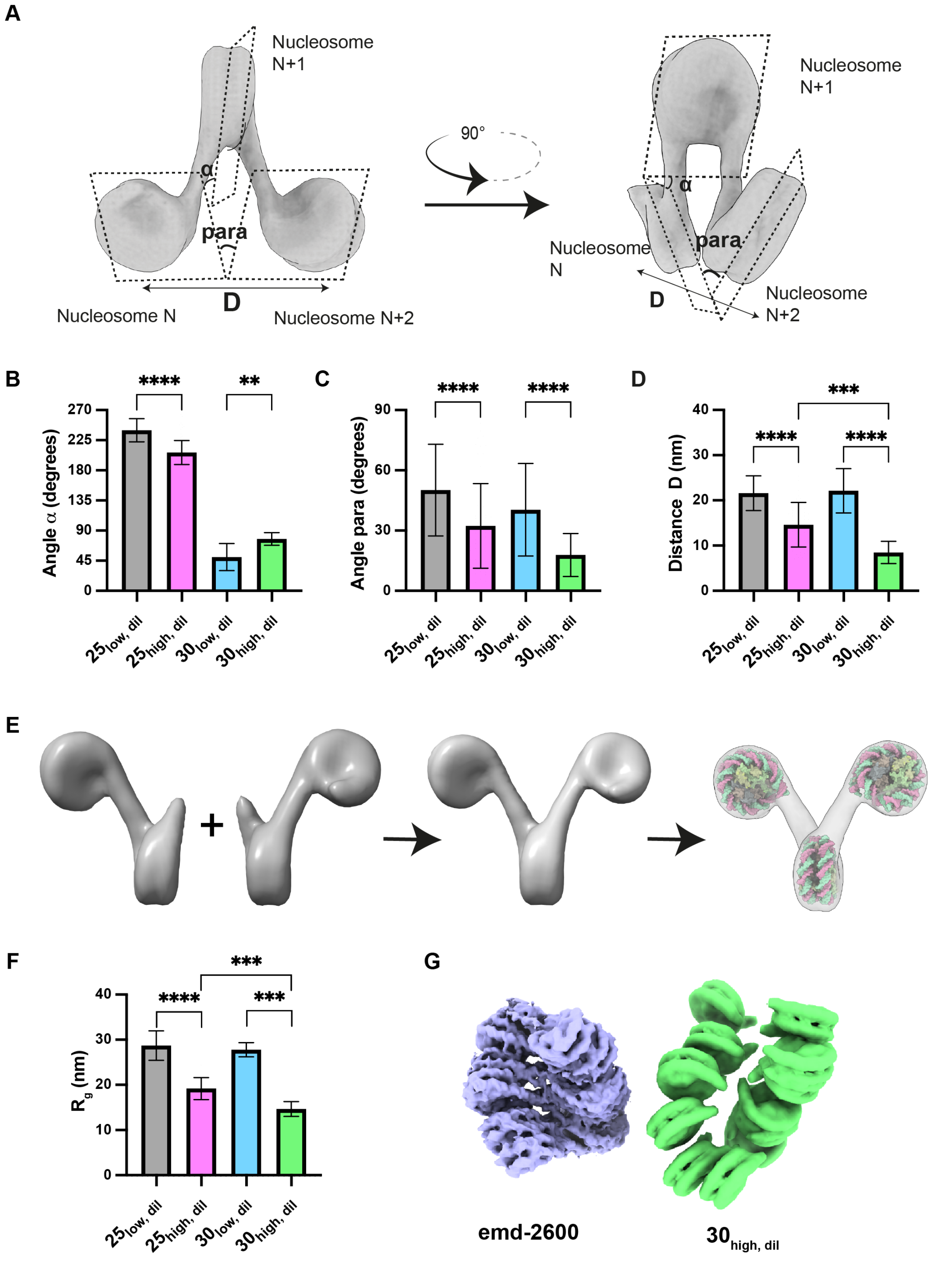

Fig S2. Modelling and analysis of nucleosome structure in the dilute phase.
(A) Schematic illustration of nucleosome features measured in this study, viewed from two perspectives.

(B-D) Statistical characterization of the dihedral angle between consecutive nucleosomes (α), the dihedral angle between nucleosomes N and N+2 (para), and the distance between nucleosomes N and N+2 (D). Labels indicate chromatin type (25 bp, 30 bp), salt conditions (low, high) and phase (dilute (dil), condensate (cond)). $25_{low, dil}$ is gray, $25_{high, dil}$ is magenta, $30_{low, dil}$ is blue, and $30_{high, dil}$ is green. Statistical comparisons were performed using the Kruskal–Wallis test (n≥45).
(E) Modelling of the tri-nucleosome models for visualization. Two identical subtomogram-averaged di-nucleosome structures were aligned at the central nucleosome to generate a tri-nucleosome configuration. On the right, mononucleosome structures (PDB: 6pwe) were fitted into the nucleosome density to enhance visualization.
(F) R_g measurements for traced 25 bp and 30 bp chromatin in low salt and high salt conditions. Error bars represent the standard deviation of the measurements. Statistical comparisons were performed using the Kruskal–Wallis test (n≥10).
(G) Left image shows the 11 Å resolution single-particle cryo-EM map of a 30 bp DNA linker length chromatin fiber in the presence of linker histone H1.4, crosslinked with glutaraldehyde in 0 mM salt conditions (emd-2600). Right image shows one of the traced 30 bp chromatin fibers in the high salt dilute phase. Both structures are 12-mer nucleosome arrays.

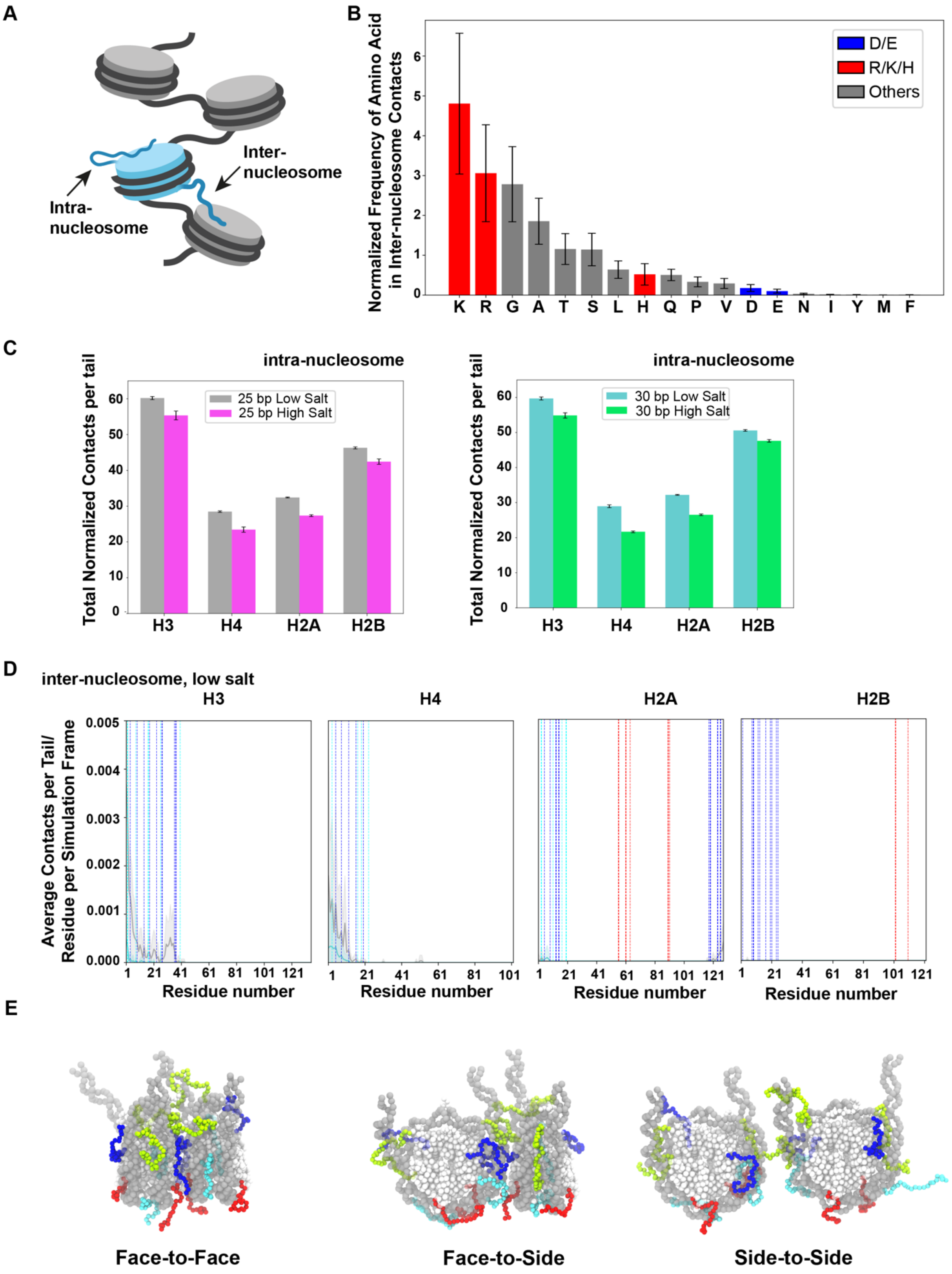
A
Inter-
nucleosome
Intra-
nucleosome
B
Normalized Frequency of Amino Acid
in Inter-nucleosome Contacts
D/E
R/K/H
Others
0
1
2
3
4
5
6
K R G A T S L H Q P V D E N I Y M F
C
intra-nucleosome
Total Normalized Contacts per tail
25 bp Low Salt
25 bp High Salt
30 bp Low Salt
30 bp High Salt
0
10
20
30
40
50
60
H3 H4 H2A H2B
D
inter-nucleosome, low salt
H3
H4
H2A
H2B
Average Contacts per Tail/
Residue per Simulation Frame
0.000
0.001
0.002
0.003
0.004
0.005
Residue number
1 21 41 61 81 101 121
E
Face-to-Face
Face-to-Side
Side-to-Side

Fig. S3. Simulation of histone tail interactions in the dilute phase.

(A) Schematic illustration of histone tail-mediated intra-nucleosome and inter-nucleosome interactions.

(B) Normalized frequency of amino acid pair inter-nucleosome contacts involving different residue types.

() Total number of contacts mediated by the N-terminal histone tails (H3, H4, H2A(N), and H2B) of one nucleosome and the DNA and histones of the same nucleosome (intra-nucleosome nucleosome) computed from molecular dynamics simulations where nucleosomes are restrained to maintain their cryo-ET positions. Number of contacts is defined as the total number of amino acid–phosphate and amino acid–amino acid pairs closer than a cutoff distance (see Methods) per tail type per nucleosome averaged across the different arrays and the simulation trajectory. Error bars represent one standard deviation from the mean. Plots compare results for 25-bp (left) and 30-bp (right) chromatin fibers under high and low salt conditions.

(D) Number of tail-mediated inter-nucleosome contacts in low salt conditions. Data for 25 bp and 30 bp fibers is pink and green, respectively. Blue and cyan vertical lines show the positions of lysines and arginines in the tails, respectively. Shading indicates the standard deviation from the mean.

(E) Schematic chemically specific coarse-grained representation of tail-mediated inter-nucleosome interactions among nucleosome pairs interacting face-to–face (left), face-to–side (middle), and side-to–side (right). DNA beads are yellow, histone core beads are grey, H3 tail beads are green, H4 tail beads are blue, H2A(N) beads are red, and H2B beads are cyan.

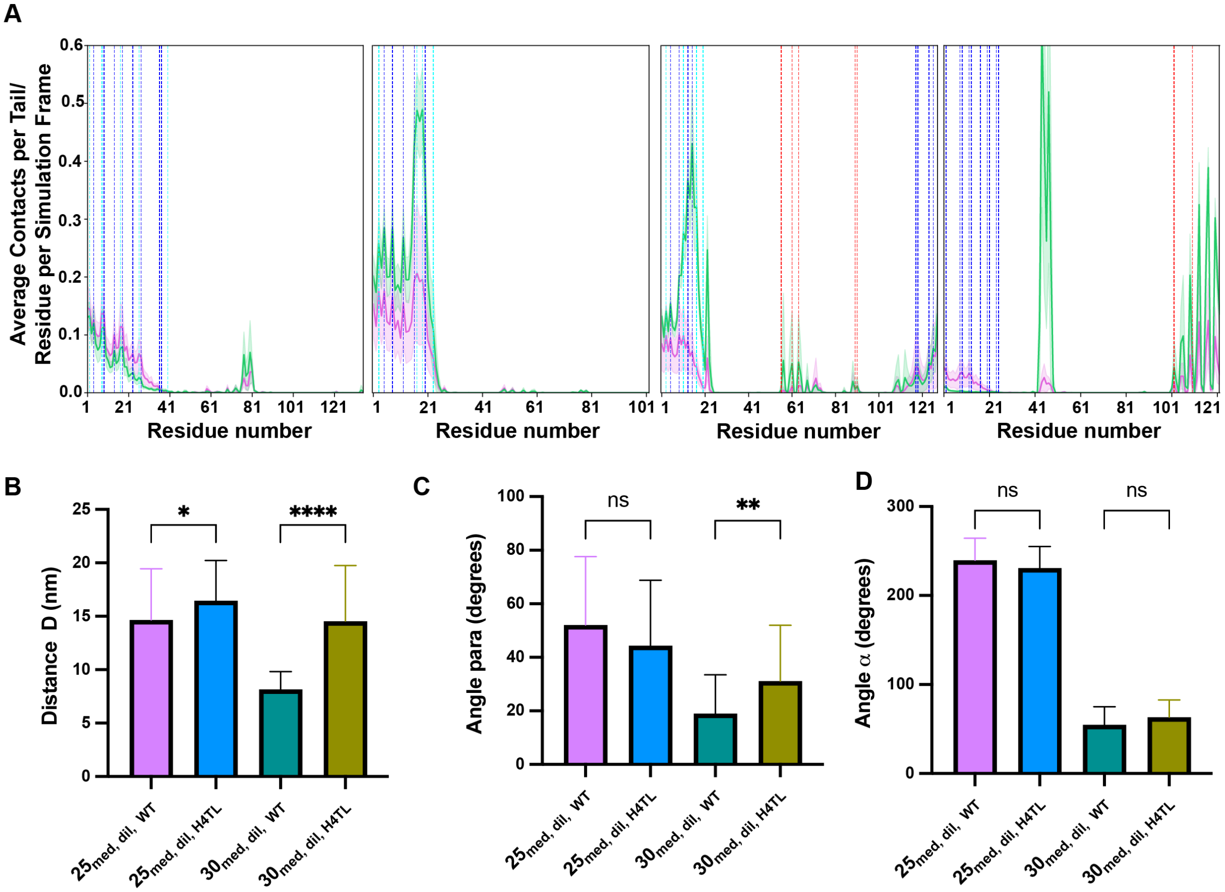

Fig. S4. Computer simulations of histone tail interactions and cryo-ET analysis of histone H4 tail deletions.

(A) Number of tail-mediated inter-nucleosome contacts in high salt conditions in the dilute phase. Data for 25 bp and 30 bp fibers is pink and green, respectively. Blue and cyan vertical lines show the positions of lysines and arginines in the tails, respectively. Shading indicates standard deviation from the mean.

(B–D) Quantification of the inter-nucleosome distance D (B), dihedral angle para (C), and angle α (D) for nucleosome arrays reconstituted with either wild-type histone octamers or octamers lacking the H4 tail. Statistical comparisons were performed using the Kruskal–Wallis test (n=10).

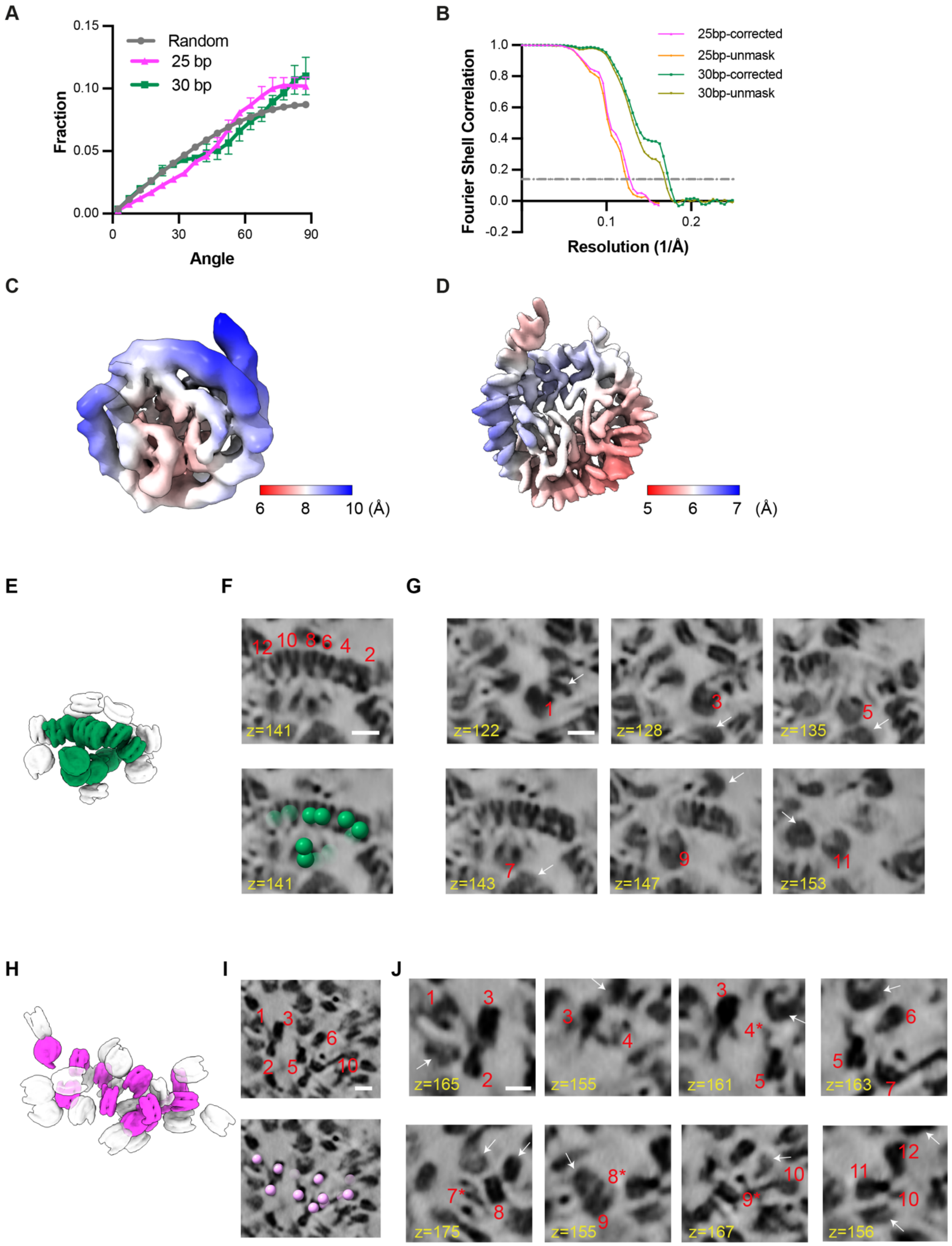
A
Fraction
Angle
Random
25 bp
30 bp
B
Fourier Shell Correlation
Resolution (1/Å)
25bp-corrected
25bp-unmask
30bp-corrected
30bp-unmask
C
6 8 10 (Å)
D
5 6 7 (Å)
E
F
G
H
I
J
z=141
z=122
z=128
z=135
z=143
z=147
z=153
z=165
z=155
z=161
z=163
z=175
z=167
z=156

Fig. S5. Subtomogram averaging and nucleosome array tracing within chromatin condensates.

(A) Angular distribution between the nucleosome plane normal and the electron beam (Z-axis) for 25 bp (magenta), 30 bp (green) chromatin. Analogous distribution of angles for a bath of randomly oriented nucleosomes is shown in grey.
(B) Fourier shell correlation (FSC) between two independently processed halves of the datasets for 25 bp and 30 bp chromatin condensates.
(C–D) Subtomogram-averaged nucleosome structures reconstructed from 25 bp (C) and 30 bp (D) chromatin condensates, colored by local resolution.
(E–J) Representative examples of nucleosome array tracing in 25 bp (E–G) and 30 bp (H–J) chromatin condensates. Left panels (E, H) show colored nucleosome array models surrounded by white nucleosomes. Middle panels (F, I) display central slices with nucleosome labels (top) and sphere representations (bottom) of the traced arrays. Right panels (G, J) illustrate the sequential connections of nucleosomes from the 1st to the 12th, with red numbers indicating order. A red number accompanied by a star marks a nucleosome that is absent in the current z-slice but present in other slices. Yellow numbers denote the relative position along the Z-axis, scale bar is 10 nm.

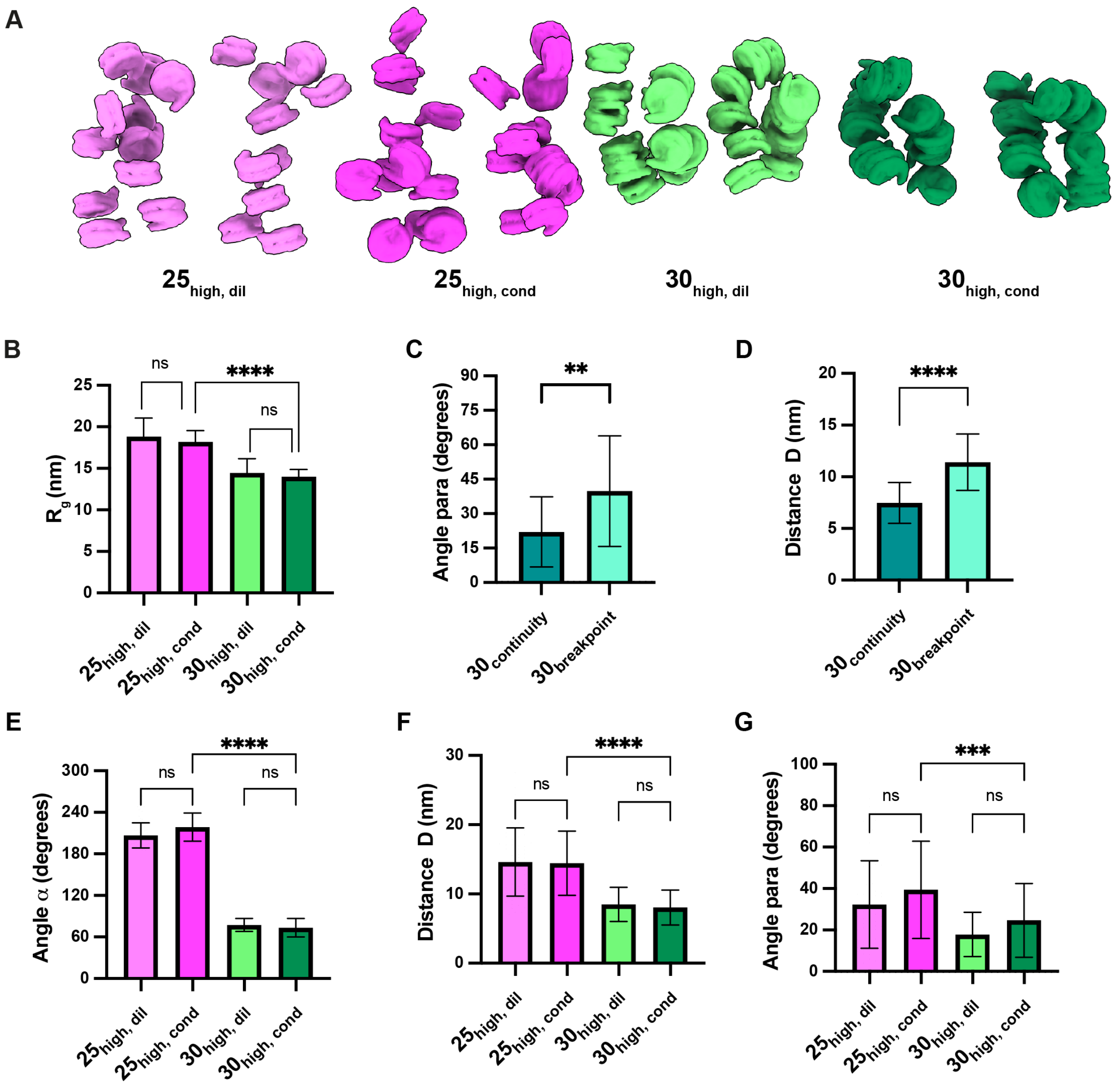

Fig. S6. Comparison of 25 bp and 30 bp chromatin structures in dilute and condensed phases.

(A) Structural comparison of nucleosome arrays for 25 bp and 30 bp chromatin under high salt conditions. Two traced arrays are shown of 25 bp chromatin in the dilute phase ($25_{high, dil}$, light magenta) and condensed phase ($25_{high, cond}$, magenta), alongside two traced arrays of 30 bp chromatin in the dilute phase ($30_{high, dil}$, light green) and condensed phase ($30_{high, dil}$, green).
(B) Radius of gyration of nucleosome arrays in the indicated conditions. Statistical comparisons were performed using the Kruskal–Wallis test (n=10).
(E-G) Comparison of dihedral angle α (E), distance D (F), and para (G) for 25 bp and 30 bp chromatin at the indicated conditions.

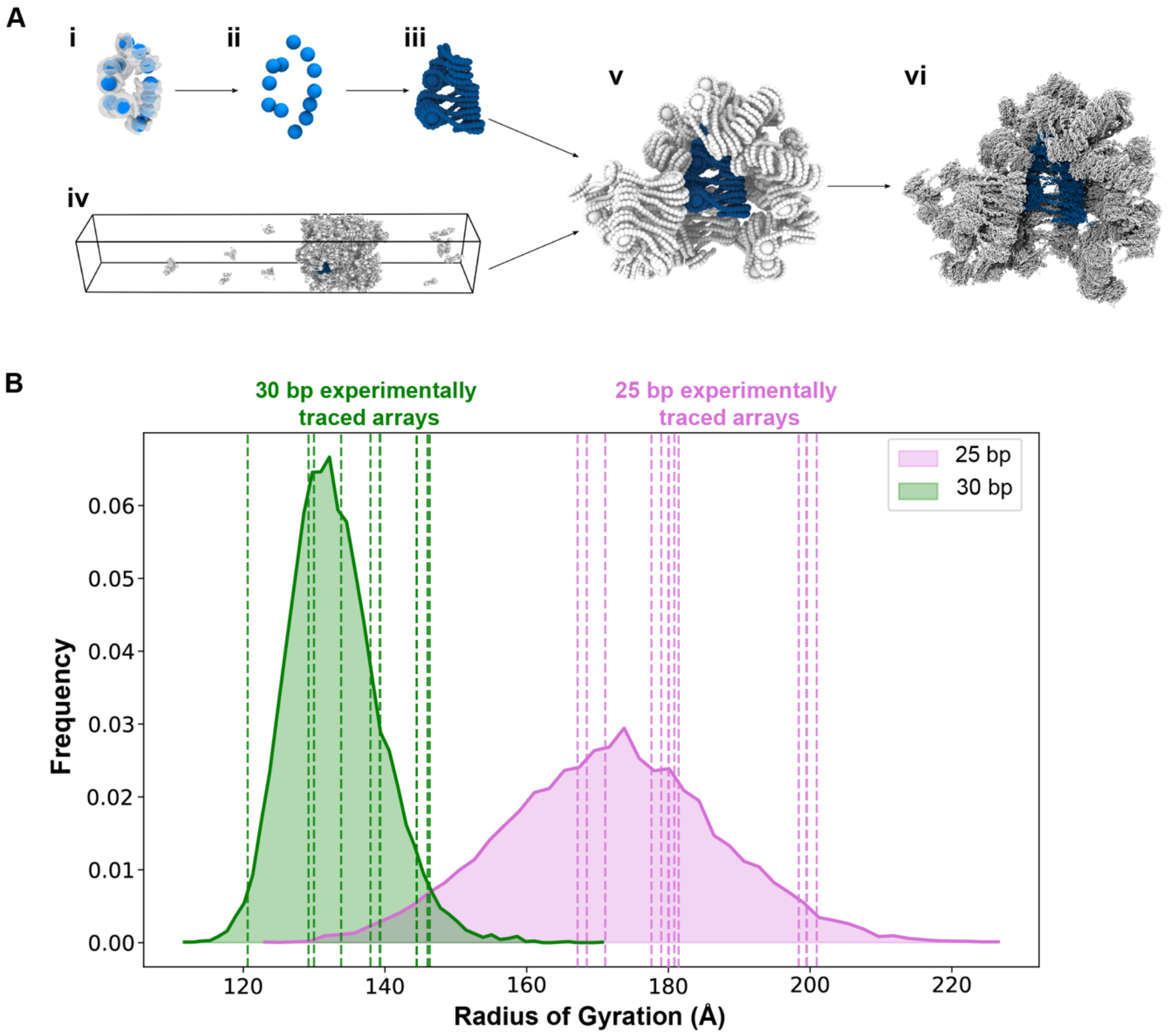

Fig. S7. Simulations to Probe Histone Tail Interactions inside Chromatin Condensates

(A) Schematic of the multiscale simulation protocol used to enhance the resolution of cryo-ET data for chromatin fibers in condensates down to the amino acid and nucleotide level. Top: Cryo-ET traced chromatin fibers (i) are compared, based on the geometric centers of their nucleosomes (ii), with individual chromatin structures extracted from the simulations (iii). Simulation structures with lower RMSD values (closer matches) to their cryo-ET counterparts are given higher scores. The top-scoring structure from the simulations, highlighted in blue, is selected (iv). These simulations of chromatin condensates are performed using a minimal coarse-grained chromatin model. Panel iv shows part of the slab simulation box with the chromatin condensate coexisting with the dilute phase. Each fiber is represented with one sphere per 5 bp and one ellipsoid for the histone core. Using the top-scoring structure from the condensate simulations, a cluster of neighboring chromatin arrays in contact with the top-scored structure is identified (v, colored grey). This cluster is then backmapped from the minimal coarse-grained model to the chemically specific model, enabling reconstruction to the amino acid and nucleotide level (vi). Finally, molecular dynamics simulations are performed on the high-resolution cluster, applying restraints to preserve the structures of the interacting arrays. This approach allows us to

sample the dynamical behavior of histone tails and perform statistical analyses on the inter-nucleosome interactions they mediate.
(B) Distribution of chromatin radius of gyration in *in silico* chromatin condensates. Radius of gyration, R_g, for chromatin arrays inside simulated condensates of the 25 bp (magenta) and 30 bp (green) chromatin arrays. Each shaded region represents the distribution of R_g values; dashed vertical lines indicate normalized R_g values of experimentally traced arrays in each case. Note that the same coarse grained simulation trajectories used for the analysis here were also reported in (*83*).

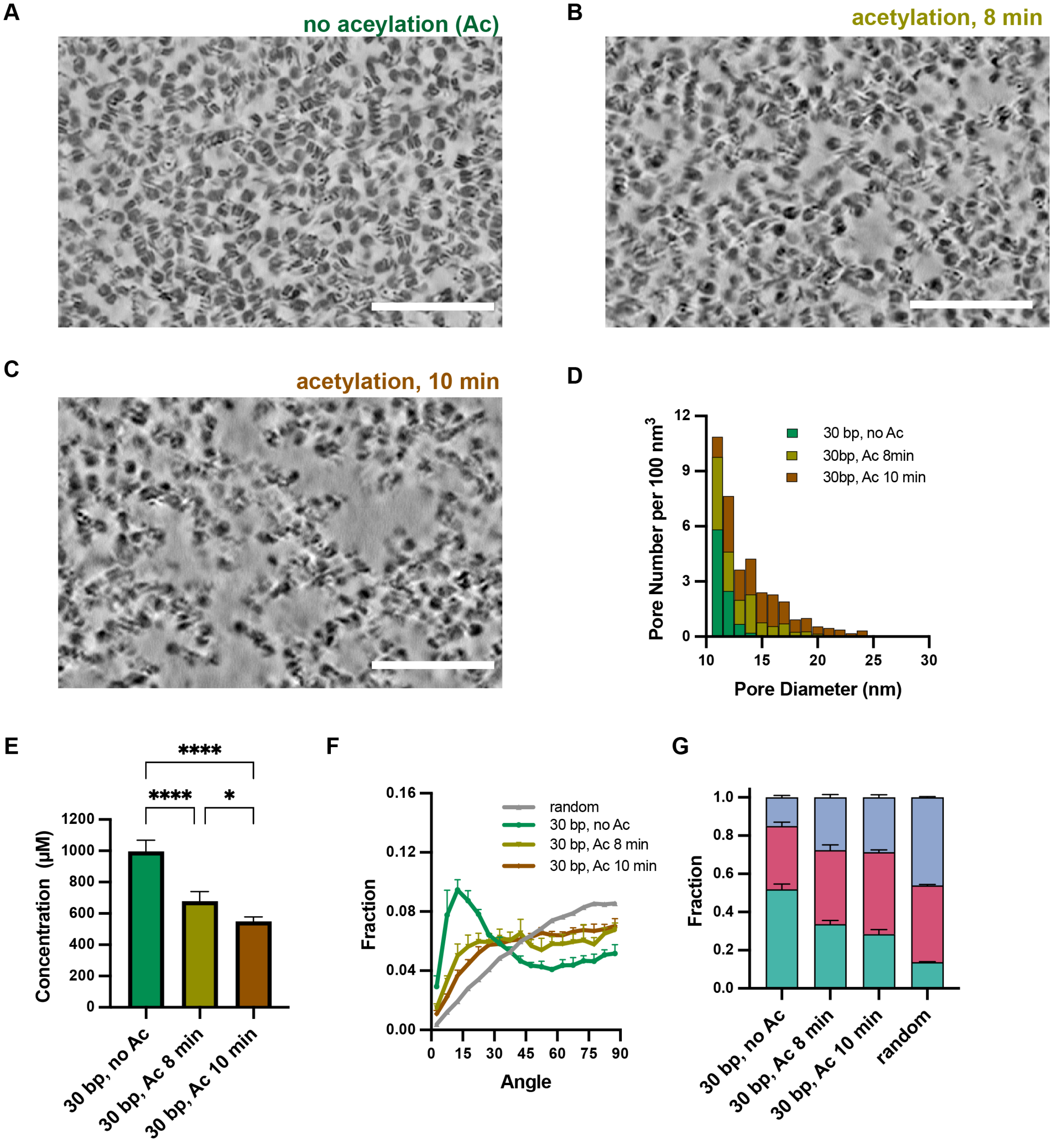

Fig. S8. Acetylation induces pore formation in condensates and remodels chromatin structure

(A–C) Representative cryo-ET images of chromatin captured by freezing before acetylation (A), and at 8 minutes (B) and 10 minutes (C) of the acetylation reaction. Scale bar: 100 nm.
(D) Quantification of pore sizes by diameter in the indicated condensates.
(E) Nucleosome concentration in the condensates measured before and after acetylation.
(F) Distributions of nearest di-nucleosome orientations in a randomized system (grey) and in the indicated chromatin samples (green, yellow, red for 0, 8 and 10 minutes of acetylation).

(G) Proportions of face-to-face (green), face-to-side (red), and side-to-side (blue) nucleosome contacts in random distributions, and in condensates before and after acetylation.

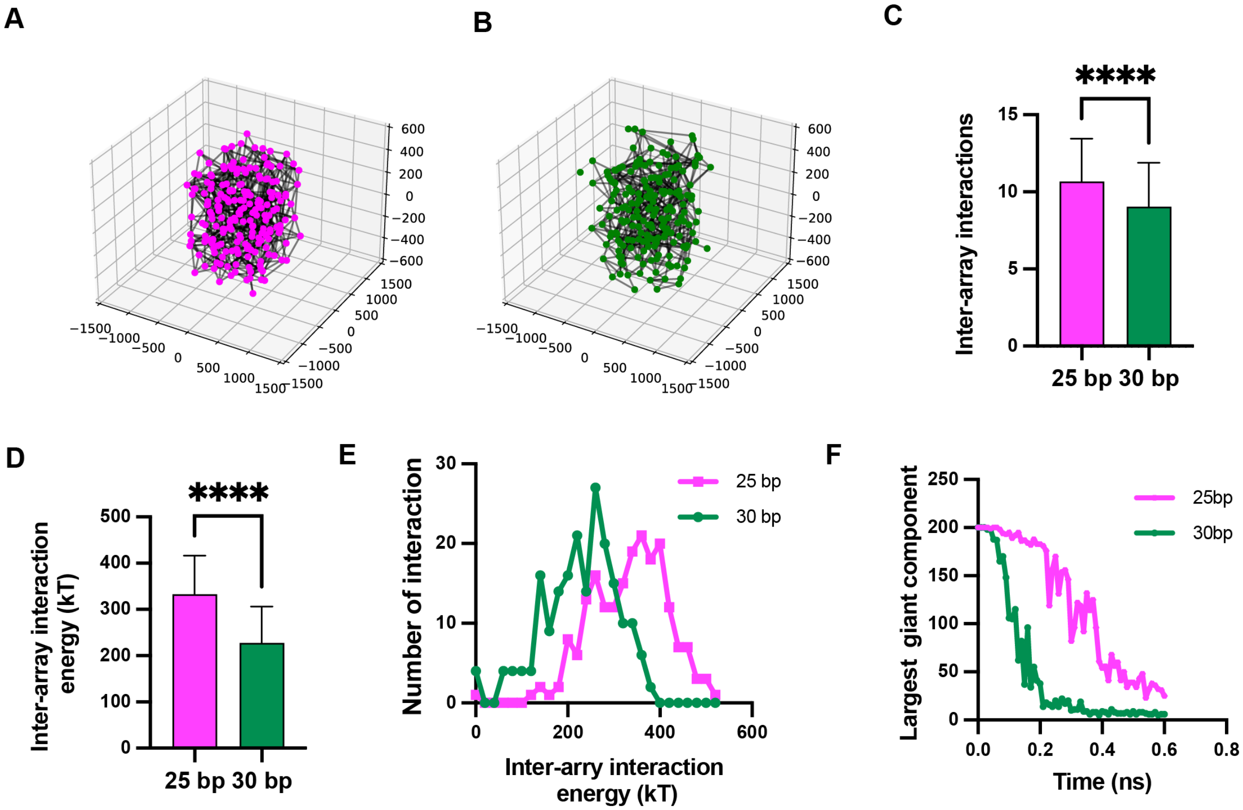

Fig. S9. Chromatin with 25 bp linkers forms a denser and more connected network compared to chromatin with 30 bp linkers, as shown by coarse-grained simulations.

(A-B) Graph network representations of simulations for 25 bp (A) and 30 bp (B) chromatin. Nodes represent nucleosome arrays, while edges denote interactions between these arrays.

(C) Number of inter-array interactions for each nucleosome array in the simulation. Error bars indicate standard deviation of 196 independent measurements.

(D-E) Association energy between the nucleosome arrays. In D, error bars indicate standard deviation of 196 independent measurements.

(F) Reduction in size of the largest cluster after deactivating 25% of the interactions between nucleosome arrays.

(C) Number of inter-array interactions for each nucleosome array in the simulation. Error bars indicate standard deviation of 196 independent measurements.

(D-E) Association energy between the nucleosome arrays. In D, error bars indicate standard deviation of 196 independent measurements.

(F) Reduction in size of the largest cluster after deactivating 25% of the interactions between nucleosome arrays.

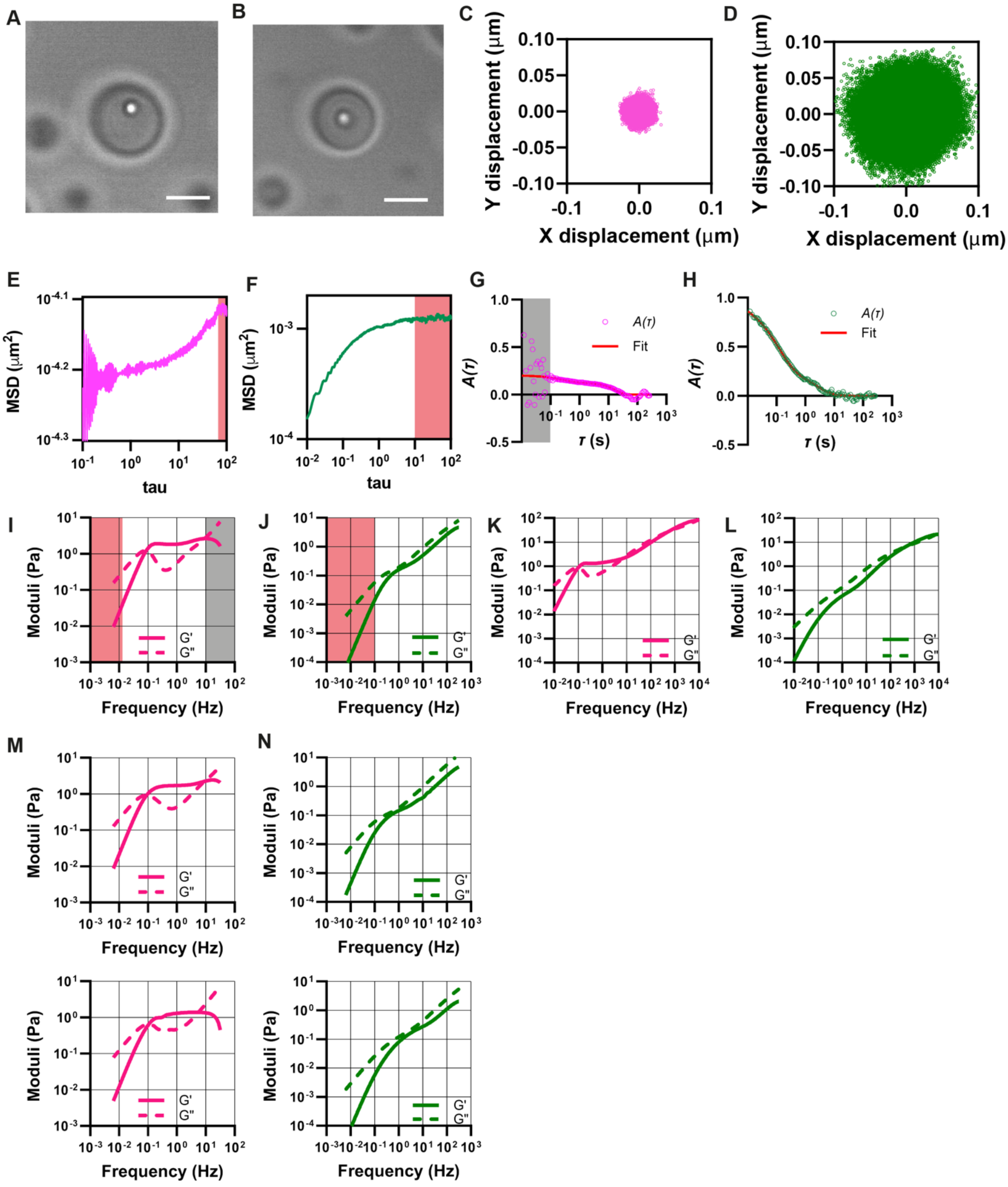

Fig. S10. Measuring chromatin viscoelasticity using PMOT.
(A-B) Representative brightfield images of 25 bp (left) and 30 bp (right) chromatin condensates. Within each is an optically tapped 1 μm bead that is used for PMOT. Scale bar = 5 μm. Same representatives are used for panels (A-J) to demonstrate PMOT methodology.
(C-D) Representative XY displacements of the trapped motions of the optically trapped 1 μm bead within 25 bp (left; magenta) and 30 bp (right; green) chromatin condensates. For both, the XY displacements across 300 s are shown.

(E-F) Representative mean-squared displacements of the trapped motions of the optically trapped 1 µm bead within 25 bp (left; magenta) and 30 bp (right; green) chromatin condensates. Rose-shaded regions at high lag times represent time scales at which the bead motions are fully determined by the optical traps and not the viscoelastic properties of the encompassing chromatin condensates.
(G-H) Representative autocorrelation functions of the trapped motions of the optically trapped 1 µm bead within 25 bp (left; magenta) and 30 bp (right; green) chromatin condensates. Red lines indicate the fitted autocorrelation functions. In panel (G), gray-shaded regions in low lag times represent the time scales at which the unfitted autocorrelation functions are error-prone.
(I-J) Representative elastic/storage (G') and viscous/loss (G") moduli of 25 bp (left; magenta) and 30 bp (right; green) chromatin condensates, calculated from fitted autocorrelation functions in panels (G-H). The rose and gray-shaded frequencies correspond to the rose and gray-shaded lag times in panels (E-H) to indicate the error-prone regions.
(K-L) Representative elastic/storage (G') and viscous/loss (G") moduli of 25 bp (left; magenta) and 30 bp (right; green) chromatin condensates, obtained from similar processing of 78 kHz measurements collected using the quadrant photodetector (QPD) of the LUMICKS C-trap.
(M-N) Additional representative elastic/storage (G') and viscous/loss (G") moduli of 25 bp (left; magenta) and 30 bp (right; green) chromatin condensates determined using the standard bright field camera (as in panels A-J).

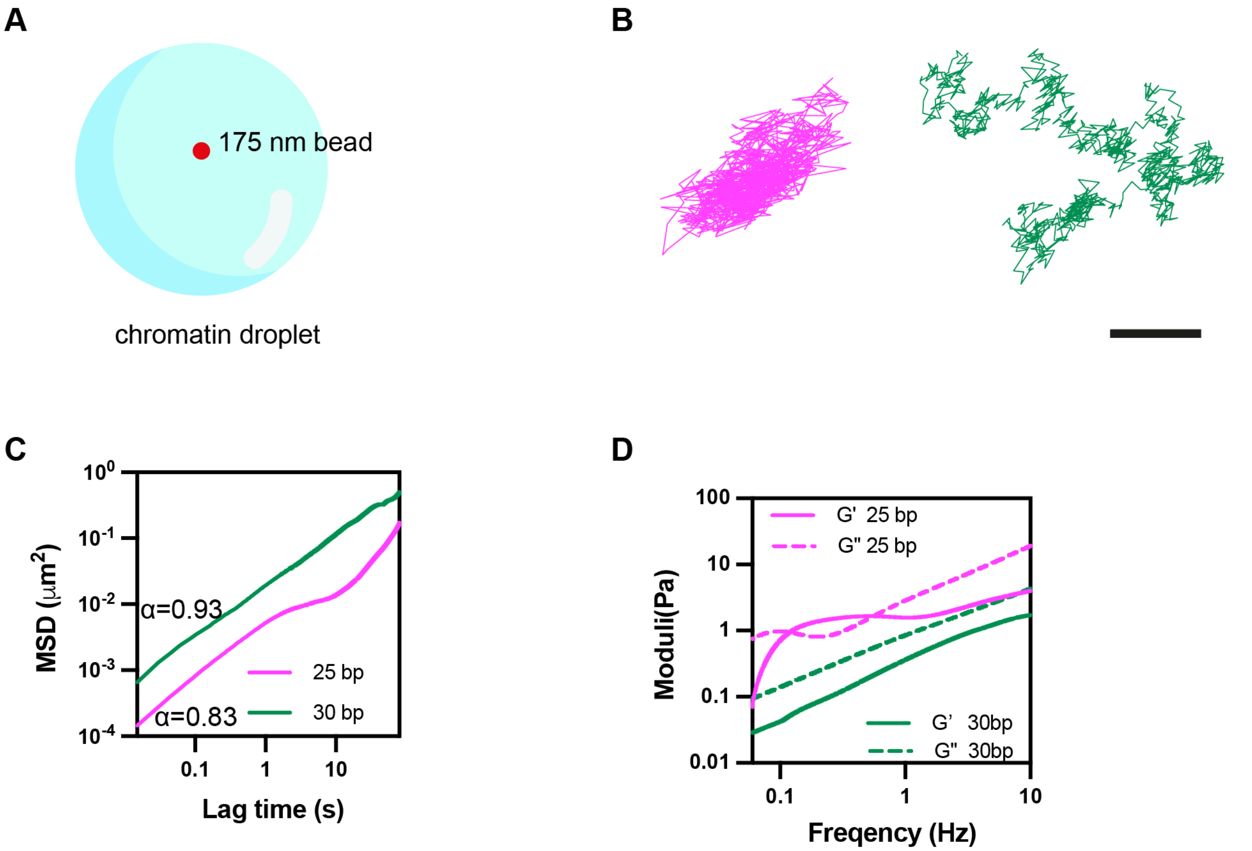

Fig. S11. Measuring chromatin viscoelasticity modulus with video particle tracking microrheology.

(C) Mean Squared Displacement (MSD) analysis for beads tracked in 25 bp (magenta) and 30 bp (green) chromatin condensates. MSD was averaged from ~10, 000 individual trajectories. Alpha indicates the scaling exponent of diffusion (slope of the MSD vs lag time curve) for short lag times.

(D) Elastic/storage (G') and viscous/loss (G") moduli of 25 bp (magenta) and 30 bp (green) chromatin condensates, calculated from MSD data in panel (C) using the Jeffery model (25 bp) or Maxwell model (30 bp) (Supplementary Table 7).

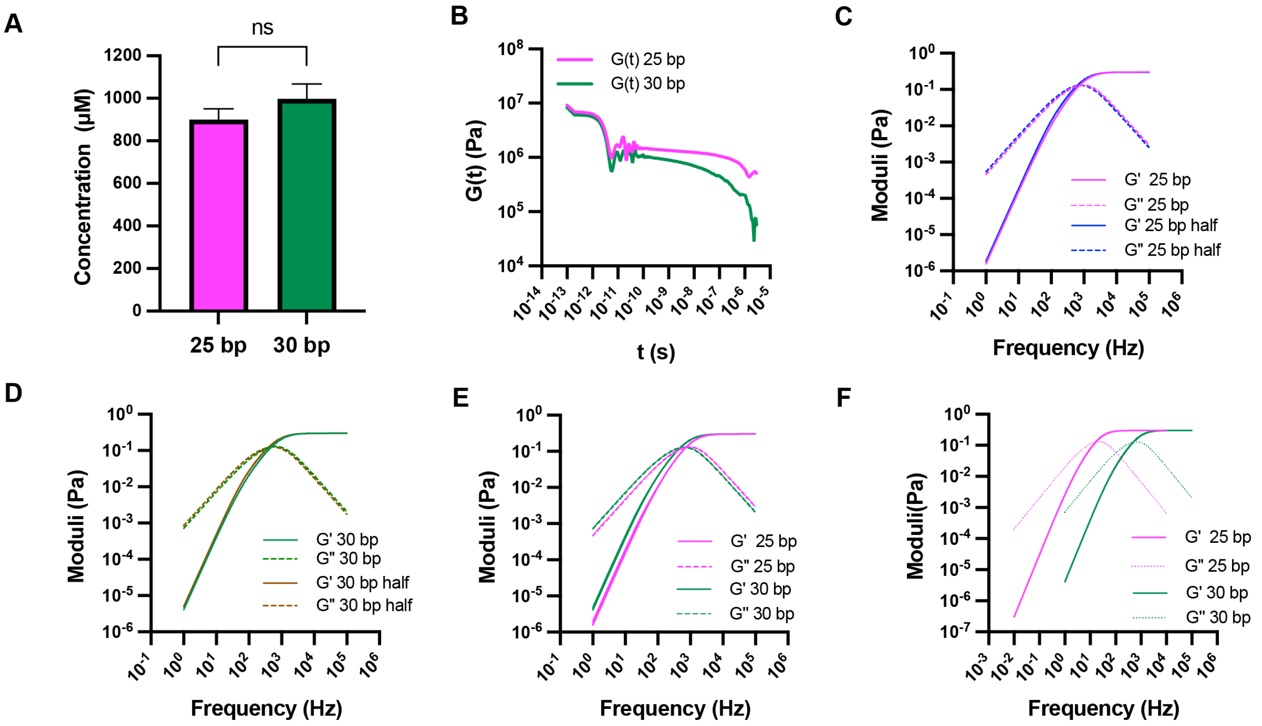

Fig. S12. Quantification of nucleosome concentration and viscoelastic moduli in chromatin condensates.

(A) Quantification of nucleosome concentration in 25 bp and 30 bp chromatin condensates. Statistical analysis was performed using ordinary one-way ANOVA ($n = 4$). We note that in our earlier estimation of nucleosome density based on fluorescence intensity, we described 25 bp condensates as more dense than 30 bp condensate ((*33*))We believe the current direct measurement with cryo-ET data is more reliable given potential environmental effects on intrinsic fluorophore intensity.

(B) Stress relaxation modulus G(t) calculated directly from stress tensor autocorrelations in coarse-grained simulations using the Green–Kubo formalism(*30*).

(C–D) Validation of the direct viscoelasticity measurement from the coarse-grained MD simulations using a graph-based Rouse-Zimm model (*5*). The chromatin network was extracted from coarse-grained simulations (see Fig. S9) and split into two halves based on Z-coordinate thresholds. For both the 25 bp and 30 bp chromatin simulations the two half-data sets yielded nearly identical G′ and G″ profiles to the full dataset, confirming that the network size in the full dataset was sufficient for this analysis.

(E) Overlaid plots of computed storage (G′) and loss (G″) moduli for 25 bp and 30 bp chromatin, averaged with 3 independent snapshots from the simulations using the graph-based Rouse-Zimm model (*5*). Computations only varied in the connectivity matrix used for the two condensates. Error is indicated by the thickness of the line. We note that this formalism is hard-wired to provide single crossover between G' and G'', and thus is a valuable but imperfect description of our data.

(F) Computation of viscoelastic moduli after scaling the friction coefficients in the graph-based Rouse-Zimm model (*5*) using experimentally measured viscosities for 25 bp and 30 bp chromatin condensates, averaged with 3 independent snapshots from the simulations. Error is indicate by the thickness of the line.

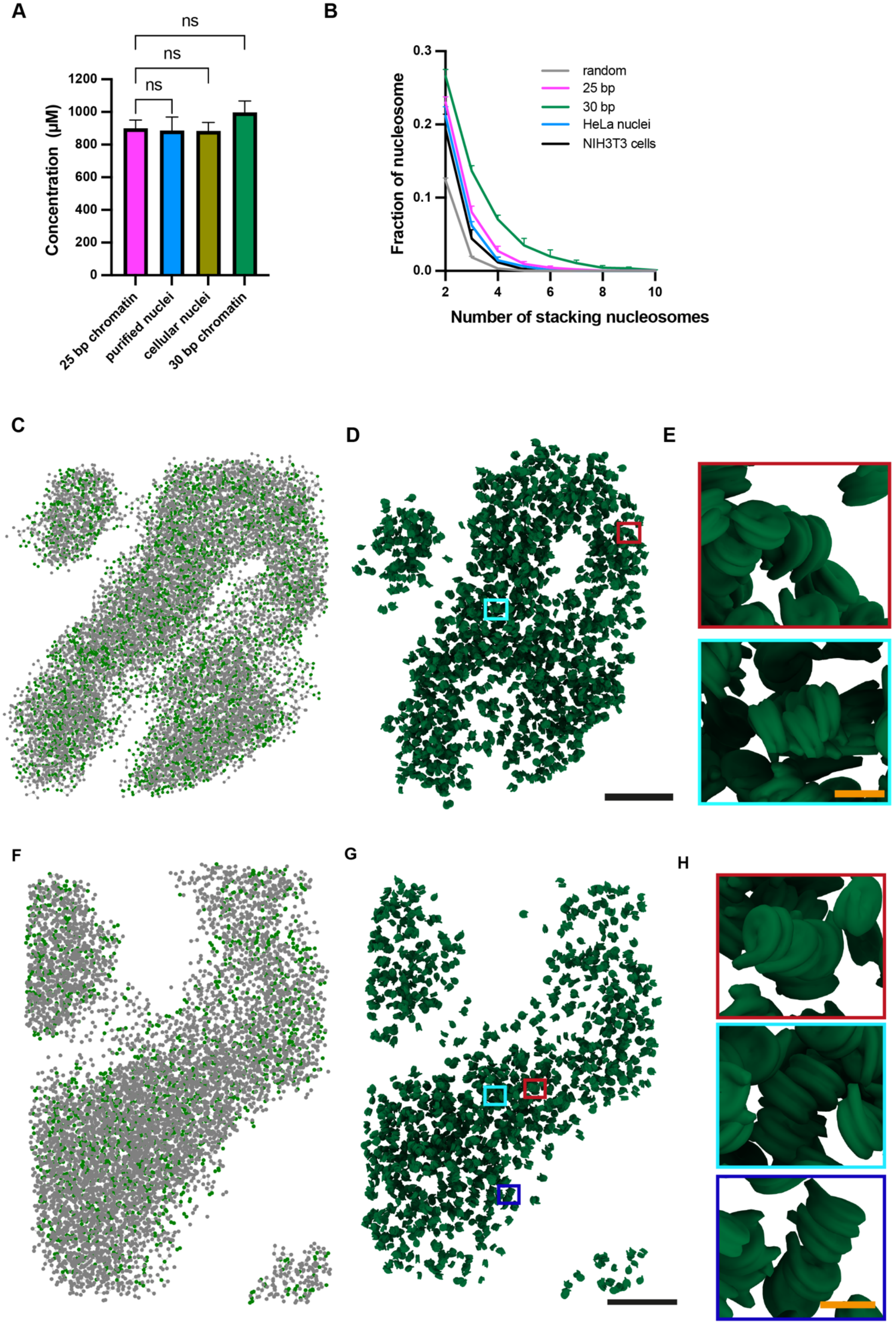
A
ns
ns
ns
1200
1000
800
600
400
200
0
Concentration (μM)
25 bp chromatin
purified nuclei
cellular nuclei
30 bp chromatin
B
0.3
0.2
0.1
0.0
Fraction of nucleosome
2
4
6
8
10
Number of stacking nucleosomes
random
25 bp
30 bp
HeLa nuclei
NIH3T3 cells
C
D
E
F
G
H

Fig. S13. Quantification of nucleosome concentration and stacking interactions.
(B) Quantification of the fraction of nucleosomes belonging to continuously stacked nucleosome fibers in the indicated systems.
(C, F) To investigate potential spatial relationships between the stacked nucleosome structures, we constructed a graph network for chromatin from HeLa nuclei (C) and NIH3T3 cellular tomograms (F). We analyzed the attribute assortativity coefficient (ASC) of the two classes (face-to-face stacked shown as green dots and all other contact types shown as grey dots). ASC quantifies the tendency of nodes with similar attributes to connect, ranging from −1 (indicating minimal connectivity between similar nodes) to +1 (indicating strong preferential connectivity). Our analysis yielded a near-neutral ASC value of 0.148 ± 0.009 and 0.157 ± 0.002 for HeLa nuclei and NIH3T3 cellular chromatin respectively, suggesting the stacking of nucleosomes slightly favors clustering.
(D, G) Stacked nucleosomes in representative HeLa nuclei (D) and NIH3T3 cellular tomograms (G). Scale bars represent 100 nm.
(E, H) Expanded view of a few examples of stacked nucleosomes in panels D and G. Scale bar are 10 nm.

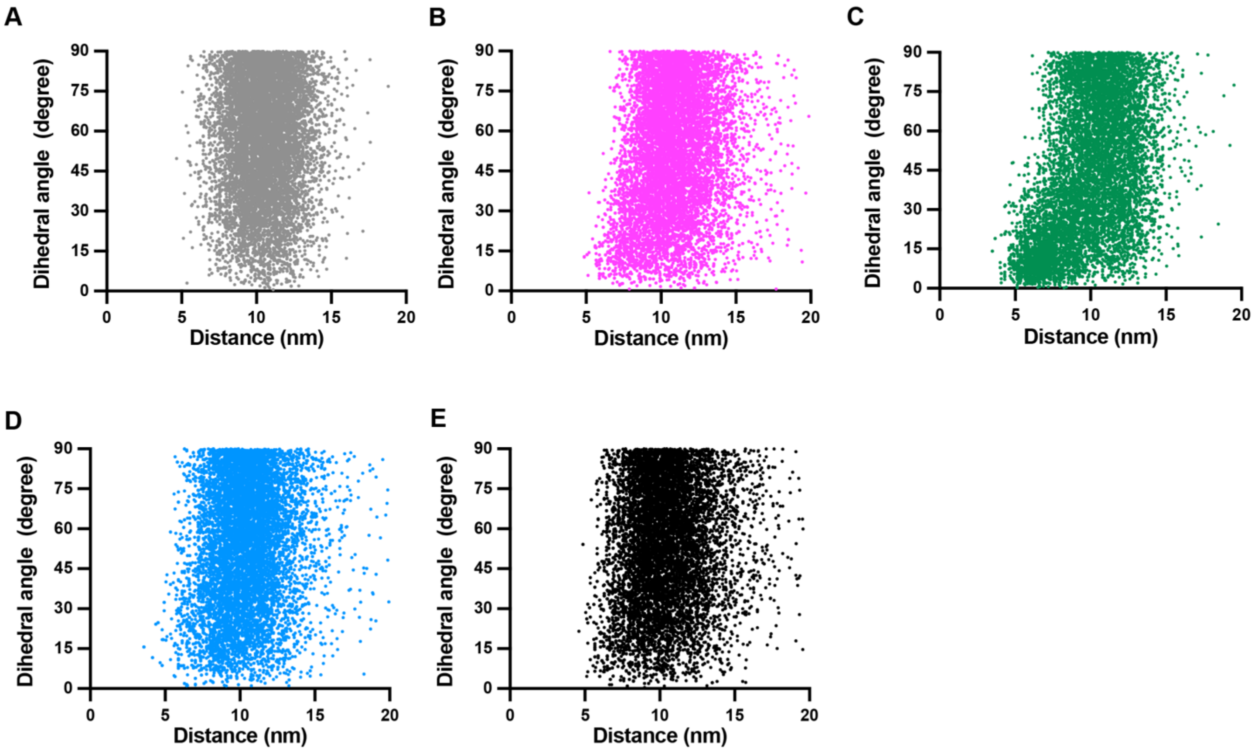

Fig. S14. Quantification of nucleosome dihedral angles and distance distributions.
(A–E) Analysis of the relationship between dihedral angles and inter-nucleosome distances for neighboring nucleosomes (within 20 nm) in randomly distributed nucleosomes (A), 25 bp chromatin condensates (B), 30 bp chromatin condensates (C), isolated HeLa cell nucleus chromatin (D), and NIH3T3 cell chromatin (E). Compared to the random distribution, the 25 bp chromatin and both cellular chromatin samples show a shift toward longer distances (15–20 nm) and modest enrichment at low angles with short distances (5–8 nm). In contrast, the 30 bp chromatin exhibits a pronounced enrichment of low dihedral angles at short distances, indicating a higher prevalence of face-to-face stacking interactions.

Table S1. Summary of Nucleosome Arrays and Nucleosomes Used in the Chemically Specific Model Simulation.

condition	Number of Nucleosome Arrays (Number of Neighboring Arrays)	Number of Nucleosomes (Number of Neighboring Nucleosomes)
**25 bp, dilute, low salt**	12	144
**30 bp, dilute, low salt**	11	132
**25 bp, dilute, high salt**	7	54
**30 bp, dilute, high salt**	5	51
**25 bp, condensed, high salt**	5 (75)	60 (900)
**30 bp, condensed, high salt**	5 (58)	60 (696)

Table S2: Role of histone tails in compacting 25 bp-like chromatin arrays as reported in the literature.

The studies here on long linker arrays are not uniformly consistent with each other, but generally show that all tails have been ascribed importance in array compaction by one or more authors. These results are consistent with our simulations (Fig. 2E-H), where for 25 bp arrays all tails make substantial inter-nucleosome contacts in high salt conditions, due to the configurational heterogeneity of the nucleosomes.

	Linker length (repeats*sequence)	Method (salt condition)	Important (Y/N)	Reference
H2A				
H2B				
H2A/H2B	61 bp (12*5 S rDNA)	Tailless, AUC (2 mM Mg^{2+})	Y	(*158*)
	61 bp (12*5 S rDNA)	Tailless, AUC (0-150 mM NaCl)	N	(*159*)
H3	61 bp (12*5 S rDNA)	Crosslinking (0-5 mM Mg^{2+})	Y	(*160*)
	61 bp (12*5 S rDNA)<br>61 bp (35*5 S rDNA)	Acetylation mimics, Crosslinking (0-5 mM Mg^{2+})	Y	(*161*)
H4	61 bp (12*5 S rDNA)	Crosslinking (Mg^{2+})	Y	(*157*)
	50 bp (12*601)	Tailless, Force spectroscopy	N	(*102*)
	61 bp (12*5 S rDNA)	Crosslinking (Mg^{2+})	Y	(*162*)
H3/H4	61 bp (12*5 S rDNA)	Tailless, AUC (2 mM Mg^{2+})	Y	(*158*)
	61 bp (12*5 S rDNA)	Tailless, AUC (0-150 mM NaCl)	Y	(*159*)

Table S3: Role of histone tails in compacting 30 bp-like chromatin arrays as reported in the literature.

A number of groups have studied nucleosome arrays with relatively short 10N linkers (30 bp or 20 bp), analogous to the 30 bp array used in our study. These have all found that H4 is important, consistent with the large number of inter-nucleosome contacts in our tail simulations (Fig. 2E-H). While other tails have not been extensively examined for short 10N arrays, only H2A has been found to contribute to compaction in the 30 bp system, while H2B and H3 have not. This is again consistent with our observations that after H4, H2A is the tail making the most inter-nucleosome contacts.

	Linker length (repeats*sequence)	Method (salt condition)	Important (Y/N)	Reference
H2A	30 bp (12*601)	Tailless, AUC (0.1-1 mM Mg^{2+})	N	(*101*)
	30 bp (12*601)	Mutation, AUC (0.8 mM Mg^{2+})	Y	(*163*)
H2B	30 bp (12*601)	Tailless, AUC (0.1-1 mM Mg^{2+})	N	(*101*)
H3	30 bp (12*601)	Tailless, AUC (0.1-1 mM Mg^{2+})	N	(*101*)
H4	30 bp (12*601)	Tailless, AUC (0.1-1 mM Mg^{2+})	Y	(*101*)
	30 bp (48*601)	Cross-linking, EM (1 mM Mg^{2+} , 50 mM K^{+})	Y	(*94*)
	30 bp (12*601)	Tailless, crosslinking (0 or 1 mM Mg^{2+})	Y	(*93*)
	30 bp (12*601)	Acetylation mimics, AUC (various salt)	Y	(*164*)
	20 bp (12*601)	Tailless, Force spectroscopy	Y	(*102*)

Supplementary Table 4: cryo-ET data collection and reconstruction statistics

	#1 25 bp chromatin (EMD-70745)	#2 30 bp chromatin (EMD-70743)
**Data collection and Processing (for each dataset)**		
Microscope	TFS Krios	TFS Krios
Voltage (keV)	300	300
Camera	Falcon 4i	Falcon 4i
Magnification	81000 x	81000 x
Pixel size at detector (Å/pixel)	1.516	1.516
Total electron exposure ($e^-/Å^2$)	150	150
Exposure rate (e-/pixel/sec)	10	10
Number of frames collected during exposure	251	251
Defocus range (µm)	3 - 5	3 - 5
Phase plate (if used)	N/A	N/A
- phase shift range (in degrees)	N/A	N/A
- number of images per phase plate position	N/A	N/A
Automation software (EPU, SerialEM or manual)	SerialEM	SerialEM
Tilt angle (if grid was tilted)	-40 to + 60	-40 to + 60
Energy filter slit width (if used)	5 eV	5 eV
Micrographs collected (no.)	~ 1000	~ 1000
Micrographs used (no.)	~ 500	~ 500
Total extracted particles (no.)	109,801	119,577
<u>**For each reconstruction:**</u>		
Refined particles (no.)	109,801	119,577
Final particles (no.)	104,438	111,909
Point-group or helical symmetry parameters	C1	C1
Estimated error of translations/rotations (if available)	N/A	N/A
Resolution (global, Å)	9.8/9.6	7.6/7.4
FSC 0.5 (unmasked/masked)	8.1/8.0	5.8/5.8
FSC 0.143 (unmasked/masked)	7-10.5	5.4-7.5
Resolution range (local, Å)		

Table S5: Role of histone tails in oligomerizing 25 bp-like chromatin arrays as reported in the literature.

Experimental data show that all tails contribute to chromatin oligomerization for long linkers and a 25 bp linker array, consistent with our simulations, where all tails make substantial intermolecular contacts in the condensed phase.

	Linker length (repeats*sequence)	Method (salt condition)	Important (Y/N)	Reference
H2A	61 bp (12*5 S rDNA)	Tailless, Precipitation (Mg^{2+})	Y	(*73*)
	61 bp (12*5 S rDNA) 60 bp (12*601)	Acetylation mimics, Precipitation (Mg^{2+})	Y/N	(*165*)
H2B	61 bp (12*5 S rDNA)	Tailless, Precipitation (Mg^{2+})	Y	(*73*)
	61 bp (12*5 S rDNA) 60 bp (12*601)	Acetylation mimics, Precipitation (Mg^{2+})	Y	(*165*)
H2A/H2B	61 bp (12*5 S rDNA)	Tailless, Precipitation (Mg^{2+})	Y	(*158*)
	61 bp (12*5 S rDNA)	Tailless, Precipitation (Mg^{2+})	Y	(*159*)
H3	61 bp (12*5 S rDNA)	Tailless, Precipitation (Mg^{2+})	Y	(*73*)
	61 bp (12*5 S rDNA) 60 bp (12*601)	Acetylation mimics, Precipitation (Mg^{2+})	Y	(*165*)
	61 bp (12*5 S rDNA) 60 bp (35*5 S rDNA)	Acetylation mimics, Precipitation (0-7 mM Mg^{2+})	Y	(*161*)
H4	61 bp (12*5 S rDNA)	Tailless, Precipitation (Mg^{2+})	Y	(*73*)
	61 bp (12*5 S rDNA)	Crosslinking, Precipitation (1-6 mM Mg^{2+})	Y	(*166*)
	61 bp (12*5 S rDNA) 60 bp (12*601)	Acetylation mimics, Precipitation (Mg^{2+})	Y	(*165*)
	61 bp (12*5 S rDNA) 60 bp (35*5 S rDNA)	Acetylation mimics, Precipitation (Mg^{2+})	Y	(*157*)
	25 bp (12*601)	Alanine mutations, Fluorescent imaging (150 mM KOAc)	Y	(*33*)
H3/H4	61 bp (12*5 S rDNA)	Tailless, Precipitation (Mg^{2+})	Y	(*158*)
	61 bp (12*5 S rDNA)	Tailless, Precipitation (Mg^{2+})	Y	(*159*)

Table S6: Role of histone tails in oligomerizing 30 bp-like chromatin arrays as reported in the literature.

Experimental data from several groups indicate that histone H4 tail mutations/truncation have the largest inhibitory effect on oligomerization of 30 bp chromatin. This likely arises because the H4 tail is needed both to maintain stacking in the 2-start helix (Supplementary Table 2, Figure 2I-K), and then also for intermolecular interactions when the helix is disrupted, as for arrays in Supplementary Table 3. Thus, the data are not easily interpretable in terms of oligomerization of wild type 30 bp chromatin, nor in comparing to our simulations, where the two-start helix is intact. Data on the other three histone tails is more ambiguous, with truncations in (*101*) showing no effect on chromatin precipitation, but acetylation mimicking K→Q mutations in (*165*) suggesting all tails play roles in precipitation. Our simulations would be more consistent with the latter behaviors, as H3, H2A(N) and H2B all make substantial intermolecular contacts in simulations of the 30 bp condensates.

	Linker length (repeats*sequence)	Method (salt condition)	Important (Y/N)	Reference
H2A	30 bp (12*601)	Tailless, Precipitation (0.1-1 mM Mg^{2+})	N	(*101*)
	30 bp (12*601)	Acetylation mimics, Precipitation (Mg^{2+})	Y/N	(*165*)
H2B	30 bp (12*601)	Tailless, Precipitation (0.1-1 mM Mg^{2+})	N	(*101*)
	30 bp (12*601)	Acetylation mimics, Precipitation (Mg^{2+})	Y	(*165*)
H3	30 bp (12*601)	Tailless, Precipitation (0.1-1 mM Mg^{2+})	N	(*101*)
	30 bp (12*601)	Acetylation mimics, Precipitation (Mg^{2+})	Y	(*165*)
H4	30 bp (12*601)	Tailless, Precipitation (0.1-1 mM Mg^{2+})	Y	(*101*)
	30 bp (12*601)	Crosslinking, Precipitation (1-6 mM Mg^{2+})	Y	(*166*)
	30 bp (12*601)	Acetylation mimics, Precipitation (various salt)	Y	(*164*)
	30 bp (12*601)	Acetylation mimics, Precipitation (Mg^{2+})	Y	(*165*)

Table S7. Statistical comparison of model selection for video particle tracking microrheology.

An F-test was performed to determine whether the Jeffery or Maxwell model provides a better fit for calculating the viscoelastic moduli of 25 bp and 30 bp chromatin. For both chromatin systems, the Jeffery model generally showed a better fit. In the case of 25 bp chromatin, the F value is 10, which exceeds the critical value of the F-distribution (6.944), indicating that the Jeffery model is preferred. For 30 bp chromatin, the F value is 1.6, which does not exceed the critical threshold, suggesting that the Maxwell model is more appropriate.

	p-value	F	Critical value of the F-Distribution (α=0.05)
25 bp chromatin	≈0.00	10	6.944
30 bp chromatin	1.79e-135	1.6	6.944

Movie S1. Tracing of nucleosome arrays in low salt dilute phase for 25 bp chromatin condensates. Movie shows slices through a tomogram of chromatin in non-phase separating conditions, and traces of selected nucleosome arrays.

Movie S2. Tracing of nucleosome arrays in low salt dilute phase for 30 bp chromatin condensates. Movie shows slices through a tomogram of chromatin in non-phase separating conditions, and traces of selected nucleosome arrays.

Movie S3. Tomogram denoising, segmentation and nucleosome assignment for chromatin condensates. Movie shows a slice through a chromatin condensate during the different stages of image processing.

Movie S4. Tracing of nucleosome arrays in high salt condensed phase for 25 bp chromatin condensates. Movie shows slices through a tomogram of a 25 bp chromatin condensate, and traces of selected nucleosome arrays.

Movie S5. Tracing of nucleosome arrays in high salt condensed phase for 30 bp chromatin condensates. Movie shows slices through a tomogram of a 30 bp chromatin condensates, and traces of selected nucleosome arrays.

Movie S6. Overview of multi-scale structural studies on chromatin condensates, spanning histone tail interactions, molecular interactions, and mesoscale condensate properties.

Movie S7. Representative fluorescence microscopy recordings used for particle tracking microrheology in 25 bp and 30 bp chromatin condensates. Fluorescent beads are green and condensate is red.

Movie S8. Single-molecule trajectories within 25 bp and 30 bp chromatin condensates.

References and Notes

1. P. Li, S. Banjade, H.-C. Cheng, S. Kim, B. Chen, L. Guo, M. Llaguno, J. V. Hollingsworth, D. S. King, S. F. Banani, P. S. Russo, Q.-X. Jiang, B. T. Nixon, M. K. Rosen, Phase transitions in the assembly of multivalent signalling proteins. *Nature* **483**, 336–340 (2012).

2. S. F. Banani, H. O. Lee, A. A. Hyman, M. K. Rosen, Biomolecular condensates: organizers of cellular biochemistry. *Nature Reviews Molecular Cell Biology* **18**, 285–298 (2017).

3. Y. Shin, C. P. Brangwynne, Liquid phase condensation in cell physiology and disease. *Science* **357**, eaaf4382 (2017).

4. I. Alshareedah, W. M. Borcherds, S. R. Cohen, A. Singh, A. E. Posey, M. Farag, A. Bremer, G. W. Strout, D. T. Tomares, R. V. Pappu, T. Mittag, P. R. Banerjee, Sequence-specific interactions determine viscoelasticity and ageing dynamics of protein condensates. *Nat. Phys.*, doi: 10.1038/s41567-024-02558-1 (2024).

5. S. R. Cohen, P. R. Banerjee, R. V. Pappu, Direct computations of viscoelastic moduli of biomolecular condensates. *The Journal of Chemical Physics* **161**, 095103 (2024).

6. A. Chandrasekaran, K. Graham, J. C. Stachowiak, P. Rangamani, Kinetic trapping organizes actin filaments within liquid-like protein droplets. *Nat Commun* **15**, 3139 (2024).

7. A. S. Lyon, W. B. Peeples, M. K. Rosen, A framework for understanding the functions of biomolecular condensates across scales. *Nat Rev Mol Cell Biol* **22**, 215–235 (2021).

8. D. L. J. Lafontaine, J. A. Riback, R. Bascetin, C. P. Brangwynne, The nucleolus as a multiphase liquid condensate. *Nat Rev Mol Cell Biol* **22**, 165–182 (2021).

9. A. Yamasaki, J. Md. Alam, D. Noshiro, E. Hirata, Y. Fujioka, K. Suzuki, Y. Ohsumi, N. N. Noda, Liquidity Is a Critical Determinant for Selective Autophagy of Protein Condensates. *Molecular Cell* **77**, 1163-1175.e9 (2020).

10. Z. Wang, D. Chen, D. Guan, X. Liang, J. Xue, H. Zhao, G. Song, J. Lou, Y. He, H. Zhang, Material properties of phase-separated TFEB condensates regulate the autophagy-lysosome pathway. *Journal of Cell Biology* **221**, e202112024 (2022).

11. P. Guo, B. Li, W. Dong, H. Zhou, L. Wang, T. Su, C. Carl, Y. Zheng, Y. Hong, H. Deng, D. Pan, PI4P-mediated solid-like Merlin condensates orchestrate Hippo pathway regulation. *Science* **385**, eadf4478 (2024).

12. J. Risso-Ballester, M. Galloux, J. Cao, R. Le Goffic, F. Hontonnou, A. Jobart-Malfait, A. Desquesnes, S. M. Sake, S. Haid, M. Du, X. Zhang, H. Zhang, Z. Wang, V. Rincheval, Y. Zhang, T. Pietschmann, J.-F. Eléouët, M.-A. Rameix-Welti, R. Altmeyer, A condensate-hardening drug blocks RSV replication in vivo. *Nature* **595**, 596–599 (2021).

13. L.-P. Bergeron-Sandoval, S. Kumar, H. K. Heris, C. L. A. Chang, C. E. Cornell, S. L. Keller, P. François, A. G. Hendricks, A. J. Ehrlicher, R. V. Pappu, S. W. Michnick, Endocytic proteins with prion-like domains form viscoelastic condensates that enable membrane remodeling. *Proc. Natl. Acad. Sci. U.S.A.* **118**, e2113789118 (2021).

14. S. Ambadi Thody, H. D. Clements, H. Baniasadi, A. S. Lyon, M. S. Sigman, M. K. Rosen, Small-molecule properties define partitioning into biomolecular condensates. *Nat. Chem.*, doi: 10.1038/s41557-024-01630-w (2024).

15. M. R. King, K. M. Ruff, A. Z. Lin, A. Pant, M. Farag, J. M. Lalmansingh, T. Wu, M. J. Fossat, W. Ouyang, M. D. Lew, E. Lundberg, M. D. Vahey, R. V. Pappu, Macromolecular condensation organizes nucleolar sub-phases to set up a pH gradient. *Cell* **187**, 1889-1906.e24 (2024).

16. A. E. Posey, A. Bremer, N. A. Erkamp, A. Pant, T. P. J. Knowles, Y. Dai, T. Mittag, R. V. Pappu, Biomolecular Condensates are Characterized by Interphase Electric Potentials. *J. Am. Chem. Soc.*, jacs.4c08946 (2024).

17. E. W. Martin, C. Iserman, B. Olety, D. M. Mitrea, I. A. Klein, Biomolecular Condensates as Novel Antiviral Targets. *Journal of Molecular Biology* **436**, 168380 (2024).

18. D. M. Mitrea, M. Mittasch, B. F. Gomes, I. A. Klein, M. A. Murcko, Modulating biomolecular condensates: a novel approach to drug discovery. *Nat Rev Drug Discov* **21**, 841–862 (2022).

19. T. H. Kim, B. Tsang, R. M. Vernon, N. Sonenberg, L. E. Kay, J. D. Forman-Kay, Phospho-dependent phase separation of FMRP and CAPRIN1 recapitulates regulation of translation and deadenylation. *Science* **365**, 825–829 (2019).

20. E. W. Martin, A. S. Holehouse, I. Peran, M. Farag, J. J. Incicco, A. Bremer, C. R. Grace, A. Soranno, R. V. Pappu, T. Mittag, Valence and patterning of aromatic residues determine the phase behavior of prion-like domains.

21. A. C. Murthy, G. L. Dignon, Y. Kan, G. H. Zerze, S. H. Parekh, J. Mittal, N. L. Fawzi, Molecular interactions underlying liquid−liquid phase separation of the FUS low-complexity domain. *Nat Struct Mol Biol* **26**, 637–648 (2019).

22. M. Bose, M. Lampe, J. Mahamid, A. Ephrussi, Liquid-to-solid phase transition of oskar ribonucleoprotein granules is essential for their function in Drosophila embryonic development. *Cell* **185**, 1308-1324.e23 (2022).

23. M. Zhang, C. Díaz-Celis, B. Onoa, C. Cañari-Chumpitaz, K. I. Requejo, J. Liu, M. Vien, E. Nogales, G. Ren, C. Bustamante, Molecular organization of the early stages of nucleosome phase separation visualized by cryo-electron tomography. *Molecular Cell*, S1097276522006505 (2022).

24. J. Guillén-Boixet, A. Kopach, A. S. Holehouse, S. Wittmann, M. Jahnel, R. Schlüßler, K. Kim, I. R. E. A. Trussina, J. Wang, D. Mateju, I. Poser, S. Maharana, M. Ruer-Gruß, D. Richter, X. Zhang, Y.-T. Chang, J. Guck, A. Honigmann, J. Mahamid, A. A. Hyman, R.

V. Pappu, S. Alberti, T. M. Franzmann, RNA-Induced Conformational Switching and Clustering of G3BP Drive Stress Granule Assembly by Condensation. *Cell* **181**, 346-361.e17 (2020).

25. F. Tollervey, X. Zhang, M. Bose, J. Sachweh, J. B. Woodruff, T. M. Franzmann, J. Mahamid, "Cryo-Electron Tomography of Reconstituted Biomolecular Condensates" in *Phase-Separated Biomolecular Condensates: Methods and Protocols*, H.-X. Zhou, J.-H. Spille, P. R. Banerjee, Eds. (Springer US, New York, NY, 2023; https://doi.org/10.1007/978-1-0716-2663-4_15), pp. 297–324.

26. X. Zhang, S. Sridharan, I. Zagoriy, C. Eugster Oegema, C. Ching, T. Pflaesterer, H. K. H. Fung, I. Becher, I. Poser, C. W. Müller, A. A. Hyman, M. M. Savitski, J. Mahamid, Molecular mechanisms of stress-induced reactivation in mumps virus condensates. *Cell* **186**, 1877-1894.e27 (2023).

27. J. B. Woodruff, B. Ferreira Gomes, P. O. Widlund, J. Mahamid, A. Honigmann, A. A. Hyman, The Centrosome Is a Selective Condensate that Nucleates Microtubules by Concentrating Tubulin. *Cell* **169**, 1066-1077.e10 (2017).

28. F. J. B. Bäuerlein, I. Saha, A. Mishra, M. Kalemanov, A. Martínez-Sánchez, R. Klein, I. Dudanova, M. S. Hipp, F. U. Hartl, W. Baumeister, R. Fernández-Busnadiego, In Situ Architecture and Cellular Interactions of PolyQ Inclusions. *Cell* **171**, 179-187.e10 (2017).

29. X. Liu, X. Xia, M. W. Martynowycz, T. Gonen, Z. H. Zhou, Molecular sociology of virus-induced cellular condensates supporting reovirus assembly and replication. *Nat Commun* **15**, 10638 (2024).

30. A. R. Tejedor, R. Collepardo-Guevara, J. Ramírez, J. R. Espinosa, Time-Dependent Material Properties of Aging Biomolecular Condensates from Different Viscoelasticity Measurements in Molecular Dynamics Simulations. *J. Phys. Chem. B* **127**, 4441–4459 (2023).

31. A. R. Tejedor, I. Sanchez-Burgos, M. Estevez-Espinosa, A. Garaizar, R. Collepardo-Guevara, J. Ramirez, J. R. Espinosa, Protein structural transitions critically transform the network connectivity and viscoelasticity of RNA-binding protein condensates but RNA can prevent it. *Nat Commun* **13**, 5717 (2022).

32. I. Sanchez-Burgos, J. A. Joseph, R. Collepardo-Guevara, J. R. Espinosa, Size conservation emerges spontaneously in biomolecular condensates formed by scaffolds and surfactant clients. *Sci Rep* **11**, 15241 (2021).

33. B. A. Gibson, L. K. Doolittle, M. W. G. Schneider, L. E. Jensen, N. Gamarra, L. Henry, D. W. Gerlich, S. Redding, M. K. Rosen, Organization of Chromatin by Intrinsic and Regulated Phase Separation. *Cell* **179**, 470-484.e21 (2019).

34. M. W. G. Schneider, B. A. Gibson, S. Otsuka, M. F. D. Spicer, M. Petrovic, C. Blaukopf, C. C. H. Langer, P. Batty, T. Nagaraju, L. K. Doolittle, M. K. Rosen, D. W. Gerlich, A mitotic chromatin phase transition prevents perforation by microtubules. *Nature*, doi: 10.1038/s41586-022-05027-y (2022).

35. B. A. Gibson, C. Blaukopf, T. Lou, L. Chen, L. K. Doolittle, I. Finkelstein, G. J. Narlikar, D. W. Gerlich, M. K. Rosen, In diverse conditions, intrinsic chromatin condensates have liquid-like material properties. *Proc. Natl. Acad. Sci. U.S.A.* **120**, e2218085120 (2023).

36. M. Lakadamyali, M. P. Cosma, Visualizing the genome in high resolution challenges our textbook understanding. *Nat Methods* **17**, 371–379 (2020).

37. A. G. Larson, D. Elnatan, M. M. Keenen, M. J. Trnka, J. B. Johnston, A. L. Burlingame, D. A. Agard, S. Redding, G. J. Narlikar, Liquid droplet formation by HP1α suggests a role for phase separation in heterochromatin. *Nature* **547**, 236–240 (2017).

38. A. R. Strom, A. V. Emelyanov, M. Mir, D. V. Fyodorov, X. Darzacq, G. H. Karpen, Phase separation drives heterochromatin domain formation. *Nature* **547**, 241–245 (2017).

39. I. Solovei, M. Kreysing, C. Lanctôt, S. Kösem, L. Peichl, T. Cremer, J. Guck, B. Joffe, Nuclear Architecture of Rod Photoreceptor Cells Adapts to Vision in Mammalian Evolution. *Cell* **137**, 356–368 (2009).

40. H. Belaghzal, T. Borrman, A. D. Stephens, D. L. Lafontaine, S. V. Venev, Z. Weng, J. F. Marko, J. Dekker, Liquid chromatin Hi-C characterizes compartment-dependent chromatin interaction dynamics. *Nat Genet* **53**, 367–378 (2021).

41. J. Dekker, L. A. Mirny, The chromosome folding problem and how cells solve it. *Cell* **187**, 6424–6450 (2024).

42. R. R. Cheng, V. G. Contessoto, E. Lieberman Aiden, P. G. Wolynes, M. Di Pierro, J. N. Onuchic, Exploring chromosomal structural heterogeneity across multiple cell lines. *eLife* **9**, e60312 (2020).

43. Y. Itoh, E. J. Woods, K. Minami, K. Maeshima, R. Collepardo-Guevara, Liquid-like chromatin in the cell: What can we learn from imaging and computational modeling? *Current Opinion in Structural Biology* **71**, 123–135 (2021).

44. K. Luger, A. W. Mäder, R. K. Richmond, D. F. Sargent, T. J. Richmond, Crystal structure of the nucleosome core particle at 2.8 Å resolution. *Nature* **389**, 251–260 (1997).

45. S. Pepenella, K. J. Murphy, J. J. Hayes, Intra- and inter-nucleosome interactions of the core histone tail domains in higher-order chromatin structure. *Chromosoma* **123**, 3–13 (2014).

46. M. A. Ricci, C. Manzo, M. F. García-Parajo, M. Lakadamyali, M. P. Cosma, Chromatin Fibers Are Formed by Heterogeneous Groups of Nucleosomes In Vivo. *Cell* **160**, 1145–1158 (2015).

47. M. Ohno, T. Ando, D. G. Priest, V. Kumar, Y. Yoshida, Y. Taniguchi, Sub-nucleosomal Genome Structure Reveals Distinct Nucleosome Folding Motifs. *Cell* **176**, 520-534.e25 (2019).

48. H. D. Ou, S. Phan, T. J. Deerinck, A. Thor, M. H. Ellisman, C. C. O'Shea, ChromEMT: Visualizing 3D chromatin structure and compaction in interphase and mitotic cells. *Science* **357**, eaag0025 (2017).

49. T. Nozaki, R. Imai, M. Tanbo, R. Nagashima, S. Tamura, T. Tani, Y. Joti, M. Tomita, K. Hibino, M. T. Kanemaki, K. S. Wendt, Y. Okada, T. Nagai, K. Maeshima, Dynamic Organization of Chromatin Domains Revealed by Super-Resolution Live-Cell Imaging. *Molecular Cell* **67**, 282-293.e7 (2017).

50. E. Miron, R. Oldenkamp, J. M. Brown, D. M. S. Pinto, C. S. Xu, A. R. Faria, H. A. Shaban, J. D. P. Rhodes, C. Innocent, S. de Ornellas, H. F. Hess, V. Buckle, L. Schermelleh, Chromatin arranges in chains of mesoscale domains with nanoscale functional topography independent of cohesin. *Science Advances* **6**, eaba8811 (2020).

51. G. Pei, H. Lyons, P. Li, B. R. Sabari, Transcription regulation by biomolecular condensates. *Nat Rev Mol Cell Biol*, doi: 10.1038/s41580-024-00789-x (2024).

52. S. Brahmachari, S. Tripathi, J. N. Onuchic, H. Levine, Nucleosomes play a dual role in regulating transcription dynamics. *Proc. Natl. Acad. Sci. U.S.A.* **121**, e2319772121 (2024).

53. S. Sekine, H. Ehara, T. Kujirai, H. Kurumizaka, Structural perspectives on transcription in chromatin. *Trends in Cell Biology*, S0962892423001551 (2023).

54. A. E. Ehrenhofer-Murray, Chromatin dynamics at DNA replication, transcription and repair. *European Journal of Biochemistry* **271**, 2335–2349 (2004).

55. E. F. Hammonds, M. C. Harwig, E. A. Paintsil, E. A. Tillison, R. B. Hill, E. A. Morrison, Histone H3 and H4 tails play an important role in nucleosome phase separation. *Biophysical Chemistry* **283**, 106767 (2022).

56. X. Li, Z. An, W. Zhang, F. Li, Phase Separation: Direct and Indirect Driving Force for High-Order Chromatin Organization. *Genes* **14**, 499 (2023).

57. S. Sanulli, M. J. Trnka, V. Dharmarajan, R. W. Tibble, B. D. Pascal, A. L. Burlingame, P. R. Griffin, J. D. Gross, G. J. Narlikar, HP1 reshapes nucleosome core to promote phase separation of heterochromatin. *Nature* **575**, 390–394 (2019).

58. H. Strickfaden, T. O. Tolsma, A. Sharma, D. A. Underhill, J. C. Hansen, M. J. Hendzel, Condensed Chromatin Behaves like a Solid on the Mesoscale In Vitro and in Living Cells. *Cell* **183**, 1772-1784.e13 (2020).

59. A. Shakya, S. Park, N. Rana, J. T. King, Liquid-Liquid Phase Separation of Histone Proteins in Cells: Role in Chromatin Organization. *Biophysical Journal* **118**, 753–764 (2020).

60. Q. Chen, L. Zhao, A. Soman, A. Y. Arkhipova, J. Li, H. Li, Y. Chen, X. Shi, L. Nordenskiöld, Chromatin Liquid–Liquid Phase Separation (LLPS) Is Regulated by Ionic Conditions and Fiber Length. *Cells* **11**, 3145 (2022).

61. T. Nozaki, S. Shinkai, S. Ide, K. Higashi, S. Tamura, M. A. Shimazoe, M. Nakagawa, Y. Suzuki, Y. Okada, M. Sasai, S. Onami, K. Kurokawa, S. Iida, K. Maeshima, Condensed but liquid-like domain organization of active chromatin regions in living human cells. *Sci. Adv.* **9**, eadf1488 (2023).

62. Y. Li, H. Zhang, X. Li, W. Wu, P. Zhu, Cryo-ET study from in vitro to in vivo revealed a general folding mode of chromatin with two-start helical architecture. *Cell Reports* **42**, 113134 (2023).

63. A. J. Beel, M. Azubel, P.-J. Matteï, R. D. Kornberg, Structure of mitotic chromosomes. *Molecular Cell*, S1097276521006870 (2021).

64. F. Fatmaoui, P. Carrivain, D. Grewe, B. Jakob, J.-M. Victor, A. Leforestier, M. Eltsov, Cryo-electron tomography and deep learning denoising reveal native chromatin landscapes of interphase nuclei. *bioRxiv*, 2022.08.16.502515 (2022).

65. Z. Hou, F. Nightingale, Y. Zhu, C. MacGregor-Chatwin, P. Zhang, Structure of native chromatin fibres revealed by Cryo-ET in situ. *Nat Commun* **14**, 6324 (2023).

66. B. Bintu, L. J. Mateo, J.-H. Su, N. A. Sinnott-Armstrong, M. Parker, S. Kinrot, K. Yamaya, A. N. Boettiger, X. Zhuang, Super-resolution chromatin tracing reveals domains and cooperative interactions in single cells. *Science* **362**, eaau1783 (2018).

67. T.-H. S. Hsieh, A. Weiner, B. Lajoie, J. Dekker, N. Friedman, O. J. Rando, Mapping Nucleosome Resolution Chromosome Folding in Yeast by Micro-C. *Cell* **162**, 108–119 (2015).

68. V. I. Risca, S. K. Denny, A. F. Straight, W. J. Greenleaf, Variable chromatin structure revealed by in situ spatially correlated DNA cleavage mapping. *Nature* **541**, 237–241 (2017).

69. S. Cai, D. Böck, M. Pilhofer, L. Gan, The in situ structures of mono-, di-, and trinucleosomes in human heterochromatin. *MBoC* **29**, 2450–2457 (2018).

70. J. K. Chen, T. Liu, S. Cai, W. Ruan, C. T. Ng, J. Shi, U. Surana, L. Gan, Nanoscale analysis of human G1 and metaphase chromatin in situ. *EMBO J* **44**, 2658–2694 (2025).

71. P. T. Lowary, J. Widom, New DNA sequence rules for high affinity binding to histone octamer and sequence-directed nucleosome positioning. *Journal of Molecular Biology* **276**, 19–42 (1998).

72. A. Flaus, Principles and practice of nucleosome positioning *in vitro*. *Frontiers in Life Science* **5**, 5–27 (2011).

73. F. Gordon, K. Luger, J. C. Hansen, The Core Histone N-terminal Tail Domains Function Independently and Additively during Salt-dependent Oligomerization of Nucleosomal Arrays. *Journal of Biological Chemistry* **280**, 33701–33706 (2005).

74. K. Maeshima, R. Rogge, S. Tamura, Y. Joti, T. Hikima, H. Szerlong, C. Krause, J. Herman, E. Seidel, J. DeLuca, T. Ishikawa, J. C. Hansen, Nucleosomal arrays self-assemble into supramolecular globular structures lacking 30-nm fibers. *EMBO J* **35**, 1115–1132 (2016).

75. J. C. Hansen, K. Maeshima, M. J. Hendzel, The solid and liquid states of chromatin. *Epigenetics & Chromatin* **14**, 50 (2021).

76. A. Allahverdi, Q. Chen, N. Korolev, L. Nordenskiöld, Chromatin compaction under mixed salt conditions: Opposite effects of sodium and potassium ions on nucleosome array folding. *Sci Rep* **5**, 8512 (2015).

77. N. Korolev, A. Allahverdi, Y. Yang, Y. Fan, A. P. Lyubartsev, L. Nordenskiöld, Electrostatic Origin of Salt-Induced Nucleosome Array Compaction. *Biophysical Journal* **99**, 1896–1905 (2010).

78. M. Kruithof, F.-T. Chien, A. Routh, C. Logie, D. Rhodes, J. Van Noort, Single-molecule force spectroscopy reveals a highly compliant helical folding for the 30-nm chromatin fiber. *Nat Struct Mol Biol* **16**, 534–540 (2009).

79. J. Widom, Physicochemical studies of the folding of the 100 Å nucleosome filament into the 300 Å filament. *Journal of Molecular Biology* **190**, 411–424 (1986).

80. N. Jentink, C. Purnell, B. Kable, M. T. Swulius, S. A. Grigoryev, Cryoelectron tomography reveals the multiplex anatomy of condensed native chromatin and its unfolding by histone citrullination. *Molecular Cell* **83**, 3236-3252.e7 (2023).

81. M. Zhang, C. Díaz-Celis, J. Liu, J. Tao, P. D. Ashby, C. Bustamante, G. Ren, Angle between DNA linker and nucleosome core particle regulates array compaction revealed by individual-particle cryo-electron tomography. *Nat Commun* **15**, 4395 (2024).

82. T. Nikitina, D. Norouzi, S. A. Grigoryev, V. B. Zhurkin, DNA topology in chromatin is defined by nucleosome spacing. *Science Advances* **3**, e1700957 (2017).

83. L. Chen, M. J. Maristany, S. E. Farr, J. Luo, B. A. Gibson, L. K. Doolittle, J. R. Espinosa, J. Huertas, S. Redding, R. Collepardo-Guevara, M. K. Rosen, Nucleosome spacing can fine-tune higher-order chromatin assembly. *Nature Communications* **16**, 6315 (2025).

84. H. Kenzaki, S. Takada, Linker DNA Length is a Key to Tri-nucleosome Folding. *Journal of Molecular Biology* **433**, 166792 (2021).

85. T. Schalch, S. Duda, D. F. Sargent, T. J. Richmond, X-ray structure of a tetranucleosome and its implications for the chromatin fibre. *Nature* **436**, 138–141 (2005).

86. F. Song, P. Chen, D. Sun, M. Wang, L. Dong, D. Liang, R.-M. Xu, P. Zhu, G. Li, Cryo-EM Study of the Chromatin Fiber Reveals a Double Helix Twisted by Tetranucleosomal Units. *Science* **344**, 376–380 (2014).

87. A. Golembeski, J. Lequieu, A Molecular View into the Structure and Dynamics of Phase-Separated Chromatin. *J. Phys. Chem. B* **128**, 10593–10603 (2024).

88. S. E. Farr, E. J. Woods, J. A. Joseph, A. Garaizar, R. Collepardo-Guevara, Nucleosome plasticity is a critical element of chromatin liquid–liquid phase separation and multivalent nucleosome interactions. *Nat Commun* **12**, 2883 (2021).

89. G. Arya, T. Schlick, A Tale of Tails: How Histone Tails Mediate Chromatin Compaction in Different Salt and Linker Histone Environments. *J. Phys. Chem. A* **113**, 4045–4059 (2009).

90. Y. Qiu, S. Liu, X. Lin, I. C. Unarta, X. Huang, B. Zhang, Nucleosome condensate and linker DNA alter chromatin folding pathways and rates. *bioRxiv*, 2024.11.15.623891 (2024).

91. P. J. J. Robinson, W. An, A. Routh, F. Martino, L. Chapman, R. G. Roeder, D. Rhodes, 30 nm Chromatin Fibre Decompaction Requires both H4-K16 Acetylation and Linker Histone Eviction. *Journal of Molecular Biology* **381**, 816–825 (2008).

92. R. Collepardo-Guevara, G. Portella, M. Vendruscolo, D. Frenkel, T. Schlick, M. Orozco, Chromatin Unfolding by Epigenetic Modifications Explained by Dramatic Impairment of Internucleosome Interactions: A Multiscale Computational Study. *J. Am. Chem. Soc.* **137**, 10205–10215 (2015).

93. M. Shogren-Knaak, H. Ishii, J.-M. Sun, M. J. Pazin, J. R. Davie, C. L. Peterson, Histone H4-K16 Acetylation Controls Chromatin Structure and Protein Interactions. *Science* **311**, 844–847 (2006).

94. B. Dorigo, T. Schalch, A. Kulangara, S. Duda, R. R. Schroeder, T. J. Richmond, Nucleosome Arrays Reveal the Two-Start Organization of the Chromatin Fiber. *Science* **306**, 1571–1573 (2004).

95. J. Zhou, J. Y. Fan, D. Rangasamy, D. J. Tremethick, The nucleosome surface regulates chromatin compaction and couples it with transcriptional repression. *Nat Struct Mol Biol* **14**, 1070–1076 (2007).

96. Q. Chen, R. Yang, N. Korolev, C. F. Liu, L. Nordenskiöld, Regulation of Nucleosome Stacking and Chromatin Compaction by the Histone H4 N-Terminal Tail–H2A Acidic Patch Interaction. *Journal of Molecular Biology* **429**, 2075–2092 (2017).

97. T. S. Lewis, V. Sokolova, H. Jung, H. Ng, D. Tan, Structural basis of chromatin regulation by histone variant H2A.Z. *Nucleic Acids Research* **49**, 11379–11391 (2021).

98. W. Alvarado, J. Moller, A. L. Ferguson, J. J. De Pablo, Tetranucleosome Interactions Drive Chromatin Folding. *ACS Cent. Sci.* **7**, 1019–1027 (2021).

99. R. Collepardo-Guevara, T. Schlick, Chromatin fiber polymorphism triggered by variations of DNA linker lengths. *Proc. Natl. Acad. Sci. U.S.A.* **111**, 8061–8066 (2014).

100. O. Perišić, R. Collepardo-Guevara, T. Schlick, Modeling Studies of Chromatin Fiber Structure as a Function of DNA Linker Length. *Journal of Molecular Biology* **403**, 777–802 (2010).

101. B. Dorigo, T. Schalch, K. Bystricky, T. J. Richmond, Chromatin Fiber Folding: Requirement for the Histone H4 N-terminal Tail. *Journal of Molecular Biology* **327**, 85–96 (2003).

102. T. Brouwer, C. Pham, A. Kaczmarczyk, W.-J. de Voogd, M. Botto, P. Vizjak, F. Mueller-Planitz, J. van Noort, A critical role for linker DNA in higher-order folding of chromatin fibers. *Nucleic Acids Research* **49**, 2537–2551 (2021).

103. H. Zhou, J. Hutchings, M. Shiozaki, X. Zhao, L. K. Doolittle, S. Yang, R. Yan, N. Jean, M. Riggi, Z. Yu, E. Villa, M. K. Rosen, Quantitative spatial analysis of chromatin biomolecular condensates using cryoelectron tomography. *Proceedings of the National Academy of Sciences* **122**, e2426449122 (2025).

104. S. Bilokapic, M. Strauss, M. Halic, Histone octamer rearranges to adapt to DNA unwrapping. *Nat Struct Mol Biol* **25**, 101–108 (2018).

105. Y. Takizawa, C.-H. Ho, H. Tachiwana, H. Matsunami, W. Kobayashi, M. Suzuki, Y. Arimura, T. Hori, T. Fukagawa, M. D. Ohi, M. Wolf, H. Kurumizaka, Cryo-EM Structures of Centromeric Tri-nucleosomes Containing a Central CENP-A Nucleosome. *Structure* **28**, 44-53.e4 (2020).

106. M. Dombrowski, M. Engeholm, C. Dienemann, S. Dodonova, P. Cramer, Histone H1 binding to nucleosome arrays depends on linker DNA length and trajectory. *Nat Struct Mol Biol* **29**, 493–501 (2022).

107. H.-X. Zhou, D. Kota, S. Qin, R. Prasad, Fundamental Aspects of Phase-Separated Biomolecular Condensates. *Chem. Rev.* **124**, 8550–8595 (2024).

108. D. Kota, R. Prasad, H.-X. Zhou, Adenosine Triphosphate Mediates Phase Separation of Disordered Basic Proteins by Bridging Intermolecular Interaction Networks. *J. Am. Chem. Soc.* **146**, 1326–1336 (2024).

109. S. Iida, S. Shinkai, Y. Itoh, S. Tamura, M. T. Kanemaki, S. Onami, K. Maeshima, Single-nucleosome imaging reveals steady-state motion of interphase chromatin in living human cells. *Sci. Adv.* **8**, eabn5626 (2022).

110. I. Y. Wong, M. L. Gardel, D. R. Reichman, E. R. Weeks, M. T. Valentine, A. R. Bausch, D. A. Weitz, Anomalous Diffusion Probes Microstructure Dynamics of Entangled F-Actin Networks. *Phys. Rev. Lett.* **92**, 178101 (2004).

111. L.-H. Cai, S. Panyukov, M. Rubinstein, Mobility of Nonsticky Nanoparticles in Polymer Liquids. *Macromolecules* **44**, 7853–7863 (2011).

112. L.-H. Cai, S. Panyukov, M. Rubinstein, Hopping Diffusion of Nanoparticles in Polymer Matrices. *Macromolecules* **48**, 847–862 (2015).

113. M. Shayegan, R. Tahvildari, K. Metera, L. Kisley, S. W. Michnick, S. R. Leslie, Probing Inhomogeneous Diffusion in the Microenvironments of Phase-Separated Polymers under Confinement. *J. Am. Chem. Soc.* **141**, 7751–7757 (2019).

114. M. L. Gardel, K. E. Kasza, C. P. Brangwynne, J. Liu, D. A. Weitz, "Chapter 19 Mechanical Response of Cytoskeletal Networks" in *Methods in Cell Biology* (Elsevier, 2008; https://linkinghub.elsevier.com/retrieve/pii/S0091679X08006195)vol. 89, pp. 487–519.

115. B. E. Vos, T. M. Muenker, T. Betz, Characterizing intracellular mechanics via optical tweezers-based microrheology. *Current Opinion in Cell Biology* **88**, 102374 (2024).

116. M. Lee, H. C. Moon, H. Jeong, D. W. Kim, H. Y. Park, Y. Shin, Optogenetic control of mRNA condensation reveals an intimate link between condensate material properties and functions. *Nat Commun* **15**, 3216 (2024).

117. Y. Dai, Z. Zhou, W. Yu, Y. Ma, K. Kim, N. Rivera, J. Mohammed, E. Lantelme, H. Hsu-Kim, A. Chilkoti, L. You, Biomolecular condensates regulate cellular electrochemical equilibria. *Cell*, S0092867424009097 (2024).

118. T. Wu, M. R. King, Y. Qiu, M. Farag, R. V. Pappu, M. D. Lew, Single fluorogen imaging reveals distinct environmental and structural features of biomolecular condensates. Biophysics [Preprint] (2023). https://doi.org/10.1101/2023.01.26.525727.

119. C. Mathieu, R. V. Pappu, J. P. Taylor, Beyond aggregation: Pathological phase transitions in neurodegenerative disease. *Science* **370**, 56–60 (2020).

120. P. J. J. Robinson, L. Fairall, V. A. T. Huynh, D. Rhodes, EM measurements define the dimensions of the "30-nm" chromatin fiber: Evidence for a compact, interdigitated structure. *Proc. Natl. Acad. Sci. U.S.A.* **103**, 6506–6511 (2006).

121. E. M. Hildebrand, J. Dekker, Mechanisms and Functions of Chromosome Compartmentalization. *Trends in Biochemical Sciences* **45**, 385–396 (2020).

122. Q. Szabo, A. Donjon, I. Jerković, G. L. Papadopoulos, T. Cheutin, B. Bonev, E. P. Nora, B. G. Bruneau, F. Bantignies, G. Cavalli, Regulation of single-cell genome organization into TADs and chromatin nanodomains. *Nat Genet* **52**, 1151–1157 (2020).

123. J. Xu, X. Xu, D. Huang, Y. Luo, L. Lin, X. Bai, Y. Zheng, Q. Yang, Y. Cheng, A. Huang, J. Shi, X. Bo, J. Gu, H. Chen, A comprehensive benchmarking with interpretation and operational guidance for the hierarchy of topologically associating domains. *Nat Commun* **15**, 4376 (2024).

124. M. Hu, S. Wang, Chromatin Tracing: Imaging 3D Genome and Nucleome. *Trends in Cell Biology* **31**, 5–8 (2021).

125. S. Wang, J.-H. Su, B. J. Beliveau, B. Bintu, J. R. Moffitt, C. Wu, X. Zhuang, Spatial organization of chromatin domains and compartments in single chromosomes. *Science* **353**, 598–602 (2016).

126. L. Tan, D. Xing, C.-H. Chang, H. Li, X. S. Xie, Three-dimensional genome structures of single diploid human cells. *Science* **361**, 924–928 (2018).

127. T. J. Stevens, D. Lando, S. Basu, L. P. Atkinson, Y. Cao, S. F. Lee, M. Leeb, K. J. Wohlfahrt, W. Boucher, A. O'Shaughnessy-Kirwan, J. Cramard, A. J. Faure, M. Ralser, E. Blanco, L. Morey, M. Sansó, M. G. S. Palayret, B. Lehner, L. Di Croce, A. Wutz, B. Hendrich, D. Klenerman, E. D. Laue, 3D structures of individual mammalian genomes studied by single-cell Hi-C. *Nature* **544**, 59–64 (2017).

128. V. B. Teif, J.-P. Mallm, T. Sharma, D. B. Mark Welch, K. Rippe, R. Eils, J. Langowski, A. L. Olins, D. E. Olins, Nucleosome repositioning during differentiation of a human myeloid leukemia cell line. *Nucleus* **8**, 188–204 (2017).

129. H. Zhou, M. Rosen, Dataset for chromatin condensate structure and dynamic analysis, Dryad (2025); DOI:10.5061/dryad.p2ngf1w45.

130. H. Zhou, M. Rosen, Software for Chromatin Condensate Structure Spatial Analysis, Zenodo (2025); DOI:10.5281/zenodo.16324927.

131. K. Russell, Chromatin Reconstruction and Steered Molecular Dynamics Code, Zenodo (2025); DOI:10.5281/zenodo.16331795.

132. G. Koulouras, A. Panagopoulos, M. A. Rapsomaniki, N. N. Giakoumakis, S. Taraviras, Z. Lygerou, EasyFRAP-web: a web-based tool for the analysis of fluorescence recovery after photobleaching data. *Nucleic Acids Research* **46**, W467–W472 (2018).

133. K. Kelley, A. M. Raczkowski, O. Klykov, P. Jaroenlak, D. Bobe, M. Kopylov, E. T. Eng, G. Bhabha, C. S. Potter, B. Carragher, A. J. Noble, Waffle Method: A general and flexible approach for improving throughput in FIB-milling. *Nat Commun* **13**, 1857 (2022).

134. D. Tegunov, P. Cramer, Real-time cryo-electron microscopy data preprocessing with Warp. *Nat Methods* **16**, 1146–1152 (2019).

135. S. Zheng, G. Wolff, G. Greenan, Z. Chen, F. G. A. Faas, M. Bárcena, A. J. Koster, Y. Cheng, D. A. Agard, AreTomo: An integrated software package for automated marker-free, motion-corrected cryo-electron tomographic alignment and reconstruction. *Journal of Structural Biology: X* **6**, 100068 (2022).

136. Y.-T. Liu, H. Zhang, H. Wang, C.-L. Tao, G.-Q. Bi, Z. H. Zhou, Isotropic reconstruction for electron tomography with deep learning. *Nat Commun* **13**, 6482 (2022).

137. T. Wagner, S. Raunser, The evolution of SPHIRE-crYOLO particle picking and its application in automated cryo-EM processing workflows. *Commun Biol* **3**, 61 (2020).

138. E. Moebel, A. Martinez-Sanchez, L. Lamm, R. D. Righetto, W. Wietrzynski, S. Albert, D. Larivière, E. Fourmentin, S. Pfeffer, J. Ortiz, W. Baumeister, T. Peng, B. D. Engel, C. Kervrann, Deep learning improves macromolecule identification in 3D cellular cryo-electron tomograms. *Nat Methods* **18**, 1386–1394 (2021).

139. J. Zivanov, J. Otón, Z. Ke, A. Von Kügelgen, E. Pyle, K. Qu, D. Morado, D. Castaño-Díez, G. Zanetti, T. A. Bharat, J. A. Briggs, S. H. Scheres, A Bayesian approach to single-particle electron cryo-tomography in RELION-4.0. *eLife* **11**, e83724 (2022).

140. T. A. M. Bharat, C. J. Russo, J. Löwe, L. A. Passmore, S. H. W. Scheres, Advances in Single-Particle Electron Cryomicroscopy Structure Determination applied to Sub-tomogram Averaging. *Structure* **23**, 1743–1753 (2015).

141. E. C. Meng, T. D. Goddard, E. F. Pettersen, G. S. Couch, Z. J. Pearson, J. H. Morris, T. E. Ferrin, UCSF ChimeraX: Tools for structure building and analysis. *Protein Science* **32**, e4792 (2023).

142. U. H. Ermel, S. M. Arghittu, A. S. Frangakis, ArtiaX: An electron tomography toolbox for the interactive handling of sub-tomograms in UCSF ChimeraX. *Protein Science* **31**, e4472 (2022).

143. J. Singh, J. M. Thornton, SIRIUS: An automated method for the analysis of the preferred packing arrangements between protein groups. *Journal of Molecular Biology* **211**, 595–615 (1990).

144. D. N. Mastronarde, S. R. Held, Automated tilt series alignment and tomographic reconstruction in IMOD. *Journal of Structural Biology* **197**, 102–113 (2017).

145. D. Farré-Gil, J. P. Arcon, C. A. Laughton, M. Orozco, CGeNArate: a sequence-dependent coarse-grained model of DNA for accurate atomistic MD simulations of kb-long duplexes. *Nucleic Acids Research* **52**, 6791–6801 (2024).

146. A. Jamakovic, P. Van Mieghem, "On the Robustness of Complex Networks by Using the Algebraic Connectivity" in *NETWORKING 2008 Ad Hoc and Sensor Networks, Wireless Networks, Next Generation Internet*, A. Das, H. K. Pung, F. B. S. Lee, L. W. C. Wong, Eds. (Springer Berlin Heidelberg, Berlin, Heidelberg, 2008; http://link.springer.com/10.1007/978-3-540-79549-0_16)vol. 4982 of *Lecture Notes in Computer Science*, pp. 183–194.

147. J. Schindelin, I. Arganda-Carreras, E. Frise, V. Kaynig, M. Longair, T. Pietzsch, S. Preibisch, C. Rueden, S. Saalfeld, B. Schmid, J.-Y. Tinevez, D. J. White, V. Hartenstein, K. Eliceiri, P. Tomancak, A. Cardona, Fiji: an open-source platform for biological-image analysis. *Nat Methods* **9**, 676–682 (2012).

148. T. Kuhn, J. Hettich, R. Davtyan, J. C. M. Gebhardt, Single molecule tracking and analysis framework including theory-predicted parameter settings. *Sci Rep* **11**, 9465 (2021).

149. J. C. M. Gebhardt, D. M. Suter, R. Roy, Z. W. Zhao, A. R. Chapman, S. Basu, T. Maniatis, X. S. Xie, Single-molecule imaging of transcription factor binding to DNA in live mammalian cells. *Nat Methods* **10**, 421–426 (2013).

150. S. Ide, S. Tamura, K. Maeshima, Chromatin behavior in living cells: Lessons from single-nucleosome imaging and tracking. *BioEssays* **44**, 2200043 (2022).

151. M. Tassieri, G. M. Gibson, R. M. L. Evans, A. M. Yao, R. Warren, M. J. Padgett, J. M. Cooper, Measuring storage and loss moduli using optical tweezers: Broadband microrheology. *Phys. Rev. E* **81**, 026308 (2010).

152. I. Alshareedah, M. M. Moosa, M. Pham, D. A. Potoyan, P. R. Banerjee, Programmable viscoelasticity in protein-RNA condensates with disordered sticker-spacer polypeptides. *Nat Commun* **12**, 6620 (2021).

153. T. G. Mason, D. A. Weitz, Optical Measurements of Frequency-Dependent Linear Viscoelastic Moduli of Complex Fluids. *Phys. Rev. Lett.* **74**, 1250–1253 (1995).

154. R. M. L. Evans, M. Tassieri, D. Auhl, T. A. Waigh, Direct conversion of rheological compliance measurements into storage and loss moduli. *Phys. Rev. E* **80**, 012501 (2009).

155. T. G. Mason, Estimating the viscoelastic moduli of complex fluids using the generalized Stokes–Einstein equation. *Rheologica Acta* **39**, 371–378 (2000).

156. V. V. Rusakov, Yu. L. Raikher, R. Perzynski, Brownian Motion in the Fluids with Complex Rheology. *Math. Model. Nat. Phenom.* **10**, 1–43 (2015).

157. P.-Y. Kan, T. L. Caterino, J. J. Hayes, The H4 Tail Domain Participates in Intra- and Internucleosome Interactions with Protein and DNA during Folding and Oligomerization of Nucleosome Arrays. *Molecular and Cellular Biology* **29**, 538–546 (2009).

158. C. Tse, J. C. Hansen, Hybrid Trypsinized Nucleosomal Arrays: Identification of Multiple Functional Roles of the H2A/H2B and H3/H4 N-Termini in Chromatin Fiber Compaction. *Biochemistry* **36**, 11381–11388 (1997).

159. S. C. Moore, J. Ausió, Major Role of the Histones H3-H4 in the Folding of the Chromatin Fiber. *Biochemical and Biophysical Research Communications* **230**, 136–139 (1997).

160. C. Zheng, X. Lu, J. C. Hansen, J. J. Hayes, Salt-dependent Intra- and Internucleosomal Interactions of the H3 Tail Domain in a Model Oligonucleosomal Array. *Journal of Biological Chemistry* **280**, 33552–33557 (2005).

161. P.-Y. Kan, X. Lu, J. C. Hansen, J. J. Hayes, The H3 Tail Domain Participates in Multiple Interactions during Folding and Self-Association of Nucleosome Arrays. *Molecular and Cellular Biology* **27**, 2084–2091 (2007).

162. S. Pepenella, K. J. Murphy, J. J. Hayes, A Distinct Switch in Interactions of the Histone H4 Tail Domain upon Salt-dependent Folding of Nucleosome Arrays. *Journal of Biological Chemistry* **289**, 27342–27351 (2014).

163. B. R. Macadangdang, A. Oberai, T. Spektor, O. A. Campos, F. Sheng, M. F. Carey, M. Vogelauer, S. K. Kurdistani, Evolution of histone 2A for chromatin compaction in eukaryotes. *eLife* **3**, e02792 (2014).

164. A. Allahverdi, R. Yang, N. Korolev, Y. Fan, C. A. Davey, C.-F. Liu, L. Nordenskiöld, The effects of histone H4 tail acetylations on cation-induced chromatin folding and self-association. *Nucleic Acids Research* **39**, 1680–1691 (2011).

165. X. Wang, J. J. Hayes, Acetylation Mimics within Individual Core Histone Tail Domains Indicate Distinct Roles in Regulating the Stability of Higher-Order Chromatin Structure. *Molecular and Cellular Biology* **28**, 227–236 (2008).

166. D. Sinha, M. A. Shogren-Knaak, Role of Direct Interactions between the Histone H4 Tail and the H2A Core in Long Range Nucleosome Contacts. *Journal of Biological Chemistry* **285**, 16572–16581 (2010).
